# Ring Protonation Regulates the Homogeneous Electrocatalytic Oxygen Reduction Reaction Mediated by Manganese Phthalocyanine

**DOI:** 10.1021/jacs.5c14858

**Published:** 2025-11-21

**Authors:** Mary Jo McCormick, Kate G. Scheerer, Charles W. Machan

**Affiliations:** Department of Chemistry, 744402 University of Virginia, PO Box 400319, Charlottesville, Virginia 22904-4319, United States

## Abstract

The development of transition-metal based catalysts for small-molecule activation is essential to the development of viable alternative energy sources to nonrenewable fossil fuels. Herein, the synthesis and characterization of Mn­(^tBu^phthalocyanine)Cl (**1Cl**) as a homogeneous catalyst for the oxygen reduction reaction (ORR) with halogenated acetic acid derivatives is reported. The activity and selectivity of **1Cl** was studied electrochemically and spectrochemically. Interestingly, the activity of **1Cl** as a catalyst for the ORR displays an inverse correlation with the strength of the acid. Mechanistic studies suggest that this occurs because ring protonation leads to a deactivated form of the catalyst and ultimately degradation, limiting the strength of the proton donor which may be used. These observations contrast studies on previously reported analogous Fe-based phthalocyanines, where protonation has been suggested to improve activity. The results with the Mn complex imply that an understanding of ligand basicity and resultant control over the reaction environment is generally useful for the catalytic properties of this class of macrocyclic complexes in homogeneous configurations.

## Graphical abstract

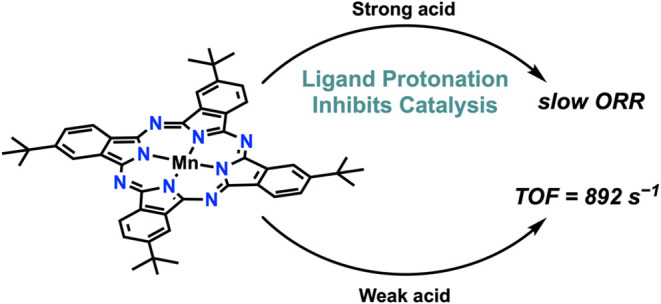

## Introduction

Due to the harmful effects of the increasing concentration of greenhouse gases in the atmosphere,^1^ the development of energy sources that do not rely on the burning of fossil fuels is imperative. Presently, the most plausible alternative energy sources rely on the electrochemical activation of abundant small molecules, such as dioxygen (O_2_). There are two possible pathways for the oxygen reduction reaction (ORR): a 4-proton (H^+^)/4-electron (e^–^) pathway to produce two equivalents of water and a 2H^+^/2e^–^ pathway to produce a single equivalent of hydrogen peroxide (H_2_O_2_).^2,3^ Both processes are desirable from an environmental standpoint, as the reduction of O_2_ to H_2_O is an ideal cathodic half-reaction in a water-splitting fuel cell, and the reduction of O_2_ to H_2_O_2_ could provide a more environmentally friendly method to produce H_2_O_2_, a commodity chemical used at large scale in multiple industrial processes.^4^

The abundance of N_4_-metallomacrocyclic complexes that bind and activate O_2_ in nature has inspired the investigation into synthetic N_4_-metallomacrocycles, such as porphyrins, corroles, and phthalocyanines, as catalysts for the ORR.^5^ Metallophthalocyanines (MPc) have unique structural aspects compared to other N_4_-metallomacrocycles, including an extended aromatic π-system that allows for rapid redox processes and the presence of basic nitrogen atoms that allow for facile ligand protonation.^6,7^ Ligand protonation has been shown to control spectrochemical properties in MPcs previously.^3,7–9^ Since ligand protonation induces dramatic changes in electronic structure, it can be monitored by UV–vis spectroscopy: protonation of the azomethine nitrogen by the addition of acid results in a bathochromic (or red) shift of the MPc Q-band.^8^ The addition of acid can also result in the demetalation of the MPc, with the stability of the MPc primarily depending on the identity of the metal center.^8,10^

Since the protonation of the MPc ligand results in distinct changes in the electronic structure, the effect of ligand protonation on the catalytic abilities of MPcs is also of interest. Previously, study of the per-arylated MPc Co^II^(Ph_8_Pc), where Ph_8_Pc = α-octaphenylphthalocyaninato, as a catalyst for the ORR revealed that ligand protonation inhibited the direct reduction of oxygen under acidic conditions through the Co^III/II^ redox couple. However, in the presence of a sufficiently strong reducing agent, ligand protonation enabled an alternative proton-coupled electron transfer (PCET) mechanism to produce a catalytically active Co^I^(Ph_8_PcH) species, where H is located on an azomethine in the ligand.^7^

FePc also shows catalytic dependence on protonation in acidic media.^9,11^ In a porphyrinic framework, it has previously been assessed that Mn centers should bind O_2_ more strongly than Fe.^12,13^ Interested in the consequences of ring protonation on such comparisons, as well as the possible differences in ORR activity between metalloporphyrins and MPcs with manganese active sites,^14–17^ the *tert*-butylated derivative **1Cl** was examined.^18^ Here, mechanistic studies in acetonitrile (MeCN) show rapid catalysis of the ORR by **1Cl** in the presence of acids too weak to protonate the ligand framework (monochloroacetic acid, ClAcOH) and degradation of **1Cl** in the presence of acids strong enough to protonate the ligand framework (2,2,2-trifluoroacetic acid, TFAH).

## Results

### Synthesis and Characterization

The synthesis of Mn­(^tBu^phthalocyanine)­Cl, **1Cl**, was conducted according to a literature precedent.^19^ A mixture of MnCl_2_·4H_2_O and *tert*-butylphthalonitrile was suspended in minimal MeOH and heated until a molten flux was achieved (250 °C). After 2 h, the flux was allowed to cool and the resulting dark solid was then crushed and sequentially boiled in 1 N HCl and 1 N NaOH for 1 h before isolation on filter paper with a Buchner funnel with applied vacuum. The resulting dark green solid was spectroscopically pure and was characterized by elemental analysis, as well as NMR and UV–vis spectroscopies.

### Spectrochemical Analysis of Ligand Protonation

To establish the conditions required for ligand protonation, acetic acid derivatives in acetonitrile (MeCN) with varying proton activity were analyzed spectroscopically by UV–vis titration in the presence of **1Cl**. The addition of TFAH [p*K*_a_ (MeCN) = 12.65][Bibr ref20] was sufficient to induce the formation of a new species, as indicated by the presence of isosbestic points (Figure 1A). With the addition of TFAH as a strong acid, an absorbance band at 712 nm decreases as new bands at 543 and 740 nm increase in intensity. A Hill Plot (Figure 1B) using the absorbance at 740 nm gives a linear slope indicative of noncooperative binding.^11^ A protonation equilibrium constant, *K*_eq_, involving the aza-N-bridge in the phthalocyanine ligand was determined to be 76 M^–1^ (Figure 1C). Since contributing factors to the equilibrium constant undoubtedly include both the coordination of the conjugate base trifluoroacetate to Mn as well as the homoconjugation of TFAH with trifluoroacetate (p*K*_f_ = 3.9, where *K*_f_ is defined as HA + A^–^ ⇌ HA_2_^–^)^3^, 2,6-lutidinium tetrafluoroborate (p*K*_a_ (MeCN) = 14.1)^21^ was also used for titration study. As an acid source, 2,6-lutidinium tetrafluoroborate is expected to be less prone to homoconjugation (for unfunctionalized pyridinium p*K*_f_ (MeCN) = 0.8)^22^ and too sterically hindered to coordinate to the Mn center. Spectral shifts consistent with azomethine protonation are again observed: the resultant Hill Plot using the absorbance at 740 nm gives a linear slope and a protonation equilibrium constant, *K*_eq_, was determined to be 107 M^–1^ (Figure S2). Assuming minimal contributions from the secondary factors described above, the p*K*_a_ of the conjugate acid of **1Cl** can be approximated as 16.1 from the 2,6-lutidinium titration study.

**Figure 1:**
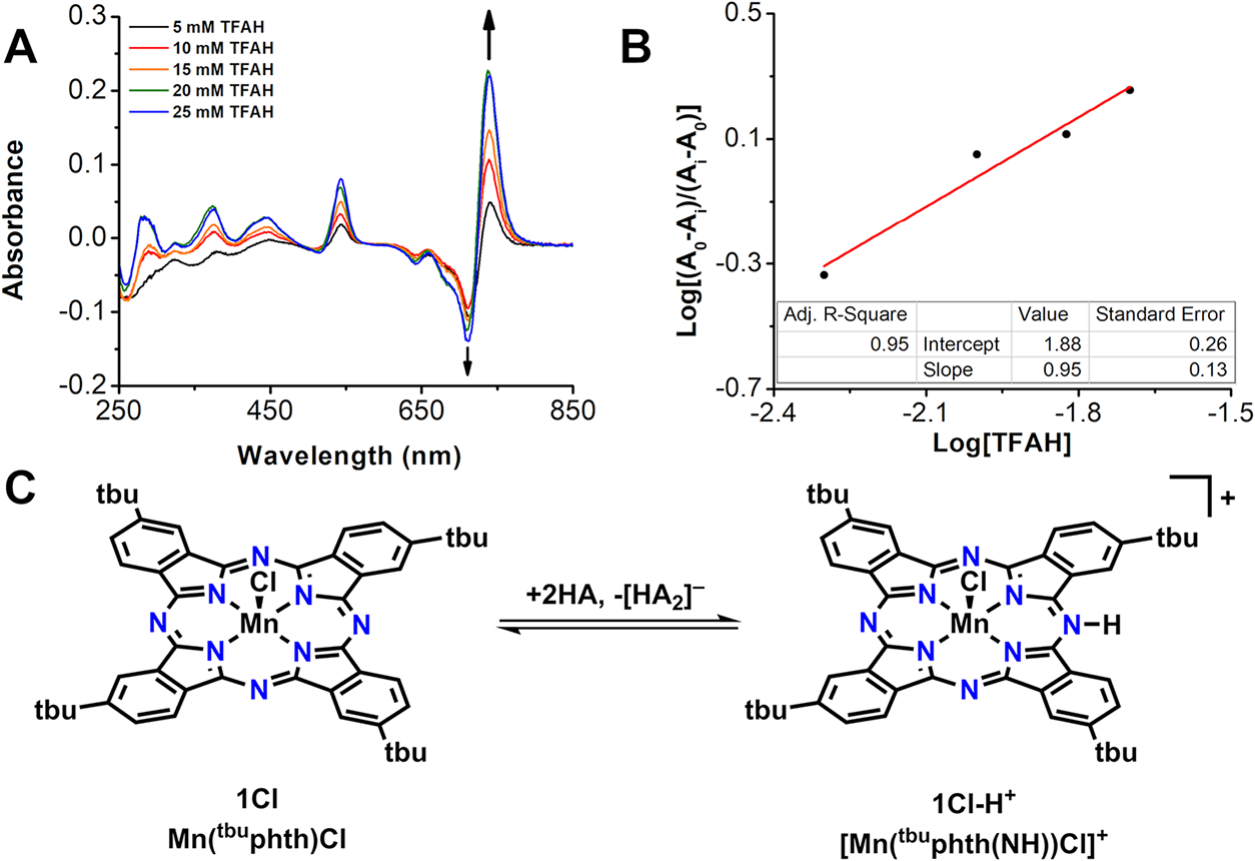
(A) UV–vis difference spectra of **1Cl** (25 μM) in MeCN with increasing amounts of TFAH. (B) Hill Plot obtained from absorbance changes at 740 nm. (C) Scheme depicting the proposed species involved in the protonation of [Mn­(Pc)]^+^, omitting possible chloride equilibria.

The initial additions of an acid of intermediate strength, dichloroacetic acid, Cl_2_AcOH [p*K*_a_ (MeCN) = 17.3],^20^ to a solution of **1Cl** (25 μM) result in a minimal shift in the absorbance spectra and did not indicate protonation at low acid concentrations (Figure S3). However, subsequent additions to reach a concentration of 25 mM Cl_2_AcOH did result in the formation of the protonated species (**1Cl–H** ^+^, Figure 1C) as indicated by the increase in absorbance again at 543 and 740 nm. This variable protonation behavior at low and high concentrations of Cl_2_AcOH is indicative of near equilibrium conditions for protonation, consistent with the estimated ligand p*K*_a_ of 16.1.

The addition of 25 mM of the relatively weaker acid ClAcOH [p*K*_a_ (MeCN) = 20.3][Bibr ref20] yields a slight decrease in the absorbances of the MPc overall but does not result in the appearance of the spectrochemical handles that are consistent with protonation (Figure S4). The absence of protonation even at large excess is indicative of being far away from the ligand protonation equilibrium. Based on these observations, it was reasoned that addition of conjugate base could be used to attenuate proton activity in solution with a stronger acid, shifting the system away from ligand protonation. Indeed, the addition of 1:1 buffered Cl_2_AcOH/sodium dichloroacetate (NaCl_2_AcO) does not result in the absorbance shift associated with ligand protonation at concentrations where the unbuffered acid would. However, addition of buffer at these concentrations does result in an increase in absorbance of the strong Q-band at 718 nm, which is ascribed to improved catalyst dispersion (Figure S5).^23^ Control studies confirmed that the dispersion is not a result of the Na^+^ ion that is introduced with the conjugate base interacting with the complex (Figure S6). Further, addition of NaTFA and tetrabutylammonium acetate both show comparable increases in absorbance consistent with dispersion (Figures S7 and S8); the conjugate acids of these bases are strong enough and too weak to protonate the ligand, respectively. Overall, these observations across different acid activities are consistent with the estimated p*K*_a_ of the aza ring bridge.

### Electrochemical Analysis

Having established the conditions required for ligand protonation and that the inclusion of conjugate base could shift the equilibrium away from protonation, electrochemical studies were conducted to compare catalytic activity in the protonated state, at near equilibrium to protonation, and in the unprotonated state. Initially, complex **1Cl** was analyzed by cyclic voltammetry (CV) in MeCN with tetrabutylammonium hexafluorophosphate (TBAPF_6_) as the supporting electrolyte. Under inert Ar atmosphere, there is a single reversible one-electron reduction feature at *E*_1/2_ = −0.58 V vs Fc^+^/Fc. The current density at this feature was found to increase linearly with the square root of the scan rate between 0.025 and 5 V/s, indicating a diffusion-limited response suggestive of a homogeneous species.^24^ The diffusion coefficient was calculated from the slope of the plot using the Randles-Ševčík equation and found to be 5.98 × 10^–6^ cm^2^·s^–1^ (See Supporting Information, Figure S17).

Upon saturation with dioxygen (O_2_), the reversible feature is replaced with two, irreversible, one-electron reductions at *E*_p_ = −0.58 V vs Fc^+^/Fc and *E*_p_ = −0.67 V vs Fc^+^/Fc that coalesce into a single wave with an *E*_p_ = −0.61 V vs Fc^+^/Fc at and above scan rates of 200 mV/s (Figure S18). The coalescence of these peaks is accompanied by the appearance of a weak oxidation feature at *E*_p_ = −0.30 V vs Fc^+^/Fc that disappears at a scan rate of 4 V/s. The Mn^III/II^ feature becomes reversible again as higher scan rates (2–10 V/s) are reached. The slope of the log of the scan rate versus the shift in reduction potential is −16.5, which is close to the slope of −19.7 for RRD or RSD-DISP2 mechanisms, suggesting that dimerization reactions involving Mn-superoxide species are possible in the absence of a proton donor (Figure S18).^25^ Comparing the log of O_2_ concentration to the shift in peak reduction potential for the first wave shows a slope of 12.7, which aligns better with an RRD mechanism (Figure S19).

The addition of TFAH (50 mM) under Ar saturation results in a positive shift of the Mn^III/II^ reduction feature to *E*_p_ = −0.34 V vs Fc^+^/Fc, a loss of reversibility, and an increase in current density (Figure S20). Based on the spectrochemical data described above, it is proposed that this reduction feature corresponds to the protonated Mn species (**1Cl–H** ^**+**^, Figure 1C). There is minimal change to the feature observed with added TFAH upon saturation with O_2_: the feature shifts to a slightly more negative peak potential *E*_p_ = −0.35 V vs Fc^+^/Fc, and a minimal increase in current density is observed. The absence of a significant current increase upon saturation with O_2_ indicates that electrocatalytic reduction of oxygen by the **1Cl** precatalyst is not occurring in the presence of excess TFAH. Thus, ring protonation is observed to inhibit electrocatalytic ORR behavior.

Similarly, the addition of Cl_2_AcOH (50 mM) under Ar saturation results in a positive shift for the redox response of **1Cl** to *E*_p_ = −0.50 V vs Fc^+^/Fc, some loss of reversibility, and an increase in current density; however, the changes are accompanied by the appearance of a prefeature with *E*_p_ = −0.36 V vs Fc^+^/Fc. Upon saturation with O_2_, there is a slightly negative shift to *E*_cat/2_ = −0.56 V vs Fc^+^/Fc and a total loss of reversibility that is accompanied by an increase in current density, which is consistent with the electrocatalytic ORR by **1Cl** (Figure 2). Interestingly, variable concentration studies indicate an inverse mixed-order dependence of the observed catalytic current density on Cl_2_AcOH concentration; the decrease in current density with increasing acid concentrations is also accompanied by a positive shift in potential (Figure S21). Based on these results and given that features consistent with protonation of **1Cl** were only visible at elevated concentrations of Cl_2_AcOH, subsequent studies analyzed both low acid (20-fold excess) and high acid (100-fold excess) regimes. Doing so enabled a performance comparison of the catalyst primarily in the unprotonated state with conditions where the catalyst was predominantly in the protonated state. There was an observed first-order dependence on **1Cl** and O_2_ concentrations in both the low and high acid regimes (Figures S22–S27).

**Figure 2:**
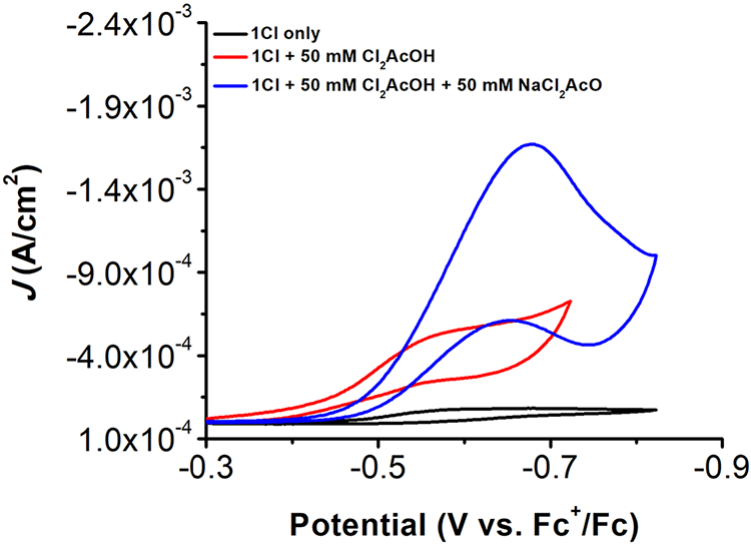
CVs of **1Cl** showing the increase in catalytic activity upon the buffering of the high Cl_2_AcOH acid regime. Conditions: 0.5 mM **1Cl** (black) with 50 mM Cl_2_AcOH (red) and 50 mM 1:1 Cl_2_AcOH/Cl_2_AcO^–^ buffer (blue) in oxygen-saturated 0.1 M TBAPF_6_/MeCN; glassy carbon working electrode, glassy carbon rod counter electrode, Ag/AgCl pseudoreference electrode; referenced to Fc^+^/Fc internal standard; 100 mV/s scan rate.

The addition of conjugate base, NaCl_2_AcO, to create a 1:1 buffer (50 mM) shifts the catalytic feature from *E*_cat/2_ = −0.56 V vs Fc^+^/Fc to *E*_cat/2_ = −0.68 V vs Fc^+^/Fc and results in a dramatic increase in current density (Figure 2). As was the case during UV–vis testing, control studies using NaPF_6_ as an alternate Na^+^ source suggest the enhancement of the catalytic activity is due to the addition of the conjugate base and not Lewis Acid interactions (Figure S32). Thus, the role of the base in attenuating proton activity seems to be primarily to preserve the more active unprotonated form of the catalyst. Variable concentration studies with a 1:1 buffer indicate an inverse mixed-order dependence of catalysis on the buffer concentration; a 30 mM concentration of 1:1 buffer was chosen for use in later analyses since this concentration resulted in the greatest current density (Figure S28). Mechanistic studies revealed a first-order dependence on oxygen and half-order dependence on catalyst at this concentration of buffer (Figures S29, S33, and S34). With a constant concentration of acid (30 mM), a mixed-order dependence on conjugate base was also observed, consistent with Mn coordination and proton activity being sensitive to this reagent (Figures S30 and S31). Rotating Ring Disk Voltammetry (RRDV) revealed an electrochemical product selectivity of 100 ± 1.8% H_2_O for the buffered system (Table 1 and Figure S35). The Koutecky–Levich analysis (Figure S36) for the buffered system showed a rotation-rate dependence for the kinetic current (K-L intercept), implying interference from an intermediate chemical step.^26^ Based on the kinetic experiments showing less than first-order concentration dependence, it is assumed that the dimerization of **1Cl** is relevant on the time scale of these experiments. In other words, at slower rotation rates, the effect of the dimerization reaction can be observed as a smaller kinetic current value. At faster rotation rates, the dimerization reaction has a relatively smaller contribution, resulting in larger kinetic current. Unfortunately, the electrochemical selectivities of the unbuffered system under high and low acid-concentrations of Cl_2_AcOH were unable to be accurately assessed due to electrode fouling by redox products of the reaction.

**Table 1:** Summary of Product Quantification of ORR Catalyzed by **1Cl** ^a^

	electrochemical selectivity	spectrochemical selectivity
**ClAcOH**	59.5 ± 6.4% H_2_O_2_	96.5 ± 9.9% H_2_O_2_
**Cl**_**2**_ **AcOH**	-	99 ± 6.6% H_2_O_2_
**buffer**	0.04 ± 1.8% H_2_O_2_	102 ± 6.7% H_2_O_2_

a “-“ for less than quantitative efficiency for H_2_O_2_, H_2_O is assumed to be the ORR product.

The addition of the weakest acid ClAcOH (50 mM) under Ar-saturated conditions with **1Cl** resulted in a minimal shift of *E*_1/2_ = −0.58 V vs Fc^+^/Fc to *E*_1/2_ = −0.57 V vs Fc^+^/Fc, consistent with the previous postulation that protonation of the ligand is not occurring appreciably. Under otherwise comparable conditions with O_2_-saturation, the addition of acid results in a total loss of reversibility and shift to *E*_cat/2_ = −0.74 V vs Fc^+^/Fc that is accompanied by an increase in current density, which is indicative of the electrocatalytic ORR mediated by **1Cl**. Variable concentration studies revealed a first-order dependence of the observed rate on ClAcOH and oxygen as well as a mixed-order dependence on catalyst (Figures S37–S40). An electrochemical product selectivity of 59.5 ± 6.4% H_2_O_2_ was determined through RRDV (Table 1and Figure S42). The Levich and Koutecky–Levich analyses (Figure S43) indicate rotation rate dependence for the kinetic current in this system, as well, consistent with a dimerization reaction occurring on the time scale of the experiment. The turnover frequency (TOF) for the ORR with ClAcOH as the proton donor was calculated to be 892 s^–1^ at a scan rate of 2 V/s with ClAcOH as the proton donor (Figure S41) using the ratio of catalytic current to Faradaic current.^24^

### UV–vis Spectroelectrochemical Analysis

In order to reconcile the protonation observed by UV–vis titration with the redox shifts observed by CV, the speciation of **1Cl** at applied potential was subsequently analyzed by UV–vis spectroelectrochemical analysis (UV–vis-SEC) in MeCN with TBAPF_6_ as the supporting electrolyte. Under inert atmosphere, there are distinct spectral shifts in the spectrochemical absorption spectrum that correspond to the formal reduction of aprotic Mn^III^ to Mn^II^: first, the λ_max_ of the Q-band at 718 nm (A) associated with the Mn^III^ complex decreases as a new feature appears at λ_max_ = 678 nm (Figure 3A). This is accompanied by the appearance of a shoulder (B) at λ_max_ = 616 nm and a blue-shift of a low-intensity band (C) from λ_max_ = 537 nm to λ_max_ = 532 nm. The Soret band (D) at λ_max_ = 365 nm is also blue-shifted to 347 nm. In the presence of air, the aprotic spectra show slight differences: band (A) at λ_max_ = 718 nm also shifts to λ_max_ = 678 nm, which is accompanied by the appearance of band (B) at λ_max_ = 619 nm. However, in the presence of air, the band C at λ_max_ = 529 nm diminishes in intensity at reducing potentials. Finally, band D shifts from λ_max_ = 364 nm to λ_max_ = 340 nm **(** Figure 3 **B)**.

**Figure 3:**
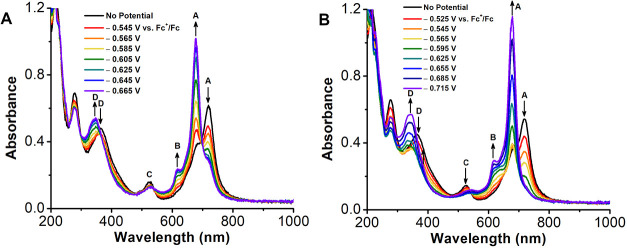
UV–vis showing spectral changes of **1Cl** under (A) inert atmosphere and (B) ambient air. Conditions: 86 μM **1Cl** in 0.1 M TBAPF_6_/MeCN, Au honeycomb working and counter electrode, leakless mini KCl reference electrode, references to external Fc^+^/Fc standard.

In the presence of TFAH, where protonation was observed to occur by UV–vis titration, the initial absorbance features of the protonated Mn^III^ complex are all red-shifted compared to the aprotic conditions (Figures S9 and S10). Reduction of **1Cl** in the presence of TFAH results in the decomposition of the complex under both inert and ambient conditions, as evidenced by a bleaching of all spectral features (see Table S1 for all peak λ_max_). Similarly, the reduction of Mn^III^ in the presence of 100 equiv of Cl_2_AcOH (Figures 4B and S12), where the system was shown to slightly favor protonation in UV–vis studies, results in a similar apparent decomposition of a portion of the complex; all peaks decrease in intensity but do not shift in wavelength. This is consistent with the incomplete protonation at elevated acid concentrations observed during UV–vis studies, *vide supra*.

**Figure 4:**
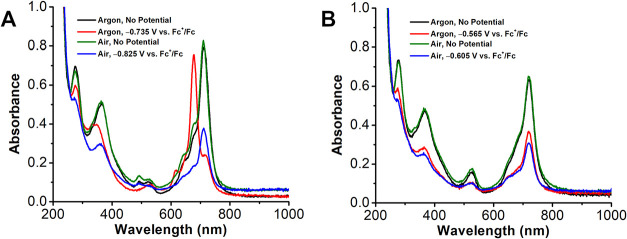
Comparison of the initial **1Cl** solution with the fully reduced solution with (A) 20 eq. Cl_2_AcOH and (B) 100 eq. Cl_2_AcOH. Conditions: 86 μM **1Cl** and (A) 1.7 mM and (B) 8.6 mM Cl_2_AcOH in 0.1 M TBAPF_6_/MeCN, Au honeycomb working and counter electrode, leakless mini KCl reference electrode, references to external Fc^+^/Fc standard.

In the presence of only 20 equiv of Cl_2_AcOH (Figures 4A and S11) where protonation was observed to be less favorable by UV–vis, there are noteworthy differences compared to the UV–vis SEC data collected with TFAH or 100 equiv of Cl_2_AcOH. Under inert atmosphere with 20 equiv of Cl_2_AcOH, the band (A) at λ_max_ = 712 nm does not fully disappear upon reduction; instead, it splits into two bands at λ_max_ = 718 nm and λ_max_ = 678 nm and is accompanied by the appearance of band (B) at λ_max_ = 615 nm. Band (C) is split into two peaks at λ_max_ = 493 nm and λ_max_ = 523 nm before reduction which coalesce into one band at λ_max_ = 528 nm upon reduction. The single band, (D), at λ_max_ = 366 nm splits into two bands at λ_max_ = 333 nm and λ_max_ = 348 nm upon reduction. Under air with 20 equiv of Cl_2_AcOH, the band (A) at λ_max_ = 710 nm does not shift or split upon reduction. Band (B) is present at λ_max_ = 641 nm before reduction and disappears with reduction. Band (C) is also split in these conditions, resulting in two peaks at λ_max_ = 492 nm and λ_max_ = 526 nm that coalesce into one peak at λ_max_ = 491 nm upon reduction. Band (D) does not split under ambient conditions; instead, it slightly blue-shifts from λ_max_ = 363 nm to λ_max_ = 360 nm.

The addition of ClAcOH, which was too weak to protonate the ligand at any concentration in UV–vis studies, under inert atmosphere results in the splitting of band (A) from λ_max_ = 718 nm to λ_max_ = 713 nm and λ_max_ = 680 nm upon reduction, similar to what is observed with 20 equiv of Cl_2_AcOH (Figures S15 and S16). However, there is no appearance of band (B) associated with this reduction, band (C) at λ_max_ = 526 nm does not shift, and there is a slight blue-shift of band (D) from λ_max_ = 366 nm to λ_max_ = 359 nm. Similar again to the principal peaks with low concentrations of Cl_2_AcOH, when air and ClAcOH are present, the band (A) at λ_max_ = 718 nm remains and band (B) at λ_max_ = 680 nm appears upon reduction. Band (C) at λ_max_ = 526 nm also remains unchanged upon reduction under ambient conditions, and band (D) at λ_max_ = 364 nm is blue-shifted to 361 nm. Overall, these results suggest that reduction following protonation results in the degradation of **1Cl**, inhibiting catalysis, and that this occurs primarily with high concentrations of Cl_2_AcOH or stronger acids.

### Spectrochemical Analysis

As a supplement to electrochemical experiments, where the reaction selectivity could not be determined for all conditions because of electrode fouling, spectrochemical experiments were conducted where the concentration of catalyst could be significantly lowered. Thus, the ORR activity of **1Cl** was next examined using UV–vis stopped-flow techniques with decamethylferrocene (Cp*_2_Fe) as the sacrificial reductant. The spectral handle of the oxidized [Cp*_2_Fe]^+^ at 780 nm was tracked over time and used to monitor the rate of the reaction. The observed rate law for each acid was determined by varying the concentrations of the reactants (**1Cl**, HA, O_2_, and Cp*_2_Fe). With the Cl_2_AcOH/Cl_2_AcO^–^ buffer (1:1 ratio), which should position the system slightly below the equilibrium position for protonation, there are observed first-order concentration dependences for reaction rate with respect to O_2_ and **1Cl**; zero-order concentration dependences are observed with respect to reductant and buffer (eq 1 and Figures S54–S55). With ClAcOH, the weakest acid, there is also a first-order concentration dependence for the observed rate on O_2_ and **1Cl**. There is a zero-order dependence on acid, and there are two regions with respect to reductant when ClAcOH was the added acid: a first-order dependence at high Cp*_2_Fe concentrations (1.33 to 2 mM) and a zero-order dependence at low concentrations (eq 2 and Figures S44–S48). All other experiments were conducted in the zero-order dependence region of Cp*_2_Fe concentration. Variable-temperature kinetic studies were conducted with the buffered system to investigate the relative enthalpic and entropic contributions to the transition state. Using Eyring analysis, the entropic parameter was found to be negative (−5.49 J mol^–1^ K^–1^) under these conditions, consistent with a bimolecular association step, *vide infra* (Figure S59). Based on the observed first-order concentration dependences of **1Cl** and O_2_, this suggests that the rate-determining step under these conditions is O_2_ binding to a Mn­(II) resting state formed through pre-equilibrium reduction. In the presence of Cl_2_AcOH, conditions where protonation is implicated with larger amounts of acid present, there is a first-order concentration dependence of reaction rate on **1Cl** and an overall inverse dependence on acid which showed a variable quantity based on the amount of added acid, precluding a meaningful overall fit. Interestingly, there is a zero-order concentration dependence for the observed rate on O_2_ and reductant when Cl_2_AcOH is the added acid (eq 3 and Figures S49–S53).

1 $${r\mathrm{ate}}_{\mathrm{Cl}2\mathrm{AcOH}/\mathrm{Cl}2\mathrm{AcO}-}=k_{\mathrm{cat}}{\left[1\mathrm{Cl}\right]}^{1}{[O_{2}]}^{1}$$

2 $${r\mathrm{ate}}_{\mathrm{ClAcOH}}=k_{\mathrm{cat}}{\left[1\mathrm{Cl}\right]}^{1}{\left[O_{2}\right]}^{1}{\left[{\mathrm{Cp}}_{2}^{*}\mathrm{Fe}\right]}^{\mathrm{variable}}$$

3 $${r\mathrm{ate}}_{\mathrm{Cl}2\mathrm{AcOH}}=k_{\mathrm{cat}}{\left[1\mathrm{Cl}\right]}^{1}{\left[{\mathrm{Cl}}_{2}\mathrm{AcOH}\right]}^{\mathrm{inverse}}$$

The spectrochemical selectivity for the ORR by **1Cl** was determined using a Ti­(O)­SO_4_ colorimetric assay (Figure S60).^4,20,27–29^ The spectrochemical reaction selectivity for ORR with all acids (ClAcOH, Cl_2_AcOH, and Cl_2_AcOH/Cl_2_AcO^–^ buffer) was determined to be quantitative for H_2_O_2_ within the error of the experiment. Specifically, the selectivity was 96.5 ± 9.9% H_2_O_2_ with ClAcOH, 99.9 ± 6.6% H_2_O_2_ with Cl_2_AcOH, and 102.9 ± 6.7% H_2_O_2_ with the buffer (Table 1 and, Figures S61–S63). Analysis for H_2_O_2_ degradation showed slight activity in the presence of ClAcOH and Cl_2_AcOH and moderate activity in the presence of the buffer (Figures S64–S66). These results imply that the most active catalyst form, the unprotonated product of single-electron reduction, has a selectivity independent of acid strength in this regime. Since the ClAcOH, Cl_2_AcOH, and Cl_2_AcOH/Cl_2_AcO^–^ buffer conditions showed a first-order dependence on **1Cl** concentration (Figures S45, S50, and S55), spectrochemical TOFs (reaction rate per equivalent of catalyst) could be determined for these conditions: 3.63 × 10^4^ s^–1^ with ClAcOH, 1.13 × 10^6^ s^–1^ for Cl_2_AcOH, and 6.75 × 10^4^ s^–1^ with the Cl_2_AcOH/Cl_2_AcO^–^ buffer as the added acid (See Supporting Information). These values show a direct relationship between TOF and acid strength, although the lack of a unified rate law makes further analysis specious.

### Computational Analysis

Computational studies were conducted to better understand the effects of ligand protonation on thermodynamic and kinetic contributions to the changes in the catalytic activity observed for **1Cl**. Geometry optimization was done with the Gaussian 16 package^30^ at B3LYP-D3­(BJ)/def2-SVP level^31–38^ with a complete structural model (only one isomer from the statistical mixture was used), and single point calculations for refining energy differences were completed with Orca 6.0 ^39–45^ at the ωB97M-D4/def2-TZVPPD^36,46–51^ level. The proton transfer thermochemistry considers the effect of homoconjugation, where proton transfer is accompanied by a favorable equilibrium interaction that results in the association of the conjugate base with another equivalent of acid (A^–^ + HA ⇌ HA_2_^–^).^20,52^ The protonation of **1Cl** to [Mn­(L­(NH))­Cl]^+^ is an endergonic reaction for all acids and becomes less favorable as p*K*_a_ increases: with TFAH, a Δ*G* of 2.4 kcal/mol; with Cl_2_AcOH, Δ*G* of 8.5 kcal/mol; and a Δ*G* of 13.8 kcal/mol for protonation of **1Cl** with ClAcOH. Here, L represents the tetracoordinate, *tert*-butyl phthalocyanine ligand for simplicity, since this coordination mode was unchanged in all structures, with NH representing protonation of the azomethine position. Considering the ligand protonation of the one-electron reduction product with accompanying chloride anion loss [Mn­(L)]^0^ to form­[Mn­(L­(NH))]^+^, the calculated thermodynamic parameters shift more negative: with TFAH, a Δ*G* of –6.0 kcal/mol; with Cl_2_AcOH, a Δ*G* of 0.1 kcal/mol; and with ClAcOH a Δ*G* of 5.4 kcal/mol (Table 2).

**Table 2:** Summary of DFT Results on Selected Reactions

reaction	Δ*G* ^‡^ (kcal/mol)	Δ*G* (kcal/mol)	*E* ^0^ (vs Fc^+/0^)
**1Cl** + 2TFAH ⇌ [**1Cl**-H]^+^ + [(TFA)_2_H]^−^		2.4	
**1Cl** + 2Cl_2_AcOH ⇌ [**1Cl**-H]^+^ + [(Cl_2_AcO)_2_H]^−^		8.5	
**1Cl** + 2ClAcOH ⇌ [**1Cl**-H]^+^ + [(ClAcO)_2_H]^−^		13.8	
Mn(L) + 2TFAH ⇌ [Mn(L(NH))]^+^ + [(TFA)_2_H]^−^		–6.0	
Mn(L) + 2Cl_2_AcOH ⇌ [Mn(L(NH))]^+^ + [(Cl_2_AcO)_2_H]^−^		0.1	
Mn(L) + 2ClAcOH ⇌ [Mn(L(NH))]^+^ + [(ClAcO)_2_H]^−^		5.4	
Mn(L) + O_2_ ⇌ Mn(L)(O_2_)	14.5	2.5	
[Mn(L(NH))]^+^ + O_2_ ⇌ [Mn(L(NH))(O_2_)]^+^	48.8	9.0	
Mn(L)(O_2_) + 2Cl_2_AcOH + e^–^ ⇌ Mn(L(O_2_H)) + [(Cl_2_AcO)_2_H]^−^		–12.6	0.55
Mn(L)(O_2_) + 2ClAcOH + e^–^ ⇌ Mn(L(O_2_H)) + [(ClAcO)_2_H]^−^		–7.3	0.32
Mn(L)(O_2_) + [Cl_2_AcO]^−^ ⇌ [Mn(L(O_2_))(Cl_2_AcO)]^−^		5.0	
[Mn(L(NH))(O_2_)]^+^ + [Cl_2_AcO]^−^ ⇌ [Mn(L(NH))(Cl_2_AcO)] + O_2_		–7.7	

Further analysis suggests that ligand protonation results in a greater activation energy for O_2_ binding. The Δ*G* ^‡^ for O_2_ binding to [Mn­(L­(NH))]^+^ is 48.8 kcal/mol (Δ*G* = 9.0 kcal/mol), while the Δ*G* ^‡^ for O_2_ to [Mn­(L)]^0^ is 14.5 kcal/mol (Δ*G* = 2.5 kcal/mol). While this suggests the loss of catalytic activity for the ORR upon ligand protonation is due in part to an increased kinetic barrier, geometry calculations indicate protonation induces minimal structural changes to the catalyst. Changes in the predicted charge and spin density on Mn are minimal between [Mn­(L)]^0^ and [Mn­(L­(NH))]^+^, and their respective *d* orbital ordering remains consistent between the formally Mn­(II) complexes and their corresponding dioxygen adducts. Although the Mn to O_2_ distance in the transition state for O_2_ binding to [Mn­(L)]^0^ (2.75 Å) is significantly longer than that found for [Mn­(L­(NH))]^+^ (2.34 Å), the extent of O O bond activation is almost identical: 1.22 and 1.24 Å, respectively. Thus, the primary change seems to be a global stabilization of *d* orbital energies, rendering them less reactive.

However, in addition to this inhibition, it should be noted that the bleaching observed in UV–vis-SEC experiments suggests that decomposition is likely to occur under these conditions, *vide supra*, which would also easily explain the observed inhibition of the catalytic response. Superoxide protonation was found to be endergonic, however, a proton-coupled electron transfer (PCET) to generate a Mn­(III)-hydroperoxide is favorable for both Cl_2_AcOH and ClAcOH, with calculated *E* ^0^ vs Fc^+/0^ of 0.55 and 0.32 V, respectively (Table 2). This hydroperoxo intermediate is presumed to be the primary branching point between the higher efficiency for water observed under electrochemical conditions and the absolute selectivity for hydrogen peroxide observed in spectrochemical experiments. Based on the experimentally observed binding of acetate derivatives to the Mn center of **1Cl**, the influence of acetate binding on O_2_ adducts was also examined computationally. The coordination of the conjugate base dichloroacetate [Cl_2_AcO]^−^ to Mn­(L)­(O_2_) was found to be endergonic by 5.0 kcal/mol. The same reaction between [Mn­(L­(NH))­(O_2_)]^+^ and [Cl_2_AcO]^−^ was thermodynamically favorable with a ΔG = −7.7 kcal/mol but induced O_2_ dissociation. Thus, acetate coordination is unfavorable if the ligand is unprotonated and inhibitory if the ligand is protonated, contrasting with observations made by Mayer and co-workers with charged Fe porphyrin derivatives.^53^

## Discussion

Based on these mechanistic and computational results, it is possible to propose a catalytic cycle for the ORR mediated by **1Cl** (Scheme 1). Starting from (**i**), a single-electron reduction allows O_2_ binding paired with chloride anion loss to generate a Mn–superoxide intermediate (**ii**). From here, two pathways are possible: (**ii**) can undergo dimerization to form an off-cycle, peroxo-bridged species (**ii-D**) or subsequent reduction and protonation results in the formation of a Mn–hydroperoxo species, (**iii**). The mechanism then diverges at this point based on the nature of the reducing agent. With the solubilized chemical reductant Cp*_2_Fe, the proximal oxygen of the Mn–hydroperoxo is preferentially protonated to release H_2_O_2_ and reform the precatalyst, (**i**). As noted above, all spectrochemical experiments showed exclusive selectivity for H_2_O_2_, regardless of the acid source. Based on the acid-independent, quantitative selectivity observed in spectrochemical experiments, it is thought that Cp*_2_Fe is unable to reduce the Mn-hydroperoxo species further. Under electrochemical conditions, however, the observed increased production of H_2_O implies that the reduction of the Mn–hydroperoxo species to induce distal protonation and form an Mn=O species (**iv**) is possible. Presumably, the reduction potential of this species is more negative than that which initiates catalysis and is therefore only accessible during potentiostatic experiments, where the voltage sweeps more negative than the standard potential of Cp*_2_Fe. From there, subsequent reduction and protonation of (**iv**) can presumably generate an intermediate Mn–OH (**v**) species that can be protonated to generate H_2_O and eventually reform the precatalyst (**i**). Although dimerization could also favor O–O bond cleavage en route to water generation, the electrochemical response shows a catalytic current dependence on [Mn] which is less than one, suggesting that this pathway is significantly slower. With TFAH and high concentrations of Cl_2_AcOH, protonation of (**i**) can occur and inhibit catalysis.

**Scheme 1:**
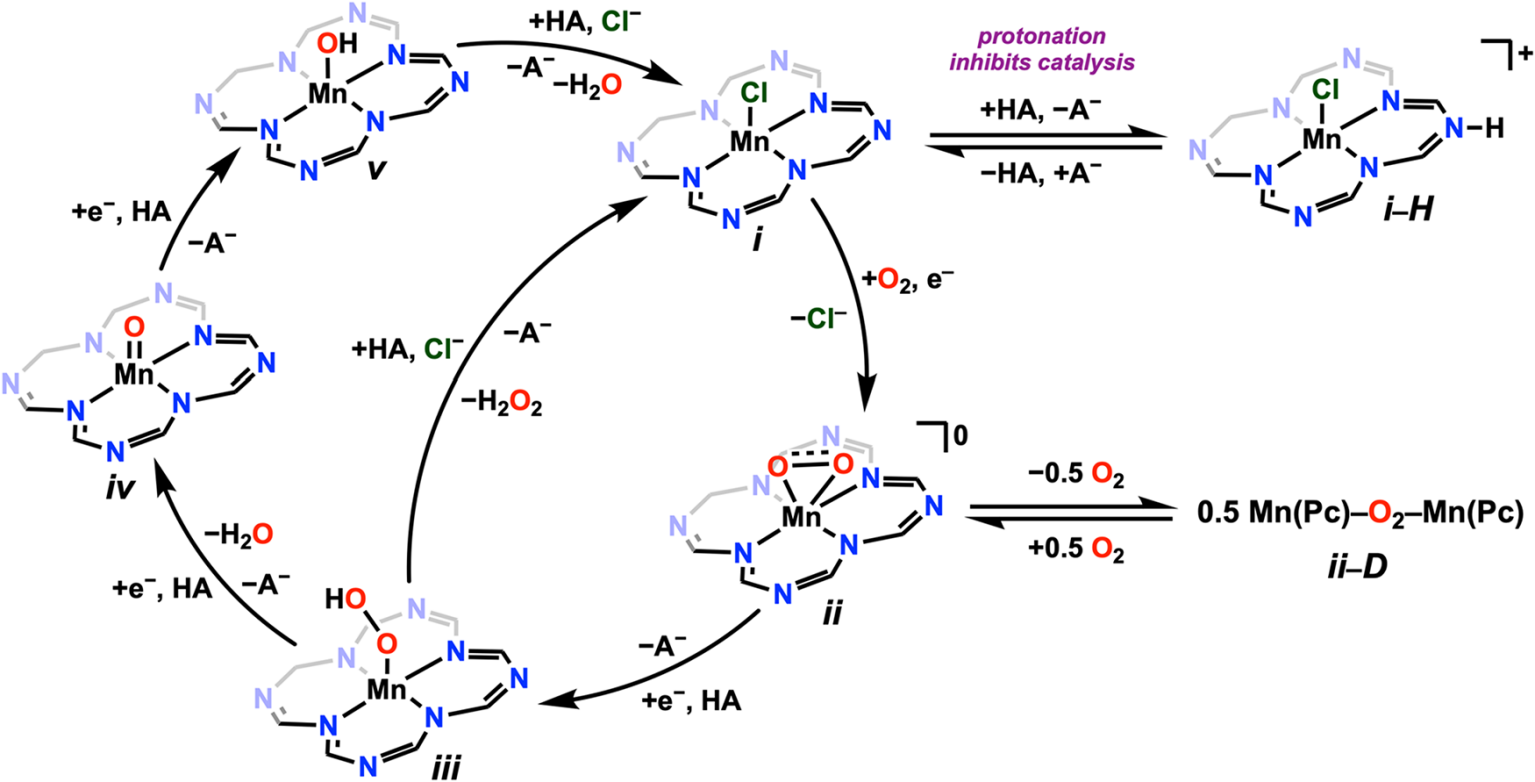
Proposed Mechanism for ORR Catalyzed by **1Cl** ^a^

a Structure truncated to the inner macrocycle of the phthalocyanine ligand for clarity.

Under spectrochemical conditions, the observed catalytic performance with ClAcOH or the Cl_2_AcOH:NaCl_2_AcO buffer exhibits a first-order concentration dependence on **1Cl** and a first-order dependence on O_2_. Based on this experimental evidence and the Eyring analysis for buffered conditions (Figure S59), O_2_ binding to a resting state Mn­(II) species is assigned to the rate-determining step of the reaction. However, unbuffered Cl_2_AcOH does not show a concentration dependence on O_2_ in its catalytic activity; instead, a first-order dependence on **1Cl** and an inverse dependence on acid is observed. This suggests that escape from the protonation equilibrium connecting **i** and **i-H** becomes rate-determining under more strongly acidic conditions. Thus, the extent of ligand protonation (and therefore catalyst deactivation) is relevant to the observed rate of ORR under these conditions. The inclusion of the conjugate base attenuates the proton activity in solution, pushing it toward a less favorable ligand protonation equilibrium and providing an increased concentration of the more active unprotonated form of the catalyst.

ClAcOH, Cl_2_AcOH, and the buffered system all display a half-order concentration dependence on **1Cl**, a first-order dependence on O_2_, and a dependence on acid for catalytic activity under electrochemical conditions. A half-order dependence on catalyst concentration for catalytic activity is consistent with the formation of a dimeric species that is not on the primary reaction pathway, which is ascribed to the formation of a bridging peroxo species as described above. While ClAcOH displays a positive, first-order activity dependence on acid concentration, both Cl_2_AcOH and the buffered system show an inverse dependence because of the ability of Cl_2_AcOH to protonate and deactivate the catalyst. The observed difference in selectivity between ClAcOH and the buffered conditions during electrochemical experiments suggests that the stronger acid is more adept at protonating the distal oxygen than the proximal oxygen under potentiostatic conditions where more reducing potentials are reached.

## Conclusion

Herein, Mn­(^tBu^phthalocyanine)­Cl is reported as a catalyst for the homogeneous ORR under electrochemical and spectrochemical conditions. Notably, the activity and selectivity of the catalytic ORR response are controlled by acid strength, since ligand protonation is found to deactivate the catalyst. In the presence of two acetic acid derivatives, Cl_2_AcOH and ClAcOH (p*K*_a_ in MeCN of 17.3 and 20.3, respectively), the formation of a peroxo-bridged dimer is implied by electroanalytical techniques. However, overall, the selectivity of the ORR depends on the strength of the reducing agent, with solubilized Cp*_2_Fe resulting in quantitative H_2_O_2_ production and potentiostatic electrochemical conditions resulting in the formation of H_2_O. The TOFs observed for spectrochemical conditions are significantly faster than electrochemical conditions and the observed concentration dependences on Mn are consistent with a decreased contribution of dimerization to the reaction rate. The decreased role for dimerization is expected for the spectrochemical experiments, which use a much lower catalyst concentration than the electrochemical conditions. DFT methods show that the effect of ligand protonation is primarily to overly stabilize the *d* orbitals of the Mn center, rendering the reduced Mn­(II) complex less reactive. Thus, the results imply that synthetic strategies to kinetically protect or lower the basicity of the azomethine groups will have implications for proton-dependent electrocatalytic transformations based on metallophthalocyanines.

## Supplementary Material





## Notes

Description of experimental details and methods, electrochemical data, UV–vis, and microscopy data (PDF)
computational coordinates (xyz)

## References

[1] Contribution of Working Groups I, I., and III to the Sixth Assessment Report of the Intergovernmental Panel on Climate Change IPCC; Climate Change 2023: Synthesis Report; IPCC: Geneva, Switzerland; 2023.

[2] Machan C. W. (2020). Advances
in the Molecular Catalysis of Dioxygen Reduction. ACS Catal..

[3] Pegis M. L., Wise C. F., Martin D. J., Mayer J. M. (2018). Oxygen Reduction
by Homogeneous Molecular Catalysts and Electrocatalysts. Chem. Rev..

[4] Cook E. N., Machan C. W. (2022). Homogeneous catalysis of dioxygen
reduction by molecular
Mn complexes. Chem. Commun..

[5] Li Y., Wang N., Lei H., Li X., Zheng H., Wang H., Zhang W., Cao R. (2021). Bioinspired
N4-metallomacrocycles
for electrocatalytic oxygen reduction reaction. Coord. Chem. Rev..

[6] Zagal J. H., Griveau S., Silva J. F., Nyokong T., Bedioui F. (2010). Metallophthalocyanine-based
Molecular Materials as Catalysts for Electrochemical Reactions. Coord. Chem. Rev..

[7] Honda T., Kojima T., Fukuzumi S. (2012). Proton-Coupled Electron-Transfer
Reduction of Dioxygen Catalyzed by a Saddle-Distorted Cobalt Phthalocyanine. J. Am. Chem. Soc..

[8] Ogunsipe A.
O., Idowub M. A., Ogunbayoc T. B., Akinbulu I. A. (2012). Protonation of some
non-transition metal phthalocyanines — spectral and photophysicochemical
consequences. J. Porphyrins Phthalocyanines.

[9] Chen Z., Jiang S., Kang G., Nguyen D., Schatz G. C., Duyne R. P. V. (2019). Operando Characterization
of Iron Phthalocyanine Deactivation
during Oxygen Reduction Reaction Using Electrochemical TipEnhanced
Raman Spectroscopy. J. Am. Chem. Soc..

[10] Eisner U., Harding M. J. C. (1964). 788. Metalloporphyrins. Part I. Some
novel demetallation
reactions. J. Chem. Soc..

[11] Yamada Y., Yoshida S., Honda T., Fukuzumi S. (2011). Protonated iron–phthalocyanine
complex used for cathode material of a hydrogen peroxide fuel cell
operated under acidic conditions. Energy Environ.
Sci..

[12] Phung Q. M., Pierloot K. (2018). The dioxygen adducts of iron and manganese porphyrins:
electronic structure and binding energy. Phys.
Chem. Chem. Phys..

[13] VanAtta R. B., Strouse C. E., Hanson L. K., Valentine J. S. (1987). [Peroxotetraphenylporphinato]­manganese­(III)
and [Chlorotetraphenylporphinato]­manganese­(II) Anions. Syntheses,
Crystal Structures, and Electronic Structures. J. Am. Chem. Soc..

[14] Passard G., Dogutan D. K., Qiu M., Costentin C., Nocera D. G. (2018). Oxygen Reduction Reaction Promoted by Manganese Porphyrins. ACS Catal..

[15] Fukuzumi S., Mochizuki S., Tanaka T. (1989). Metalloporphyrin-Catalyzed Reduction
of Dioxygen by Ferrocene Derivatives. Chem.
Lett..

[16] Fukuzumi S., Mochizuki S., Tanakalb T. (1989). Efficient Reduction of Dioxygen with
Ferrocene Derivatives, Catalyzed by Metalloporphyrins in the Presence
of Perchloric Acid. Inorg. Chem..

[17] Lieske L. E., Hooe S. L., Nichols A. W., Machan C. W. (2019). Electrocatalytic
reduction of dioxygen by Mn­(iii) meso-tetra­(N-methylpyridinium-4-yl)­porphyrin
in universal buffer. Dalton Trans..

[18] Yamamoto, S. ; Dudkin, S. V. ; Kimura, M. ; Kobayashi, N. Phthalocyanine Synthesis. In Handbook of Porphyrin Synthesis; World Scientific, 2019; Vol. 45, pp 1–168.

[19] Metz J., Schneider O., Hanack M. (1984). Synthesis and Properties of Substituted
(Phthalocyaninato)iron and -cobalt Compounds and Their Pyridine Adducts. Inorg. Chem..

[20] Cook E. N., Flaxman L. A., Reid A. G., Dickie D. A., Machan C. W. (2024). Acid Strength
Effects on Dimerization during Metal-Free Catalytic Dioxygen Reduction. J. Am. Chem. Soc..

[21] Kaljurand I., Kütt A., Sooväli L., Rodima T., Mäemets V., Leito I., Koppel I. A. (2005). Extension of the Self-Consistent
Spectrophotometric Basicity Scale in Acetonitrile to a Full Span of
28 pKa Units: Unification of Different Basicity Scales. J. Org. Chem..

[22] McCarthy B. D., Martin D. J., Rountree E. S., Ullman A. C., Dempsey J. L. (2014). Electrochemical
Reduction of Brønsted Acids by Glassy Carbon in Acetonitrile-Implications
for Electrocatalytic Hydrogen Evolution. Inorg.
Chem..

[23] Dean W. S., Soucy T. L., Rivera-Cruz K. E., Filien L. L., Terry B. D., McCrory C. C. L. (2025). Mitigating Cobalt
Phthalocyanine Aggregation in Electrocatalyst
Films through Codeposition with an Axially Coordinating Polymer. Small.

[24] McKinnon, M. ; Rochford, J. Principles of Electrocatalysis. In Green Chemistry; Török, B. ; Dransfield, T. , Eds.; Elsevier, 2018; pp 695–727.

[25] Savéant, J.-M. ; Costentin, C. Elements of Molecular and Biomolecular Electrochemistry: An Electrochemical Approach to Electron Transfer Chemistry; John Wiley & Sons, 2019.

[26] Faulkner, L. R. ; Bard, A. J. Electrochemical Methods: Fundamentals and Applications; John Wiley & Sons, Inc, 2001.

[27] Nichols A. W., Cook E. N., Gan Y. J., Miedaner P. R., Dressel J. M., Dickie D. A., Shafaat H. S., Machan C. W. (2021). Pendent Relay Enhances
H2O2 Selectivity during Dioxygen Reduction Mediated by Bipyridine-Based
Co–N2O2 Complexes. J. Am. Chem. Soc..

[28] Cook E. N., Courter I. M., Dickie D. A., Machan C. W. (2024). Controlling
product
selectivity during dioxygen reduction with Mn complexes using pendent
proton donor relays and added base. Chem. Sci..

[29] Hooe S. L., Machan C. W. (2019). Dioxygen Reduction
to Hydrogen Peroxide by a Molecular
Mn Complex: Mechanistic Divergence between Homogeneous and Heterogeneous
Reductants. J. Am. Chem. Soc..

[30] Frisch, M. J. ; Trucks, G. W. ; Schlegel, H. B. ; Scuseria, G. E. ; Robb, M. A. ; Cheeseman, J. R. ; Scalmani, G. ; Barone, V. ; Petersson, G. A. ; Nakatsuji, H. ; Li, X. ; Caricato, M. ; Marenich, A. V. ; Bloino, J. ; Janesko, B. G. ; Gomperts, R. ; Mennucci, B. ; Hratchian, H. P. ; Ortiz, J. V. ; Izmaylov, A. F. ; Sonnenberg, J. L. ; Williams-Young, D. ; Ding, F. ; Lipparini, F. ; Egidi, F. ; Goings, J. ; Peng, B. ; Petrone, A. ; Henderson, T. ; Ranasinghe, D. ; Zakrzewski, V. G. ; Gao, J. ; Rega, N. ; Zheng, G. ; Liang, W. ; Hada, M. ; Ehara, M. ; Toyota, K. ; Fukuda, R. ; Hasegawa, J. ; Ishida, M. ; Nakajima, T. ; Honda, Y. ; Kitao, O. ; Nakai, H. ; Vreven, T. ; Throssell, K. ; Montgomery Jr, J. A. ; Peralta, J. E. ; Ogliaro, F. ; Bearpark, M. J. ; Heyd, J. J. ; Brothers, E. N. ; Kudin, K. N. ; Staroverov, V. N. ; Keith, T. A. ; Kobayashi, R. ; Normand, J. ; Raghavachari, K. ; Rendell, A. P. ; Burant, J. C. ; Iyengar, S. S. ; Tomasi, J. ; Cossi, M. ; Millam, J. M. ; Klene, M. ; Adamo, C. ; Cammi, R. ; Ochterski, J. W. ; Martin, R. L. ; Morokuma, K. ; Farkas, O. ; Foresman, J. B. ; Fox, D. J. Gaussian 16 Rev. B.01; GaussView 5.0; Gaussian, Inc.: Wallingford CT, 2016.

[31] Becke A. D. (1993). Density-functional
thermochemistry. III. The role of exact exchange. J. Chem. Phys..

[32] Lee C., Yang W., Parr R. G. (1988). Development of the Colle-Salvetti
correlation-energy formula into a functional of the electron density. Phys. Rev. B.

[33] Vosko S. H., Wilk L., Nusair M. (1980). Accurate spin-dependent electron
liquid correlation energies for local spin density calculations: a
critical analysis. Can. J. Phys..

[34] Stephens P.
J., Devlin F. J., Chabalowski C. F., Frisch M. J. (1994). Ab Initio Calculation
of Vibrational Absorption and Circular Dichroism Spectra Using Density
Functional Force Fields. J. Phys. Chem. A.

[35] Weigend F. (2006). Accurate Coulomb-fitting
basis sets for H to Rn. Phys. Chem. Chem. Phys..

[36] Weigend F., Ahlrichs R. (2005). Balanced basis sets
of split valence, triple zeta valence
and quadruple zeta valence quality for H to Rn: Design and assessment
of accuracy. Phys. Chem. Chem. Phys..

[37] Grimme S., Antony J., Ehrlich S., Krieg H. (2010). A consistent and accurate
ab initio parametrization of density functional dispersion correction
(DFT-D) for the 94 elements H-Pu. J. Chem. Phys..

[38] Grimme S., Ehrlich S., Goerigk L. (2011). Effect of
the damping function in
dispersion corrected density functional theory. J. Comput. Chem..

[39] Izsák R., Hansen A., Neese F. (2012). The resolution
of identity and chain
of spheres approximations for the LPNO-CCSD singles Fock term. Mol. Phys..

[40] Izsák R., Neese F. (2011). An overlap fitted chain of spheres
exchange method. J. Chem. Phys..

[41] Izsák R., Neese F., Klopper W. (2013). Robust fitting techniques in the
chain of spheres approximation to the Fock exchange: The role of the
complementary space. J. Chem. Phys..

[42] Neese F. (2012). The ORCA program
system. WIREs Comput. Mol. Sci..

[43] Neese F. (2018). Software update:
the ORCA program system, version 4.0. WIREs
Comput. Mol. Sci..

[44] Neese F. (2022). Software update:
The ORCA program system—Version 5.0. WIREs Comput. Mol. Sci..

[45] Neese F., Wennmohs F., Becker U., Riplinger C. (2020). The ORCA quantum
chemistry program package. J. Chem. Phys..

[46] Rappoport D. (2021). Property-optimized
Gaussian basis sets for lanthanides. J. Chem.
Phys..

[47] Rappoport D., Furche F. (2010). Property-optimized
Gaussian basis sets for molecular
response calculations. J. Chem. Phys..

[48] Caldeweyher E., Bannwarth C., Grimme S. (2017). Extension of the D3 dispersion coefficient
model. J. Chem. Phys..

[49] Caldeweyher E., Ehlert S., Hansen A., Neugebauer H., Spicher S., Bannwarth C., Grimme S. (2019). A generally applicable
atomic-charge dependent London dispersion correction. J. Chem. Phys..

[50] Caldeweyher E., Mewes J.-M., Ehlerta S., Grimme S. (2020). Extension and evaluation
of the D4 London-dispersion model for periodic systems. Phys. Chem. Chem. Phys..

[51] Najibi A., Goerigk L. (2020). DFT-D4 counterparts of leading meta-generalized-gradient
approximation and hybrid density functionals for energetics and geometries. J. Comput. Chem..

[52] Fourmond V., Jacques P.-A., Fontecave M., Artero V. (2010). H_2_ Evolution
and Molecular Electrocatalysts: Determination of Overpotentials and
Effect of Homoconjugation. Inorg. Chem..

[53] Martin D. J., Mercado B. Q., Mayer J. M. (2020). Combining
scaling relationships overcomes
rate versus overpotential trade-offs in O2 molecular electrocatalysis. Sci. Adv..

