# Supplementary material for: Ring Protonation Regulates the Homogeneous Electrocatalytic Oxygen Reduction Reaction Mediated by Manganese Phthalocyanine
Source: J Am Chem Soc. 2025 Nov 21;147(49):45221–9. doi: 10.1021/jacs.5c14858 (PMC12703747; doi:10.1021/jacs.5c14858)
Supplement: Supplementary file 1 [file ja5c14858_si_001.pdf]

Supporting Information

Ring Protonation Regulates the Homogeneous Electrocatalytic Oxygen Reduction Reaction Mediated by Manganese Phthalocyanine

Mary Jo McCormick, Kate G. Scheerer and Charles W. Machan*

* - machan@virginia.edu; ORCID 0000-0002-5182-1138

MJM ORCID 0000-0001-6657-411X, KGS ORCID 0009-0003-9072-7587

Department of Chemistry, University of Virginia,

PO Box 400319, Charlottesville, VA 22904-4319, USA

Table of Contents

Experimental Methods.....7

General Considerations.....7

Synthesis and Characterization.....7

Synthesis of [Mn(^{tBu}phthalocyanine)Cl].....7

Figure S1. (A) UV-vis absorbance data for serial dilutions of 1Cl in MeCN 7

Spectrochemical Analysis.....8

Spectrochemical Analysis of Ligand Protonation 8

Figure S2. (A) Uv-vis spectra of **1Cl** in MeCN with increasing amounts of 2,6-lutidinium tetrafluoroborate 9

Figure S3. UV-vis spectra of **1Cl** (25 μM) in MeCN with increasing amounts of Cl_2AcOH 9

Figure S4. UV-vis spectra of **1Cl** (25 μM) in MeCN with increasing amounts of ClAcOH, ...10

Figure S5. (A) UV-vis spectra of **1Cl** (25 μM) in MeCN with increasing amounts of 1:1 $Cl_2AcOH:NaCl_2AcO$ buffer 10

Figure S6. (A) UV-vis spectra of **1Cl** (10 μM) in MeCN with increasing amounts of sodium tetrakis[3,5-bis(trifluoromethyl)phenyl]borate (NaBArF).....11

Figure S7. UV-vis spectra of **1Cl** (black trace, 25 μM) in MeCN with 5 mM sodium trifluoroacetate.....11

Figure S8. UV-vis spectra of **1Cl** (black trace, 25 μM) in MeCN with 5 mM tetrabutylammonium acetate.....12

UV-Vis Spectroelectrochemical Analysis.....12

Description of Au Electrode Cleaning Procedure.....12

Description of General Procedure 12

Figure S9. UV-vis showing spectral changes of **1Cl** with TFAH under (A) inert atmosphere and (B) ambient air with applied potentials.....13

Figure S10. Comparison of the initial spectra with the spectra of the fully reduced **1Cl** species with TFAH 13

Figure S11. UV-vis showing spectral changes of **1Cl** with 20 eq. Cl_2AcOH under (A) inert atmosphere and (B) ambient air with applied potentials 14

Figure S12. UV-vis showing spectral changes of **1Cl** with 100 eq. Cl_2AcOH under (A) inert atmosphere and (B) ambient air with applied potentials 14

Figure S13. UV-vis showing spectral changes of **1Cl** with 60 eq. Cl_2AcOH/Cl_2AcO^- buffer under (A) inert atmosphere and (B) ambient air with applied potentials 15

Figure S14. Comparison of the initial spectra with the spectra of the fully reduced **1Cl** species with Cl_2AcOH buffer 15

Figure S15. UV-vis showing spectral changes of **1Cl** with ClAcOH under (A) inert atmosphere and (B) ambient air with applied potentials 16

Figure S16. Comparison of the initial spectra with the spectra of the fully reduced **1Cl** species with ClAcOH 16

Table S1. Summary of λ_{max} wavelengths (nm) associated with peaks in UV-vis-SEC spectra 17

Electrochemical Analysis of 1Cl 17

Electrochemistry 17

Rotating Ring-Disk Voltammetry Methods 17

Description of Au Ring Roughening Procedure 17

Description of RRDE Collection Efficiency 18

Description of RRDE Experiment 18

Figure S17. (A) CVs of **1Cl** under inert atmosphere at variable scan rate and (B) plot showing the linear dependence of current density on the square root of scan rate 19

Figure S18. (A) CVs of **1Cl** under Oxygen-saturation at variable scan rate and (B) plot showing the linear dependence of peak voltage on the log of scan rate 20

Figure S19. (A) CVs of **1Cl** variable O_2 concentrations. (B) Log-Log plot of Oxygen concentration versus the current density 20

Figure S20. CVs of **1Cl** under Ar (black), O_2 (red) and with 50 mM TFAH under Ar (green) and oxygen-saturated (blue) conditions 21

Electrochemical Studies with Cl_2AcOH 21

Figure S21. (A) CVs of **1Cl** under catalytic conditions with variable Cl_2AcOH concentrations. (B) Log-Log plot of the log of the acid concentration versus the catalytic current density 21

Figure S22. (A) CVs of **1Cl** under catalytic conditions with variable O_2 concentrations. (B) Log-Log plot of Oxygen concentration versus the catalytic current density 22

Figure S23. CVs of **1Cl** under inert (A) and (B) catalytic conditions with variable **1Cl** concentrations 22

Figure S24. Log-Log plot of **1Cl** concentration versus the catalytic current density 23

Figure S25. (A) CVs of **1Cl** under catalytic conditions with variable O_2 concentrations. (B) Log-Log plot of Oxygen concentration versus the catalytic current density 23

Figure S26. CVs of **1Cl** under inert (A) and (B) catalytic conditions with variable Mn(^{tBu}phthalocyanine)Cl concentrations 24

Figure S27. Log-Log plot of **1Cl** concentration versus the catalytic current density 24

Electrochemical Studies with Cl_2AcOH/Cl_2AcO^- buffer 25

Figure S31. (A) CVs of **1Cl** under catalytic conditions with variable $NaCl_2AcO$ concentrations. (B) Log-Log plot of the log of the $NaCl_2AcO$ concentration versus the catalytic current density 26

Figure S32. (A) CVs of **1Cl** under inert conditions with $NaPF_6$ (red trace) and Cl_2AcOH (blue trace). (B) CVs of **1Cl** under catalytic conditions with NaPF6 (red trace), Cl_2AcOH (blue trace), and buffer (green trace). 27

Figure S33. CVs of **1Cl** under inert (A) and (B) catalytic conditions with variable **1Cl** concentrations 27

Figure S34. Log-Log plot of **1Cl** concentration versus the catalytic current density from Figure **S33.B** 28

Figure S35. Linear sweep voltammograms of the RRDE experiment with 0.5 mM **1Cl** and 30 mM Cl_2AcOH/Cl_2AcO^- under air saturation 28

Figure S36. (A) Levich and (B) Koutecky-Levich plots from data obtained from linear sweep voltammograms of **1Cl** (0.5 mM) by RRDE with 30 mM Cl_2AcOH/Cl_2AcO^- under air saturation at various rotation rates 29

Electrochemical Studies with ClAcOH 29

Figure S38. (A) CVs of **1Cl** under catalytic conditions with variable O_2 concentrations. (B) Log-Log plot of Oxygen concentration versus the catalytic current density 30

Figure S39. CVs of **1Cl** under inert (A) and (B) catalytic conditions with variable **1Cl** concentrations 30

Figure S40. Log-Log plot of **1Cl** concentration versus the catalytic current density from Figure **S39.B** 31

Figure S41. Determination of turnover frequency (TOF). (A) Variable scan rate CVs of **1Cl** with ClAcOH under O_2-saturation. (B) Calculated TOF from each scan rate versus the scan rate 31

Calculation of TOF 31

Figure S42. Linear sweep voltammograms of the RRDE experiment with 0.5 mM **1Cl** and 50 mM ClAcOH under air saturation 32

Figure S43. (A) Levich and (B) Koutecky-Levich plots from data obtained from linear sweep voltammograms of **1Cl** (0.5 mM) by RRDE with 50 mM ClAcOH under air saturation at various rotation rates 32

Stopped-Flow Kinetic Analysis of 1Cl 33

Stopped-Flow with ClAcOH 33

Figure S44. (A) Change in absorbance at 780 nm over time due to the formation of $[Cp^*_2Fe]^+$ by ORR catalyzed by **1Cl** with ClAcOH 33

Calculation of Turnover Frequency 34

Figure S46. Calculated R_{fit}/n_{cat} values from stopped-flow spectrochemical experiments with **1Cl**, O_2, and Cp^*_2Fe with varying ClAcOH concentrations 35

Figure S47. Calculated R_{fit}/n_{cat} values from stopped-flow spectrochemical experiments with ClAcOH, **1Cl**, and Cp^*_2Fe with varying O_2 concentrations 35

Figure S48. Calculated R_{fit}/n_{cat} values from stopped-flow spectrochemical experiments with **1Cl**, ClAcOH, and O_2 with varying Cp^*_2Fe concentrations 36

Stopped-Flow with Cl_2AcOH 36

Figure S49. (A) Change in absorbance at 780 nm over time due to the formation of $[Cp^*_2Fe]^+$ by ORR catalyzed by **1Cl** with Cl_2AcOH (black trace), exampled of a 2Exp + Mx + C fit in Kinetic Studio 4.0 (red trace), and residual fit (blue trace) 36

Figure S50. Calculated R_{fit}/n_{cat} values from stopped-flow spectrochemical experiments with Cl_2AcOH, O_2, and Cp^*_2Fe with varying **1Cl** concentrations 37

Figure S51. Change in absorbance at 780 nm over time due to production of $[Cp^*_2Fe]^+$ by ORR catalyzed by **1Cl** with O_2, Cp^*_2Fe, with varying Cl_2AcOH concentrations 37

Figure S52. Calculated R_{fit}/n_{cat} values from stopped-flow spectrochemical experiments with Cl_2AcOH, **1Cl**, and Cp^*_2Fe with varying O_2 concentrations 38

Figure S53. Calculated R_{fit}/n_{cat} values from stopped-flow spectrochemical experiments with **1Cl**, Cl_2AcOH, and O_2 with varying Cp^*_2Fe concentrations 38

Calculation of Turnover Frequency 39

Stopped-Flow with Cl_2AcOH/Cl_2AcO^- buffer 39

Figure S54. (A) Change in absorbance at 780 nm over time due to the formation of $[Cp^*_2Fe]^+$ by ORR catalyzed by **1Cl** with buffer (black trace), exampled of a 2Exp + Mx + C fit in Kinetic Studio 4.0 (red trace), and residual fit (blue trace) 39

Figure S55. Calculated R_{fit}/n_{cat} values from stopped-flow spectrochemical experiments with buffer, O_2, and Cp^*_2Fe with varying **1Cl** concentrations 40

Calculation of Turnover Frequency 40

Figure S56. Calculated Rfit/ncat values from stopped-flow spectrochemical experiments with **1Cl**, O2, and Cp*2Fe with varying buffer concentrations 41

Figure S57. Calculated R_{fit}/n_{cat} values from stopped-flow spectrochemical experiments with buffer, **1Cl**, and $Cp^*{}_2Fe$ with varying O_2 concentrations..41

Figure S58. Calculated Rfit/ncat values from stopped-flow spectrochemical experiments with **1Cl**, buffer, and O_2 with varying $Cp^*{}_2Fe$ concentrations..42

Eyring Analysis of Buffered Conditions 42

Figure S59. Eyring Plot for variable temperature stopped-flow kinetic data with **1Cl** and the buffer 43

Spectrochemical Analysis...43

ORR Selectivity..43

Preparation of 0.1 M $Ti(O)SO_4$ Solution...44

Preparation of H_2O_2 Calibration Curve 44

Figure S60. Calibration curve of H_2O_2 quantification for $Ti(O)SO_4$ colorimetric assay......44

ORR Product Quantification 44

Table S2. Summary of spectrochemical ORR selectivity by **1Cl** with each acid.................45

Figure S61. H_2O_2 product quantification of ORR by **1Cl** with ClAcOH after 30 s, 2 min, and 5 min 45

Figure S62. H_2O_2 product quantification of ORR by **1Cl** with Cl_2AcOH after 30 s, 2 min, and 5 min 46

Figure S63. H_2O_2 product quantification of ORR by **1Cl** with buffer after 30 s, 1 min, and 2 min...47

H_2O_2 Stability Control Studies 47

Table S3. Summary of H_2O_2 recovery for the stability control studies 48

Figure S64. Stability test of urea•H_2O_2 in the presence of **1Cl**, Cl_2AcOH, and O_2.............48

Figure S65. Stability test of urea•H_2O_2 in the presence of **1Cl**, buffer, and O_2...................49

Figure S66. Stability test of urea•H_2O_2 in the presence of **1Cl**, ClAcOH, and O_2. 49

Computational Methods 50

References 51

Experimental Methods

General Considerations. All chemicals and solvents (ACS or HPLC grade) were commercially available and used as received unless otherwise specified. For all air-sensitive electrochemical and spectrochemical experiments, HPLC-grade solvents were obtained as anhydrous and air-free from a Glass Contour Solvent System from PPT. Gas cylinders were obtained from Praxair (Ar as 5.0 and O_2 as 4.0) and passed through molecular sieves prior to use in electrochemical experiments. Gas mixing for variable concentration experiments was accomplished gas proportioning rotameter from Omega Engineering. UV-vis absorbance spectra were obtained on a Cary 60 from Agilent using a quartz cuvette with a 1 cm pathlength. The concentration of O_2 saturation in MeCN is reported to be 8.1 mM and the saturation concentration with added electrolyte is 6.1 mM.[1]

Synthesis and Characterization

Synthesis of [Mn(^{tBu}phthalocyanine)Cl].[2] In a round-bottom flask, $MnCl_2 \cdot 4H_2O$ (0.50 g, 2.5 mmol) and tert-butylphthalonitrile (1.86 g, 10.1 mmol) were suspended in MeOH (5 mL). The mixture was heated to 250 °C for one hour, during which time the solvent evaporated and a molten flux was achieved. The resulting black solid was cooled to room temperature before being crushed and boiled in 50 mL of HCl (1 N) for one hour. The suspension was filtered, and the dark solid was washed with water (3 x 100 mL). The solid was then crushed and boiled in 50 mL of NaOH (1 N) for one hour before being filtered and washed with water to yield the resulting, dark green solid that was then recrystallized from a mixture of DCM and *n*-pentanes (1.8, 89% yield). Elemental analysis calculated for $C_{48}H_{48}MnN_8Cl \cdot H_2O$: C 68.20, H 5.96, N 13.26; Found: C 68.52, H 5.67, N 13.29. HRMS (ESI) *m/z* calculated for $[C_{48}H_{48}MnN_8]^+$: 791.3382, found 791.3380.

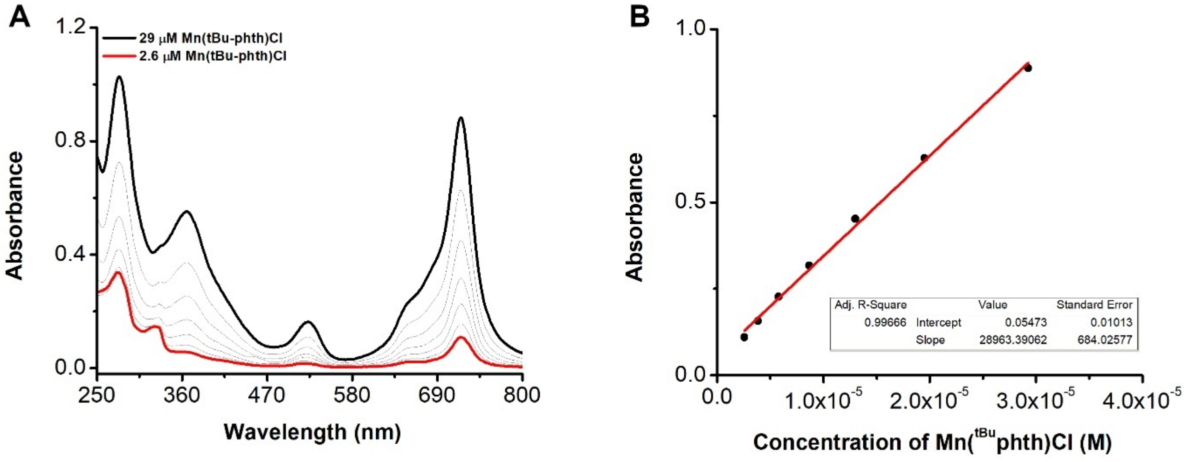

Figure S1. (A) UV-vis absorbance data for serial dilutions of **1Cl** in MeCN. Conditions: varying concentration, quartz cuvette with 1 cm pathlength. (B) Plot absorbance versus concentration (M) for **1Cl** in MeCN solution at 721 nm; $R^2 = 0.997$.

Spectrochemical Analysis

Spectrochemical Analysis of Ligand Protonation

General Procedure. The spectrochemical protonation of **1Cl** was analyzed by UV-vis titration methods. First, separate solutions of 50 µM **1Cl** and of 50 mM acid (2,2,2-trifluoroacetic acid TFAH, dichloroacetic acid Cl_2AcOH, and monochloroacetic acid ClAcOH) in MeCN were made using dry volumetric glassware. The same volume of **1Cl** solution (5 mL) was transferred to five separate volumetric flasks (10 mL) using a gas-tight Hamilton syringe (5000 µL). Then, the acid solution was transferred to each of the volumetric flasks in varying aliquots (5 mL, 4 mL, 3 mL, 2 mL, and 1 mL) using the same gas-tight Hamilton syringe. The flasks were filled with MeCN to achieve a final volume of 10 mL, capped, and inverted to ensure the solution was homogenous throughout before being transferred to the UV-vis cuvette for analysis.

Analysis for Protonation with TFAH and 2,6-lutidinium tetrafluoroborate. As strong acid is titrated into a solution of **1Cl**, the absorbance at 740 nm increases and the absorbance at 711 nm decreases. These absorbance values were used to create a Hill Plot, where the y-axis can be simplified from:

$$y = Log\left[\frac{(A_0 - A_i)}{(A_i - A_0)}\right] \qquad \textbf{(Eq. S1)}$$

to

$$y = Log\left[\frac{Abs_{740}}{Abs_{711}}\right] \qquad \textbf{(Eq. S2)}$$

Where A_0 is the absorbance with the acid present and A_i is the absorbance of **1Cl** without acid.

The protonation equilibrium constant *K* for was calculated from the Hill Plot (**Figure 1C of main text**) and **Figure S2** using **Eq. S3**:

$$pK_{eq} = intercept \qquad \textbf{(Eq. S3)}$$

Thus, the pK_a of **1Cl–H⁺** can be estimated with the following equations, using a value of 12.65 for the pK_a of TFAH[3] and 14.1 for the pK_a of 2,6-lutidinium tetrafluoroborate[4] in MeCN:

$$K_{eq} = 10^{\Delta pKa} \qquad \textbf{(Eq. S4)}$$

$$pK_{eq} = pK_a(\boldsymbol{1Cl - H^+}) - pK_a(HA) \qquad \textbf{(Eq. S5)}$$

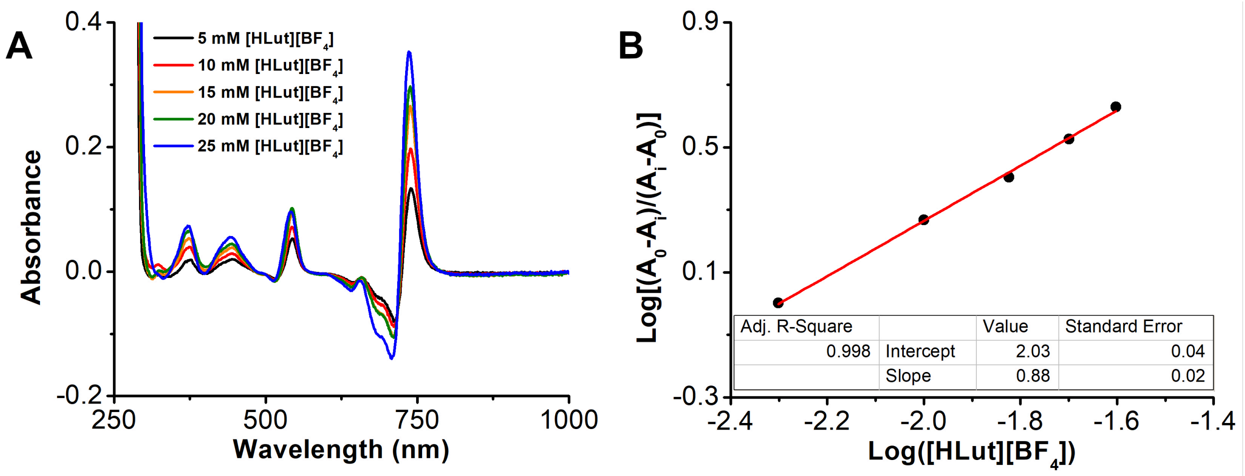

Figure S2. (A) Uv-vis spectra of **1Cl** in MeCN with increasing amounts of 2,6-lutidinium tetrafluoroborate with spectra of **1Cl** (25 μM) subtracted. (B) Hill plot obtained from the absorbance changes at 740 nm in A.

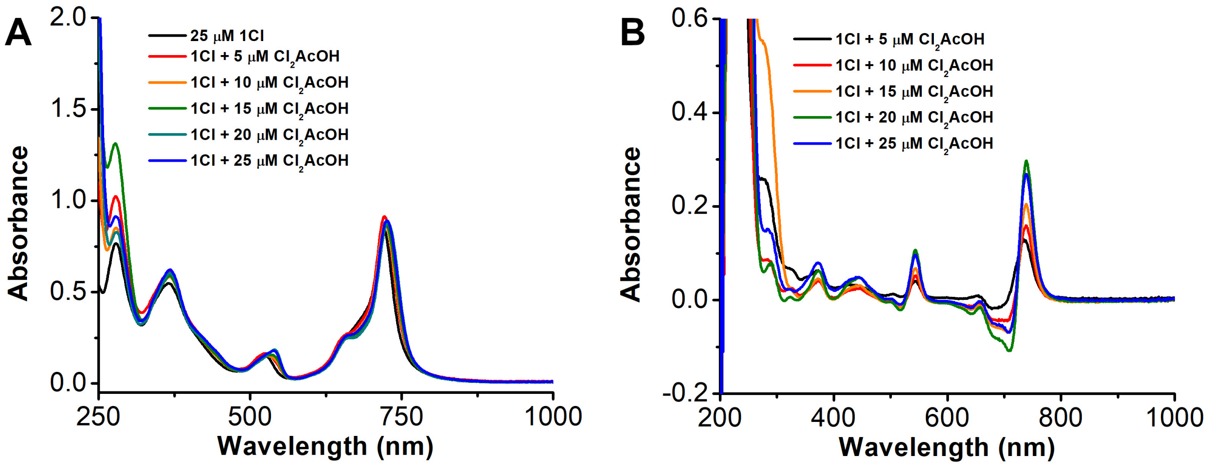

Figure S3. UV-vis spectra of **1Cl** (25 μM) in MeCN with increasing amounts of Cl_2AcOH, and (B) traces from A with unbuffered **1Cl** (black trace) subtracted.

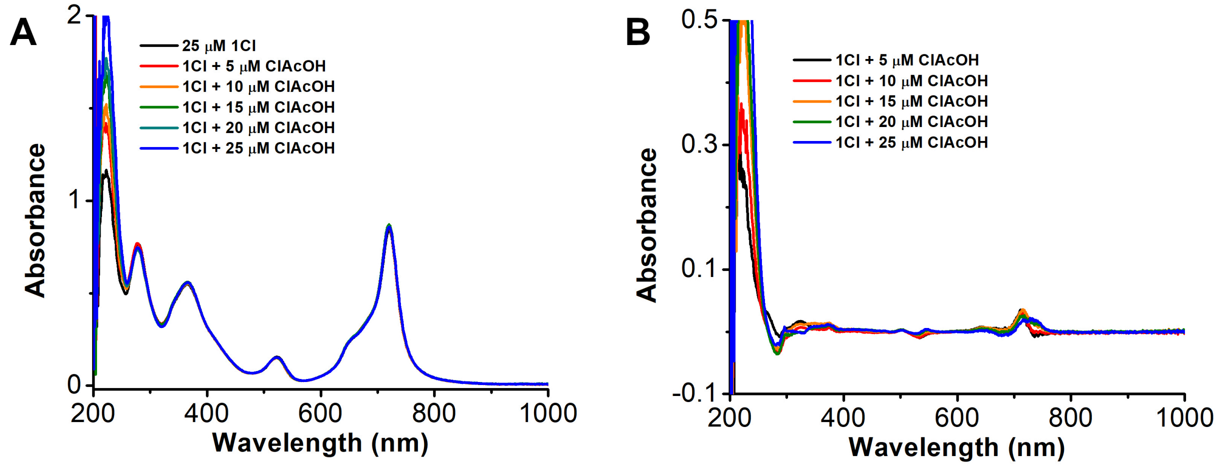

Figure S4. UV-vis spectra of **1Cl** (25 μM) in MeCN with increasing amounts of ClAcOH, and (B) traces from A with unbuffered **1Cl** (black trace) subtracted.

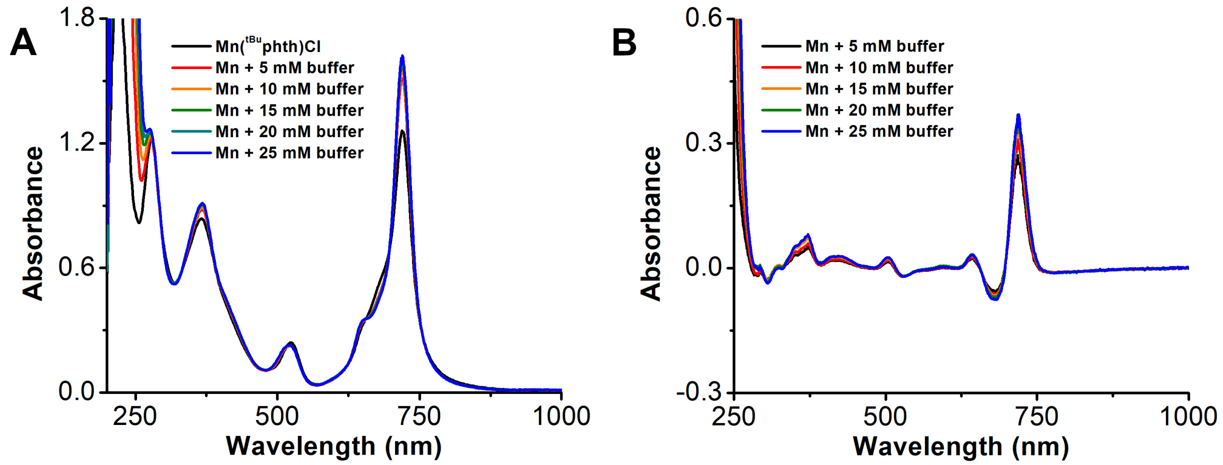

Figure S5. (A) UV-vis spectra of **1Cl** (25 μM) in MeCN with increasing amounts of 1:1 $Cl_2AcOH:NaCl_2AcO$ buffer, and (B) traces from A with unbuffered **1Cl** (black trace) subtracted.

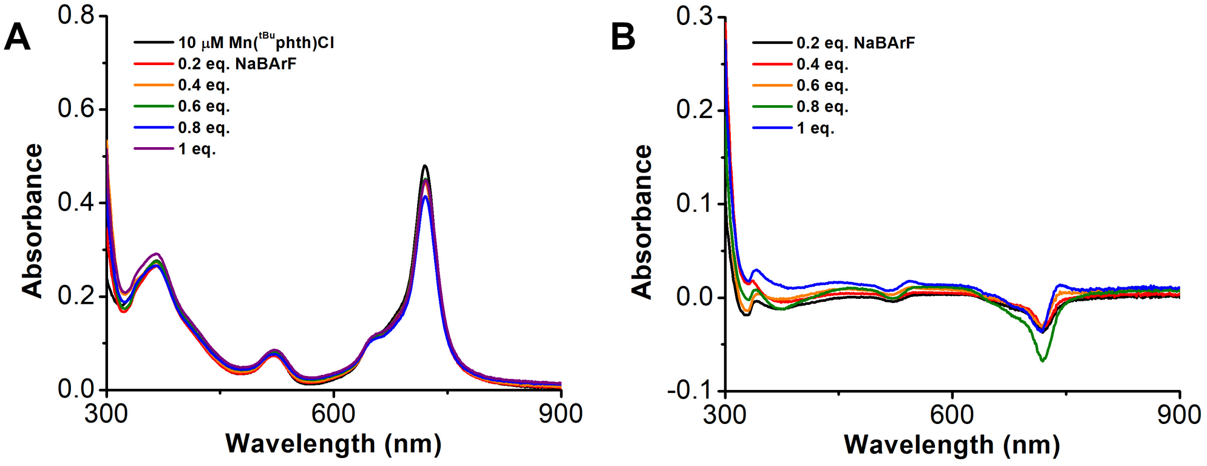

Figure S6. (A) UV-vis spectra of **1Cl** (10 μM) in MeCN with increasing amounts of sodium tetrakis[3,5-bis(trifluoromethyl)phenyl]borate (NaBArF) and (B) traces from A with **1Cl** (black trace) subtracted.

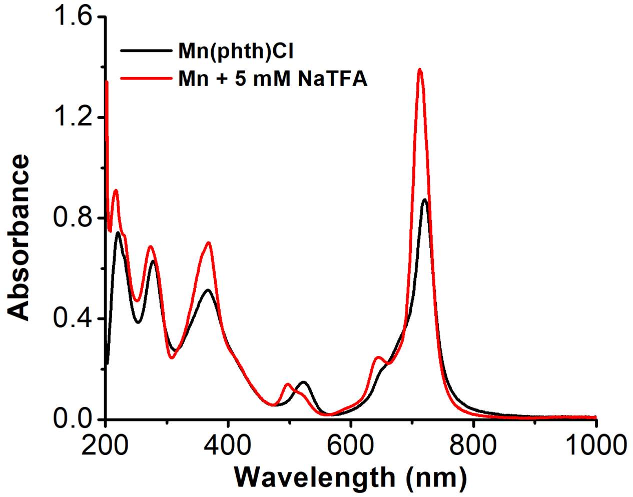

Figure S7. UV-vis spectra of **1Cl** (black trace, 25 μM) in MeCN with 5 mM sodium trifluoroacetate (red trace).

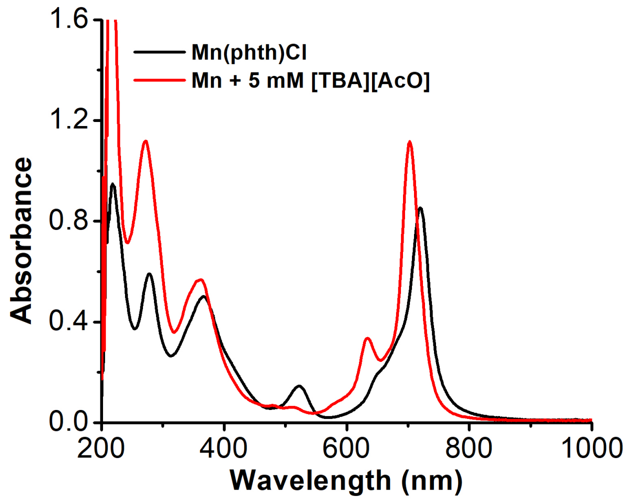

Figure S8. UV-vis spectra of **1Cl** (black trace, 25 μM) in MeCN with 5 mM tetrabutylammonium acetate (red trace).

UV-Vis Spectroelectrochemical Analysis

UV-vis spectroelectrochemistry experiments were performed using an Agilent Cary 60 Spectrometer and a Biologic SP-50 Potentiostat. All experiments were performed with a Pine Honeycomb Spectroelectrochemical Cell and a leakless mini KCl pseudoreference electrode from eDAQ. Tetrabutylammonium hexafluorophosphate ($TBAPF_6$) was purified by recrystallization from ethanol and dried in a vacuum oven before storage in a desiccator. All data were referenced to an external ferrocene standard in which a glassy carbon working (ø = 3 mm) and glassy carbon rod (ø = 3 mM) counter electrode was used. Ferrocene was purified by sublimation prior to use.

Description of Au Electrode Cleaning Procedure. The Au honeycomb electrode was cleaned according to the Pine Spectroelectrochemical Cell User Guide. The electrode was placed in a solution of 0.5 M H_2SO_4, and sequential cyclic voltammetry experiments were performed: Potential range from –0.375 V to 1.8 V, 500 mV/s scan rate, 20 sequential cycles.

Description of General Procedure. A 0.1 M $TBAPF_6$/MeCN stock was used to make all solutions and was used as a blank in the UV-vis spectrometer and was subtracted from all scans. A solution containing **1Cl** was added to the cuvette and loaded onto the working electrode; the cell was equipped with a PTFE sparging line and was sparged with MeCN-saturated argon continuously throughout the experiment. The potentiostat experiment was set to hold each applied potential for 5 minutes; the set potentials began positive of the $Mn^{III/II}$ reduction peak potential and ended more negative. The UV-vis spectrometer was set to 0.20 cycles/minute and scanned from 1000 nm to 200 nm. Once the experiment was completed, the solution was discarded, and the cuvette and electrodes were cleaned thoroughly. This procedure was repeated with fresh **1Cl** solution; however, the argon-sparging line was not placed in the cell. The unused **1Cl** solution was used for sequential acid additions.

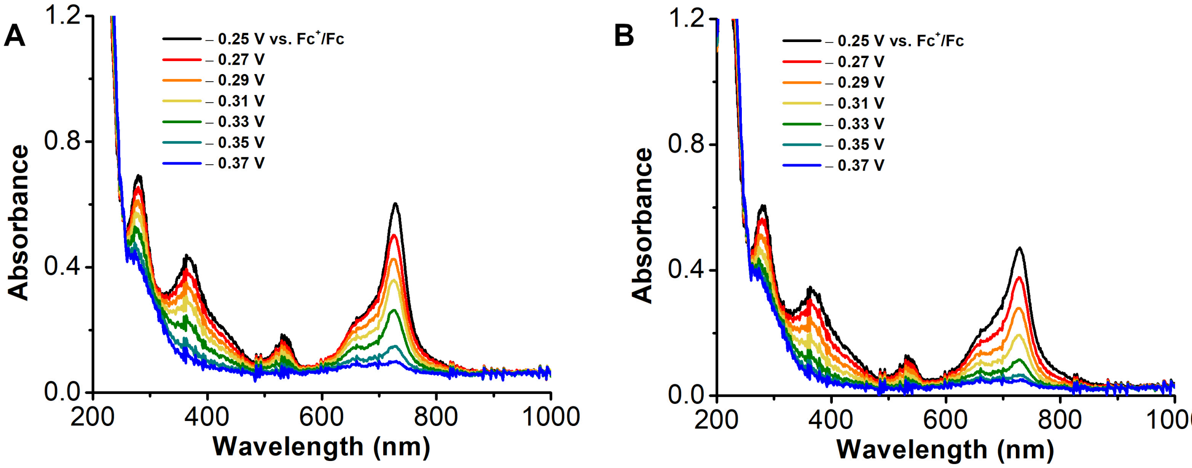

Figure S9. UV-vis showing spectral changes of **1Cl** with TFAH under (A) inert atmosphere and (B) ambient air with applied potentials. Conditions: 86 μM **1Cl** and 86 mM TFAH in 0.1 M $TBAPF_6$/MeCN.

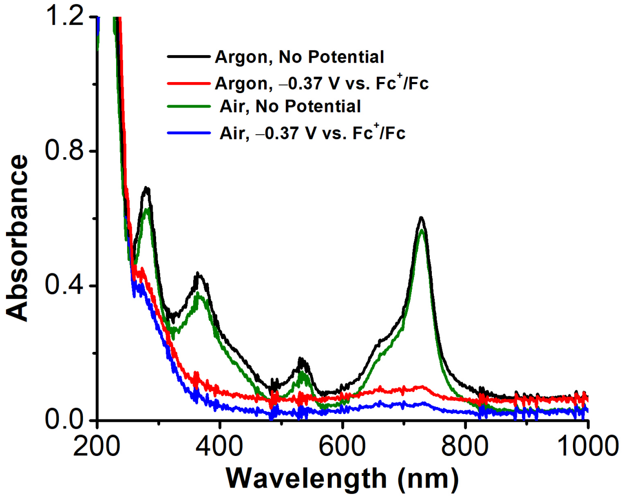

Figure S10. Comparison of the initial spectra with the spectra of the fully reduced **1Cl** species with TFAH. Conditions: 86 μM **1Cl** and 86 mM TFAH in 0.1 M $TBAPF_6$/MeCN.

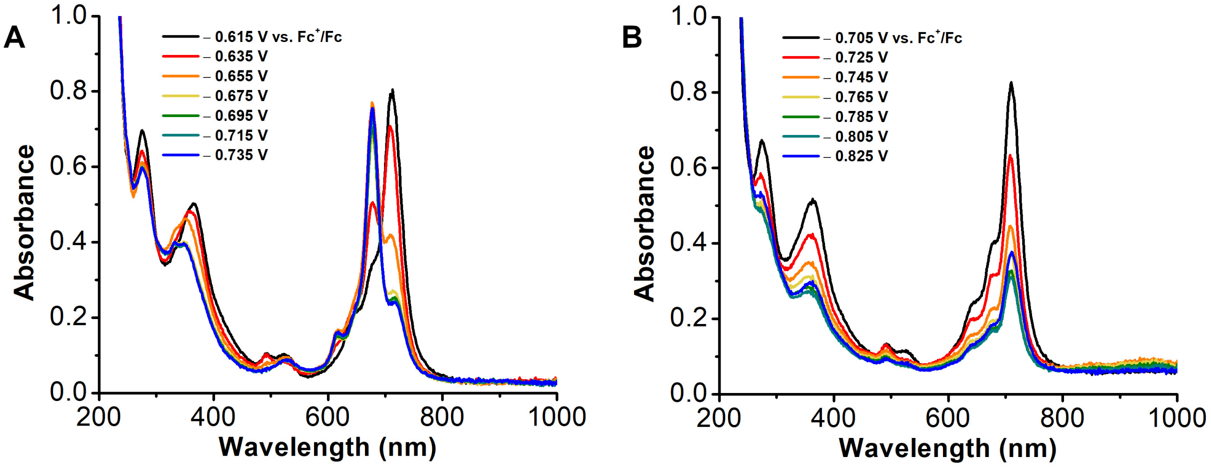

Figure S11. UV-vis showing spectral changes of **1Cl** with 20 eq. Cl_2AcOH under (A) inert atmosphere and (B) ambient air with applied potentials. Conditions: 86 μM **1Cl** and 1.7 mM Cl_2AcOH in 0.1 M $TBAPF_6$/MeCN.

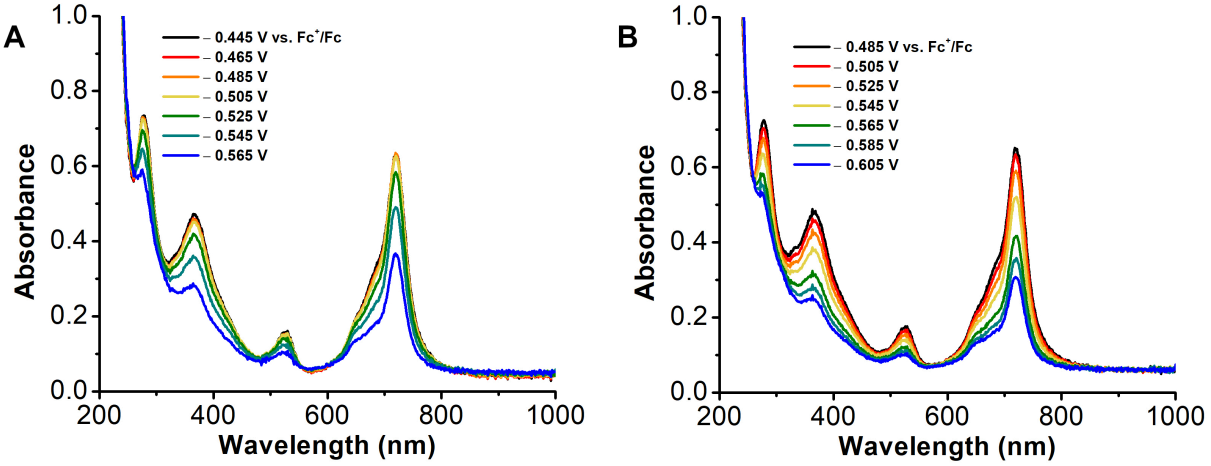

Figure S12. UV-vis showing spectral changes of **1Cl** with 100 eq. Cl_2AcOH under (A) inert atmosphere and (B) ambient air with applied potentials. Conditions: 86 μM **1Cl** and 8.6 mM Cl_2AcOH in 0.1 M $TBAPF_6$/MeCN.

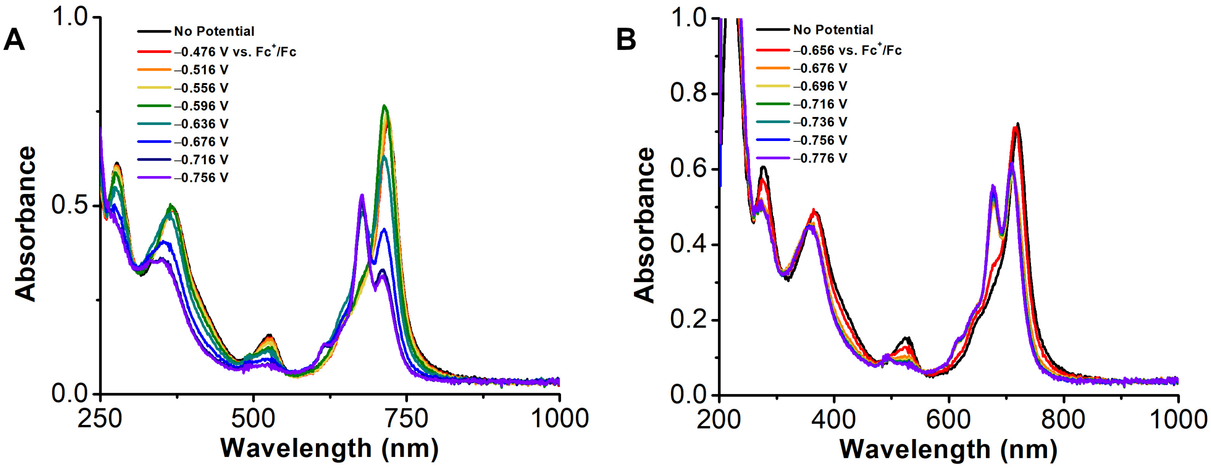

Figure S13. UV-vis showing spectral changes of **1Cl** with 60 eq. Cl_2AcOH/Cl_2AcO^- buffer under (A) inert atmosphere and (B) ambient air with applied potentials. Conditions: 86 μM **1Cl** and 5.2 mM Cl_2AcOH/Cl_2AcO^- in 0.1 M $TBAPF_6$/MeCN.

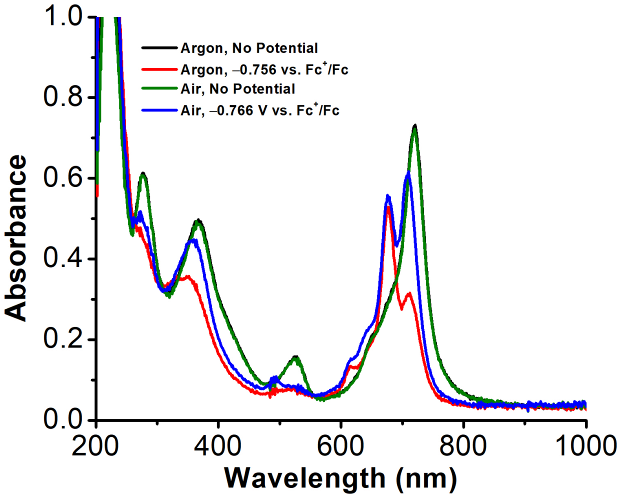

Figure S14. Comparison of the initial spectra with the spectra of the fully reduced **1Cl** species with Cl_2AcOH buffer. Conditions: 86 μM **1Cl** and 5.2 mM Cl_2AcOH/Cl_2AcO^- in 0.1 M $TBAPF_6$/MeCN.

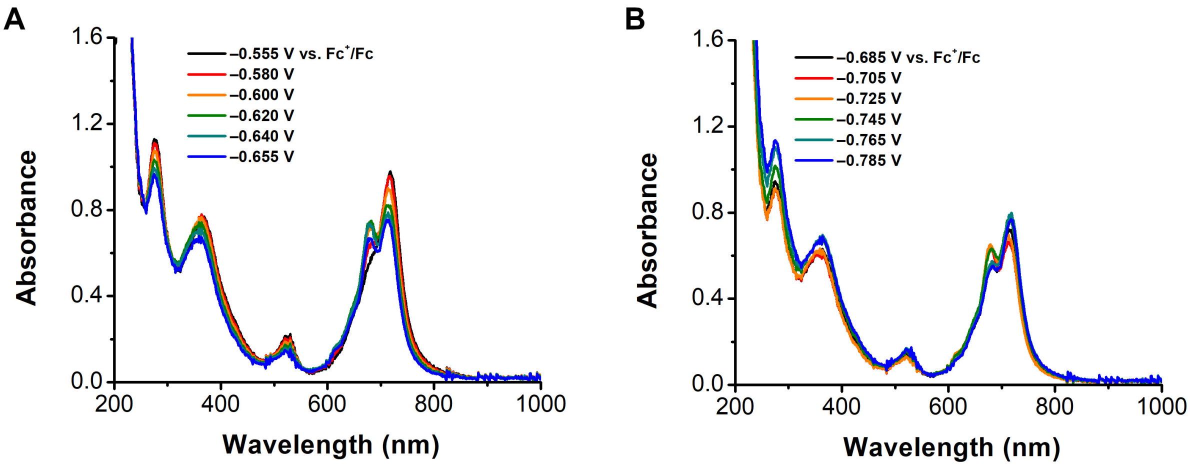

Figure S15. UV-vis showing spectral changes of **1Cl** with ClAcOH under (A) inert atmosphere and (B) ambient air with applied potentials. Conditions: 172 μM **1Cl** and 17.2 mM ClAcOH in 0.1 M $TBAPF_6$/MeCN.

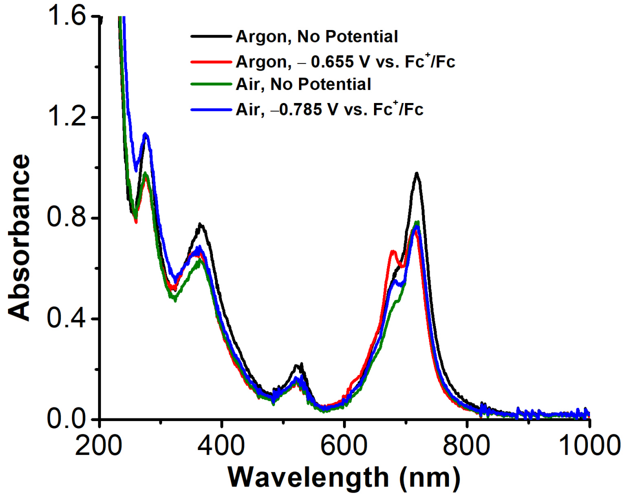

Figure S16. Comparison of the initial spectra with the spectra of the fully reduced **1Cl** species with ClAcOH. Conditions: 172 μM **1Cl** and 17.2 mM ClAcOH in 0.1 M $TBAPF_6$/MeCN.

Table S1. Summary of λ_{max} wavelengths (nm) associated with peaks in UV-vis-SEC spectra.

Acid (gas)	High-intensity $Mn^{III/II}$ (A)		Mn^{II} Shoulder (B)		Q-band (C)		High Energy Band (D)	
	No potential	Reduced	No potential	Reduced	No potential	Reduced	No potential	Reduced
**Aprotic (Ar)**	718	678	x	616	527	532	365	347
**Aprotic (air)**	718	678	x	619	529	x	364	340
**1000 eq. TFAH (Ar)**	729	x	659	x	535	x	364	340
**1000 eq. TFAH (air)**	729	x	660	x	536	x	368	x
**100 eq. Cl_2AcOH (Ar)**	720	720	x	645	525	525	366	365
**100 eq. Cl_2AcOH (air)**	720	720	x	645	525	525	366	333, 348
**20 eq. Cl_2AcOH (Ar)**	712	718, 678	x	615	493, 523	528	366	333, 348
**20 eq. Cl_2AcOH (air)**	710	711	641	x	492, 526	491	363	360
**60 eq. Buffer (Ar)**	720	677, 710	650	615	523	490, 531	368	355
**60 eq. Buffer (air)**	720	677, 708	650	615, 642	523	519	368	355
**100 eq. ClAcOH (Ar)**	718	713, 680	x	x	525	526	366	359
**100 eq. ClAcOH (air)**	718	718, 680	x	x	526	526	364	361

Electrochemical Analysis of 1Cl

Electrochemistry

Electroanalytic experiments were conducted using either a Metrhom Autolab PGSTAT302N or a BioLogic SP-50 potentiostat. RRDE experiments were performed using a Pine Research MSR Rotator and a BioLogic VSP Bipotentiostat. Glassy carbon working (⌀ = 3 mm) and non-aqueous silver/silver chloride pseudoreference electrodes behind PTFE tips were obtained from CH Instruments. The pseudoreference electrodes were obtained by depositing chloride on bare silver wire in 10% HCl at oxidizing potentials and stored in a 0.1 M tetrabutylammonium hexafluorophosphate solution in acetonitrile in the dark prior to use. The counter electrode was a glassy carbon rod (⌀ = 3 mm). All CV experiments were performed in a modified scintillation vial (20 mL volume) as a single-chamber cell with a cap modified with ports for all electrodes and a sparging needle. Tetrabutylammonium hexafluorophosphate ($TBAPF_6$) was purified by recrystallization from ethanol and dried in a vacuum oven before being stored in a desiccator. All data were referenced to an internal ferrocene standard (ferrocenium/ferrocene reduction potential under stated conditions) unless otherwise specified. All voltammograms were corrected for internal resistance. Ferrocene was purified by sublimation prior to use.

Rotating Ring-Disk Voltammetry Methods

Description of Au Ring Roughening Procedure. The Au ring electrode was roughened according to a previously reported method.[5] The electrode was first polished on a felt polishing pad with 0.3 micron alumina, then with 0.05 micron alumina and rinsed with water and ethanol. Then cyclic

voltammograms were obtained in 0.5 M H_2SO_4 by scanning from 0 to 1.6 V vs. Ag/AgCl at 100 mV/s, then at 20 mV/s for an additional two cycles to obtain the pre-roughening, surface oxide reduction charge. The electrode was then pulsed between 2.4 and 0.2 V vs. Ag/AgCl for 2.4 ms each and repeated for 250,000 cycles. Bubbles formed during electrolysis pulses were dislodged with air from a glass pipette. After electrolysis, the electrode was held at 0.3 V vs. Ag/AgCl for 2 minutes and the roughening was evaluated by CV.

Description of RRDE Collection Efficiency. The collection efficiency was determined according to previously reported methods.[6] After the Au roughening procedure was completed, a cyclic voltammogram was obtained of a ferrocene solution to determine the appropriate window for RRDE data collection. Then, the Au ring was set to 0 V and LSVs were obtained under the appropriate rotation rates (400, 800, 1200, 1600, 2000, and 2400 rpm). The solution was sparged with N_2 for 3 minutes between each scan. Conditions: 0.5 mM ferrocene in N_2-saturated, 0.1 M $TBAPF_6$/MeCN, glassy carbon working disk electrode (⌀ = 5 mm), roughened Au ring electrode, glassy carbon rod counter electrode, Ag/AgCl pseudoreferenec electrode, 0.02 mV/s and 0.1 mV/s. To calculate the collection efficiency of the RRDE, the ratio of the ring current (i_r) to the disk current (i_d) at each rotation rate was used to determine $N_{emperical}$ (**Eq. S6**). The $N_{emperical}$ value at each rotation rate was multiplied by a factor of 100 to determine the collection efficiency at each rotation rate (~22%).

$$N_{emperical} = \frac{i_{ring}}{i_{disk}} \qquad \textbf{(Eq. S6)}$$

Description of RRDE Experiment. After the Au roughening procedure was completed, a standard CV of **1Cl** under catalytic conditions was obtained to determine the appropriate potential window for the experiment. Then, the Au ring was set to +1.2 V, and LSVs were obtained for the same rotation rates used in the collection efficiency procedure. The solution was sparged with air for 3 minutes between each scan, and the glassy carbon disk electrode was polished with felt. The reproducibility of the data was confirmed by repeating scans at the same rotation rates, producing exact overlays. The same procedure was repeated under N_2. The disk and ring currents were corrected by subtracting the limiting current observed under inert conditions from the limiting current under catalytic conditions. Levich analysis (**Figures S36 and S43**) was used to verify the suitability of the quantification method.

The arithmetic mean of the number of electrons received by O_2 (n_{cat}) during the ORR was calculated from the disk current (i_d) and ring current (i_r) according to **Eq. S7**:[7]

$$n_{cat} = 4 \times \frac{i_d}{i_d + \frac{i_r}{N_{emperical}}} \qquad \textbf{(Eq. S7)}$$

The H_2O_2 ratio (p) is defined as the fraction of O_2 reduced to H_2O_2 and related to n_{cat} by **Eq. S8**:

$$n_{cat} = 4 - 2p \qquad \textbf{(Eq. S8)}$$

Multiplying p by 100 provides the % H_2O_2 selectivity of the ORR.

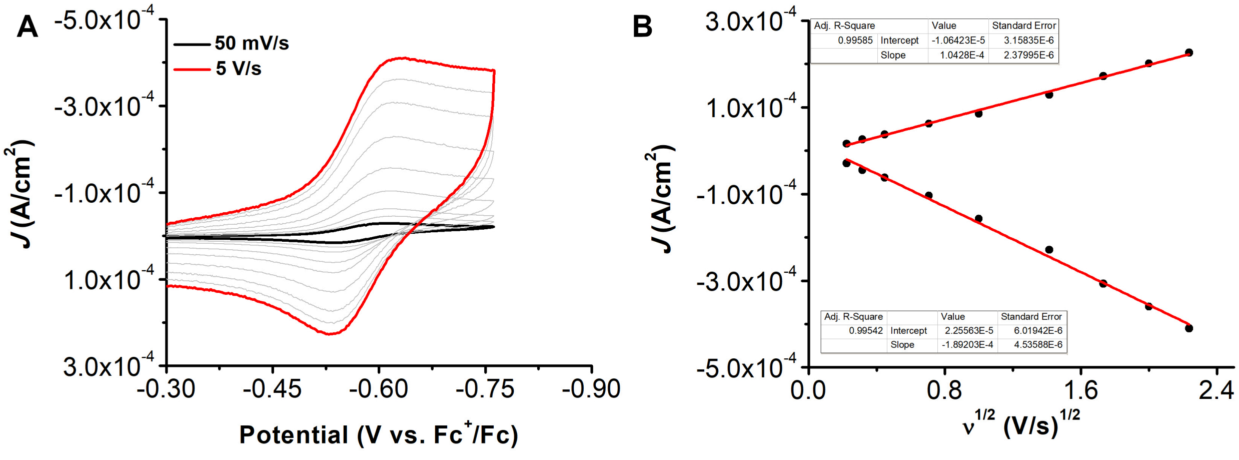

Figure S17. (A) CVs of **1Cl** under inert atmosphere at variable scan rate and (B) plot showing the linear dependence of current density on the square root of scan rate. Conditions: 0.5 mM **1Cl**, 0.1 M $TBAPF_6$/MeCN, glassy carbon working electrode, glassy carbon rod counter electrode, Ag/AgCl pseudoreference electrode, referenced to an internal ferrocene standard, scan rates: 0.05, 0.1, 0.2, 0.5, 0.75, 1, 2, 3, 4, 5 V/s.

The diffusion coefficient of **1Cl** was calculated using the slope from **Figure S17.B and Eq. S9-S10**, where i_p is the current (A), n is the number of electrons, F is Faraday's constant (96,485 A•s/mol), A is the area of the electrode (cm^2), C is the concentration of the bulk analyte solution (mol/cm^3), R is the gas constant (8.314 V•A•s/K•mol), and T is the temperature (K).[8]

$$i_p = 0.4463\, nFAC\sqrt{\frac{nFvD}{RT}} \qquad \textbf{(Eq. S9)}$$

$$D = \frac{m^2RT}{(0.4463AC)^2(nF)^3} \qquad \textbf{(Eq. S10)}$$

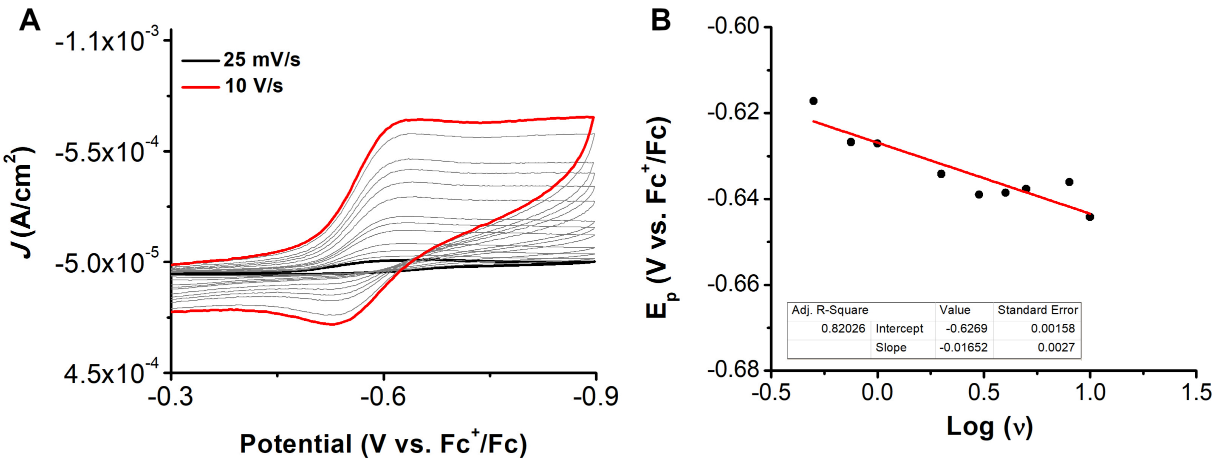

Figure S18. (A) CVs of **1Cl** under Oxygen-saturation at variable scan rate and (B) plot showing the linear dependence of peak voltage on the log of scan rate. Conditions: 0.5 **1Cl**, 0.1 M $TBAPF_6$/MeCN, glassy carbon working electrode, glassy carbon rod counter electrode, Ag/AgCl pseudoreference electrode, referenced to an internal ferrocene standard, scan rates: 0.025, 0.05, 0.1, 0.2, 0.5, 0.75, 1, 2, 3, 4, 5 V/s.

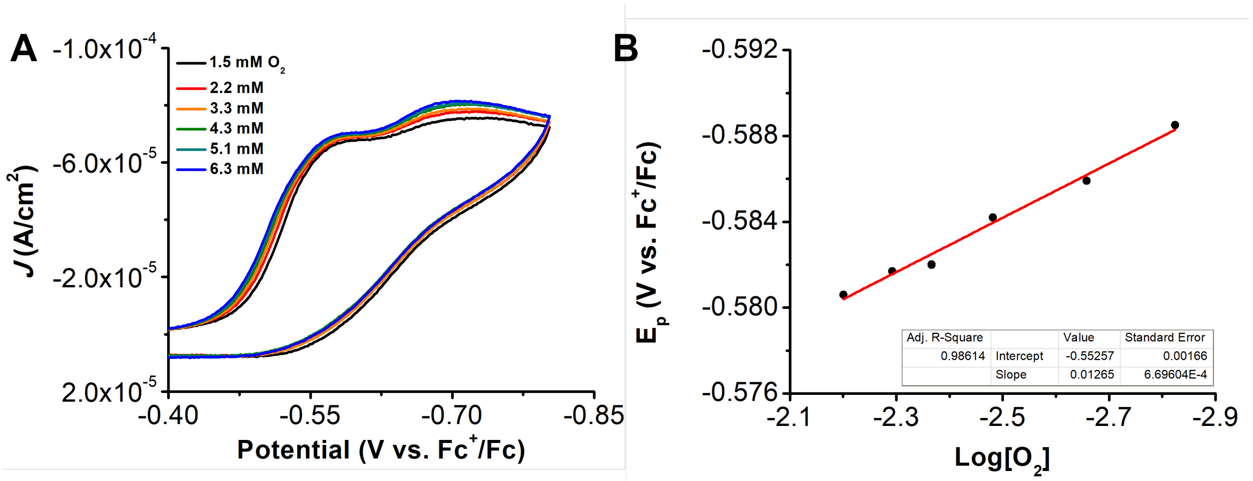

Figure S19. (A) CVs of **1Cl** variable O_2 concentrations. (B) Log-Log plot of oxygen concentration versus peak current for the first reduction. Conditions: 0.5 mM **1Cl**, 0.1 M $TBAPF_6$/MeCN, glassy carbon working electrode, glassy carbon rod counter electrode, Ag/AgCl pseudoreference electrode, referenced to an internal ferrocene standard, 100 mV/s scan rate.

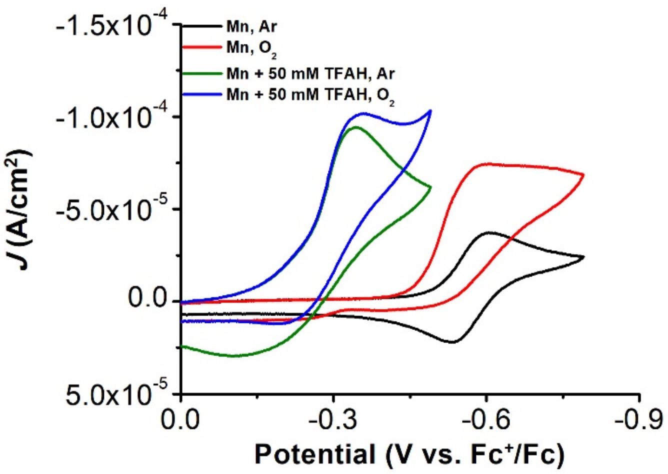

Figure S20. CVs of **1Cl** under Ar (black), O_2 (red) and with 50 mM TFAH under Ar (green) and oxygen-saturated (blue) conditions. Conditions: 0.5 mM **1Cl**, 0.1 M $TBAPF_6$/MeCN, glassy carbon working electrode, glassy carbon rod counter electrode, Ag/AgCl pseudoreference electrode, referenced to an internal ferrocene standard, 100 mV/s scan rate.

Electrochemical Studies with Cl_2AcOH

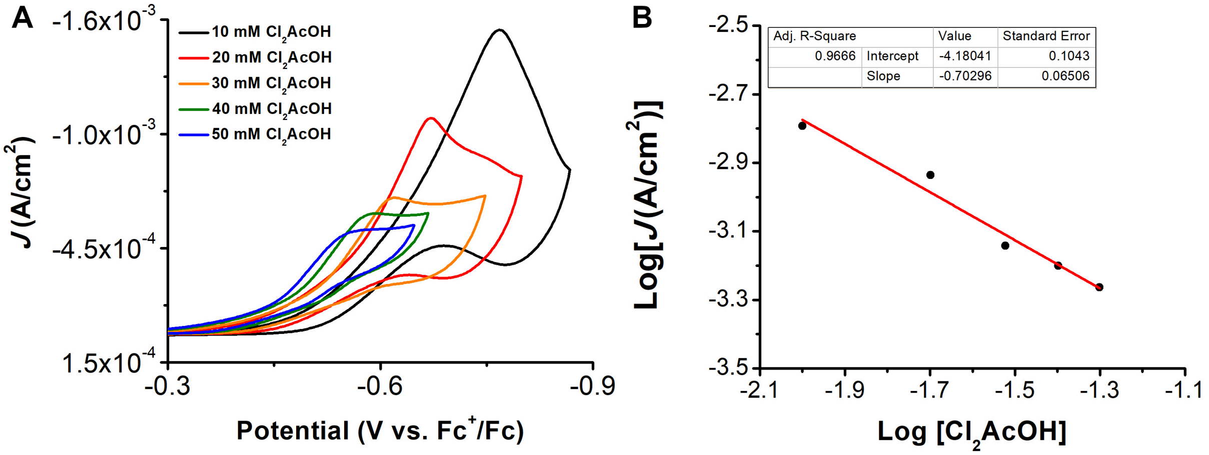

Figure S21. (A) CVs of **1Cl** under catalytic conditions with variable Cl_2AcOH concentrations. (B) Log-Log plot of the log of the acid concentration versus the catalytic current density. Conditions: 0.5 mM **1Cl**, 0.1 M $TBAPF_6$/MeCN, glassy carbon working electrode, glassy carbon rod counter electrode, Ag/AgCl pseudoreference electrode, referenced to an internal ferrocene standard, 100 mV/s scan rate.

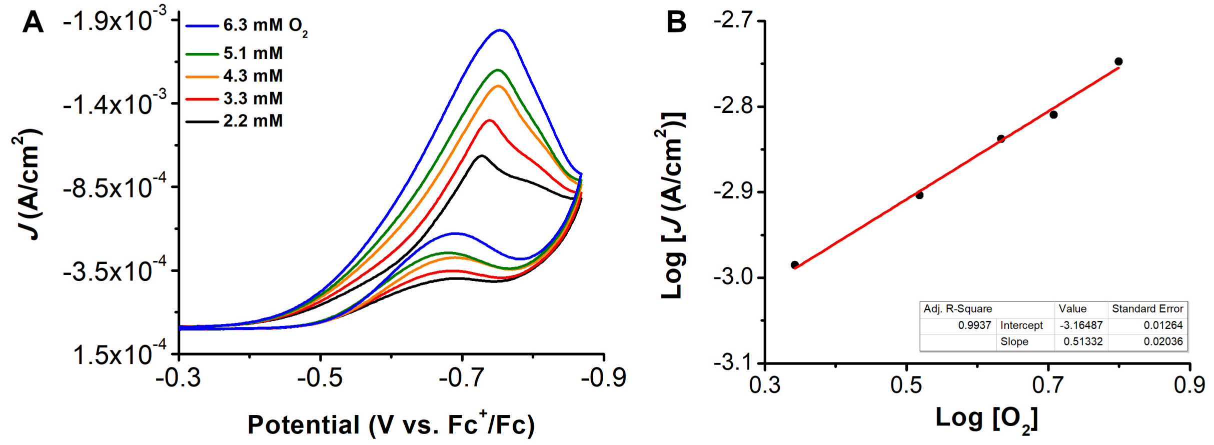

Figure S22. (A) CVs of **1Cl** under catalytic conditions with variable O_2 concentrations. (B) Log-Log plot of Oxygen concentration versus the catalytic current density. Conditions: 0.5 mM **1Cl**, 10 mM Cl_2AcOH, 0.1 M $TBAPF_6$/MeCN, glassy carbon working electrode, glassy carbon rod counter electrode, Ag/AgCl pseudoreference electrode, referenced to an internal ferrocene standard, 100 mV/s scan rate.

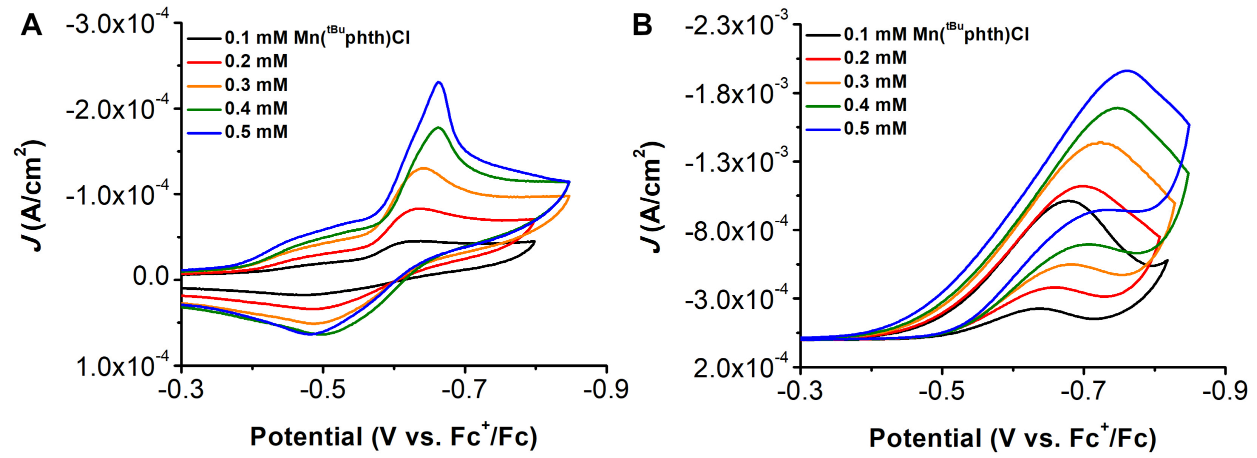

Figure S23. CVs of **1Cl** under inert (A) and (B) catalytic conditions with variable **1Cl** concentrations. Conditions: 10 mM Cl_2AcOH, 0.1 M $TBAPF_6$/MeCN, glassy carbon working electrode, glassy carbon rod counter electrode, Ag/AgCl pseudoreference electrode, referenced to an internal ferrocene standard, 100 mV/s scan rate.

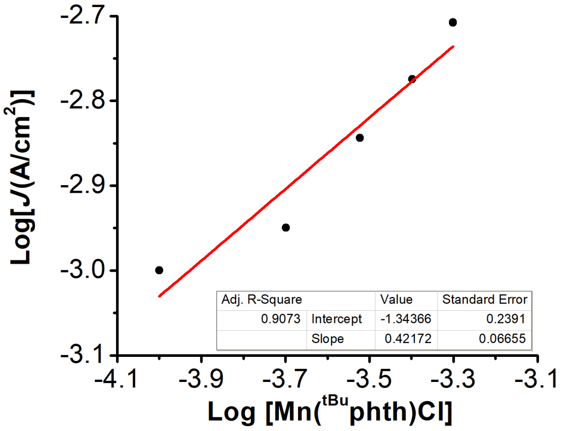

Figure S24. Log-Log plot of **1Cl** concentration versus the catalytic current density.

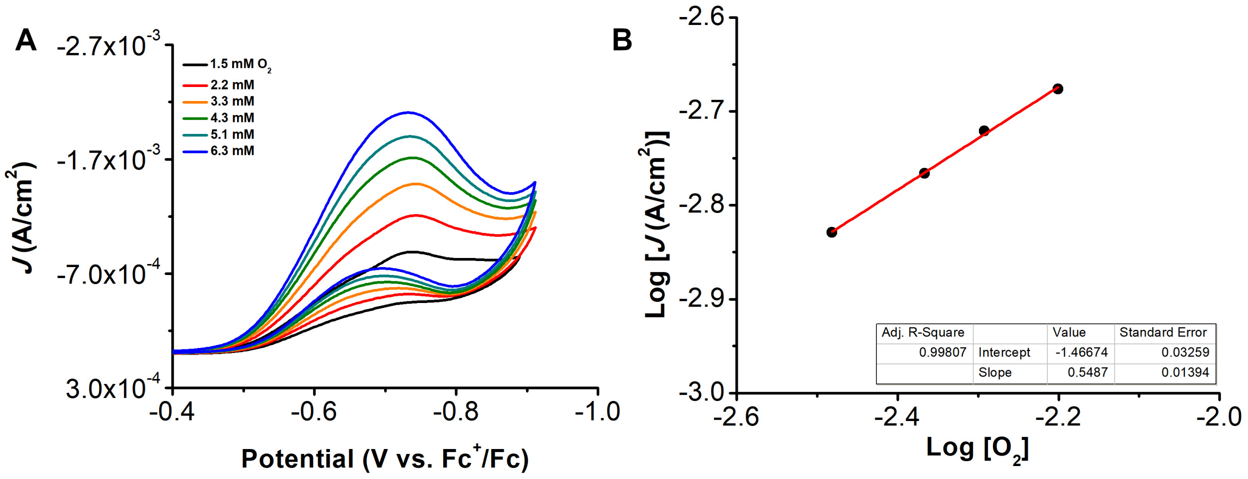

Figure S25. (A) CVs of **1Cl** under catalytic conditions with variable O_2 concentrations. (B) Log-Log plot of Oxygen concentration versus the catalytic current density. Conditions: 0.5 mM **1Cl**, 50 mM Cl_2AcOH, 0.1 M $TBAPF_6$/MeCN, glassy carbon working electrode, glassy carbon rod counter electrode, Ag/AgCl pseudoreference electrode, referenced to an internal ferrocene standard, 100 mV/s scan rate.

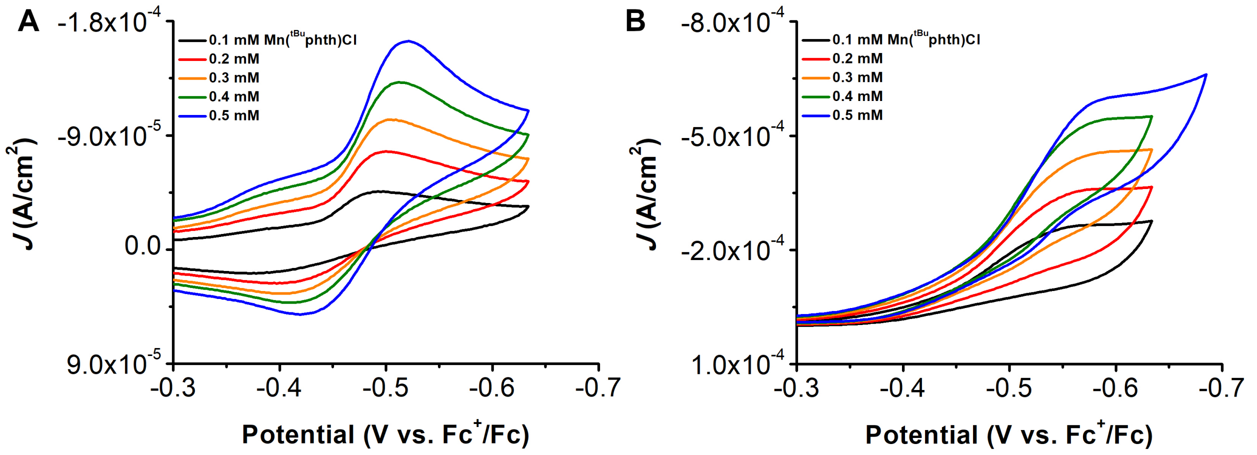

Figure S26. CVs of **1Cl** under inert (A) and (B) catalytic conditions with variable Mn(^{tBu}phthalocyanine)Cl concentrations. Conditions: 50 mM Cl_2AcOH, 0.1 M $TBAPF_6$/MeCN, glassy carbon working electrode, glassy carbon rod counter electrode, Ag/AgCl pseudoreference electrode, referenced to an internal ferrocene standard, 100 mV/s scan rate.

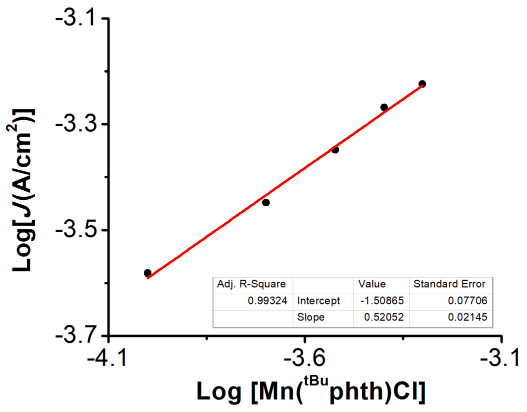

Figure S27. Log-Log plot of **1Cl** concentration versus the catalytic current density.

Electrochemical Studies with Cl_2AcOH/Cl_2AcO^- buffer.

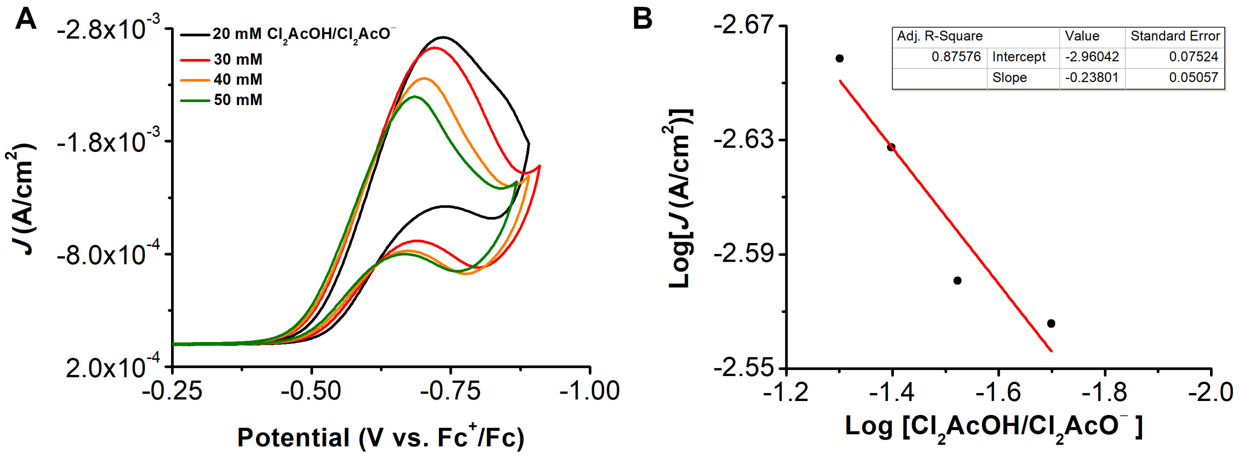

Figure S28. (A) CVs of **1Cl** under catalytic conditions with variable Cl_2AcOH/Cl_2AcO^- concentrations. (B) Log-Log plot of the log of the buffer concentration versus the catalytic current density. Conditions: 0.5 mM **1Cl**, 0.1 M $TBAPF_6$/MeCN, glassy carbon working electrode, glassy carbon rod counter electrode, Ag/AgCl pseudoreference electrode, referenced to an internal ferrocene standard, 100 mV/s scan rate.

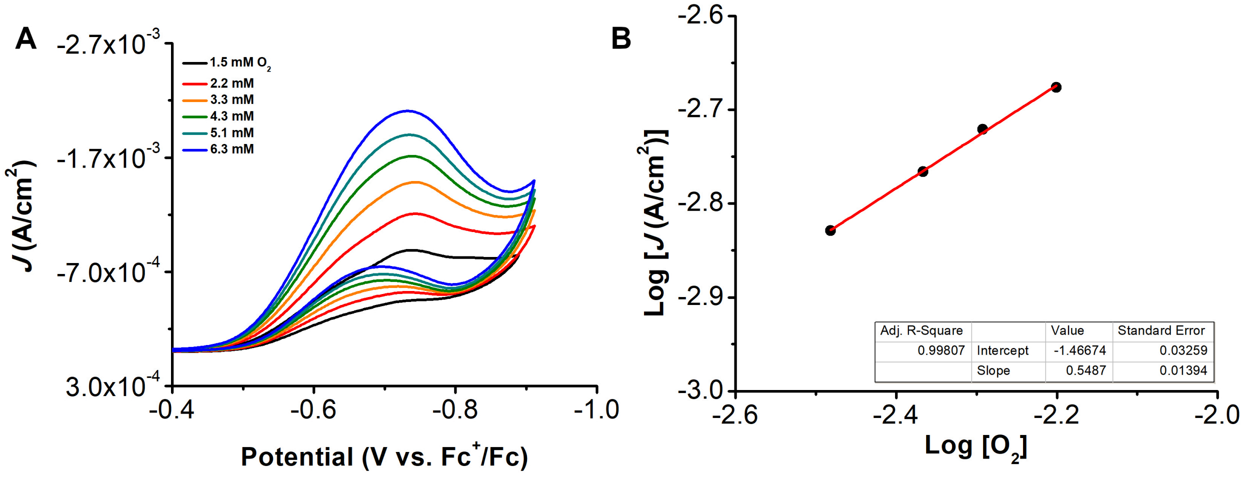

Figure S29. (A) CVs of **1Cl** under catalytic conditions with variable O_2 concentrations. (B) Log-Log plot of Oxygen concentration versus the catalytic current density. Conditions: 0.5 mM **1Cl**, 30 mM $Cl_2AcOH/Cl2AcO^-$, 0.1 M $TBAPF_6$/MeCN, glassy carbon working electrode, glassy carbon rod counter electrode, Ag/AgCl pseudoreference electrode, referenced to an internal ferrocene standard, 100 mV/s scan rate.

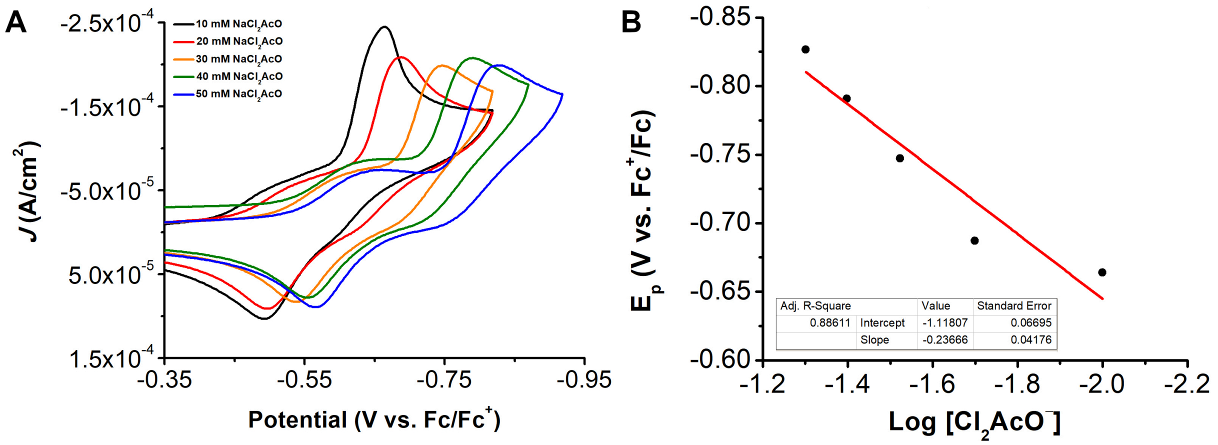

Figure S30. (A) CVs of **1Cl** under inert atmosphere with variable NaCl$_2$AcO concentrations. (B) Plot of the log of the NaCl$_2$AcO concentration versus the reduction peak potential. Conditions: 0.5 mM **1Cl**, 30 mM Cl$_2$AcOH, 0.1 M TBAPF$_6$/MeCN, glassy carbon working electrode, glassy carbon rod counter electrode, Ag/AgCl pseudoreference electrode, referenced to an internal ferrocene standard, 100 mV/s scan rate.

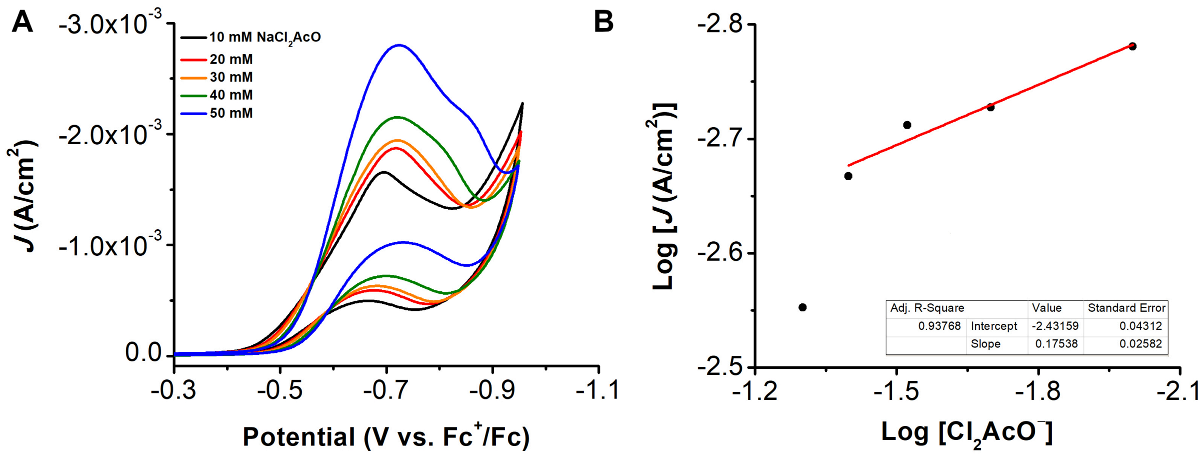

Figure S31. (A) CVs of **1Cl** under catalytic conditions with variable NaCl$_2$AcO concentrations. (B) Log-Log plot of the log of the NaCl$_2$AcO concentration versus the catalytic current density. Conditions: 0.5 mM **1Cl**, 30 mM Cl$_2$AcOH, 0.1 M TBAPF$_6$/MeCN, glassy carbon working electrode, glassy carbon rod counter electrode, Ag/AgCl pseudoreference electrode, referenced to an internal ferrocene standard, 100 mV/s scan rate.

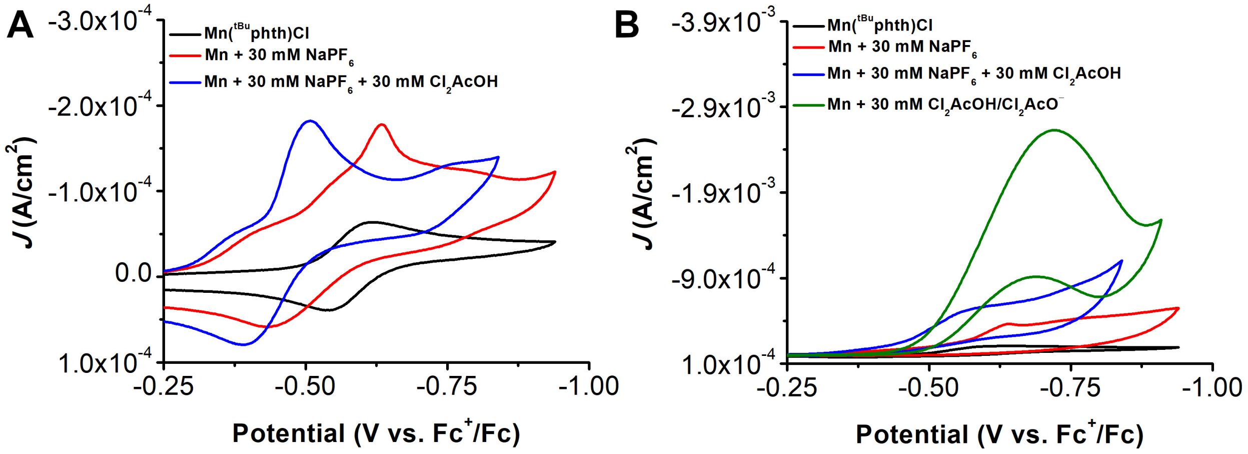

Figure S32. (A) CVs of **1Cl** under inert conditions with $NaPF_6$ (red trace) and Cl_2AcOH (blue trace). (B) CVs of **1Cl** under catalytic conditions with NaPF6 (red trace), Cl_2AcOH (blue trace), and buffer (green trace). Conditions: 0.5 mM **1Cl**, 30 mM $NaPF_6$, 30 mM Cl_2AcOH, 0.1 M $TBAPF_6$/MeCN, glassy carbon working electrode, glassy carbon rod counter electrode, Ag/AgCl pseudoreference electrode, referenced to an internal ferrocene standard, 100 mV/s scan rate.

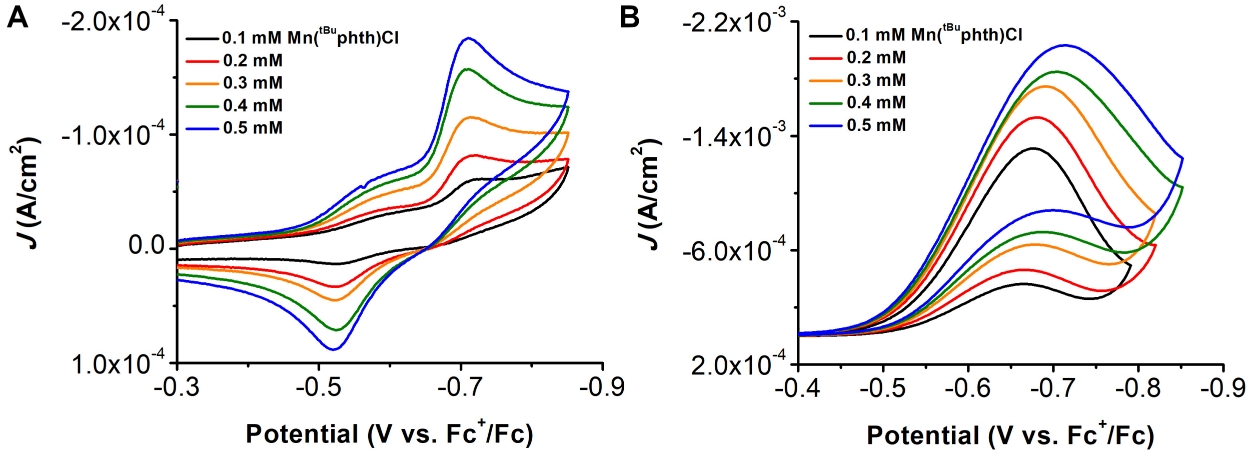

Figure S33. CVs of **1Cl** under inert (A) and (B) catalytic conditions with variable **1Cl** concentrations. Conditions: 30 mM Cl_2AcOH/Cl_2AcO^-, 0.1 M $TBAPF_6$/MeCN, glassy carbon working electrode, glassy carbon rod counter electrode, Ag/AgCl pseudoreference electrode, referenced to an internal ferrocene standard, 100 mV/s scan rate.

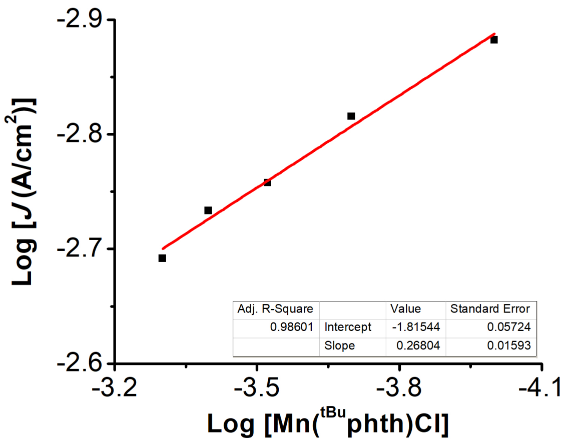

Figure S34. Log-Log plot of **1Cl** concentration versus the catalytic current density from Figure **S33.B.**

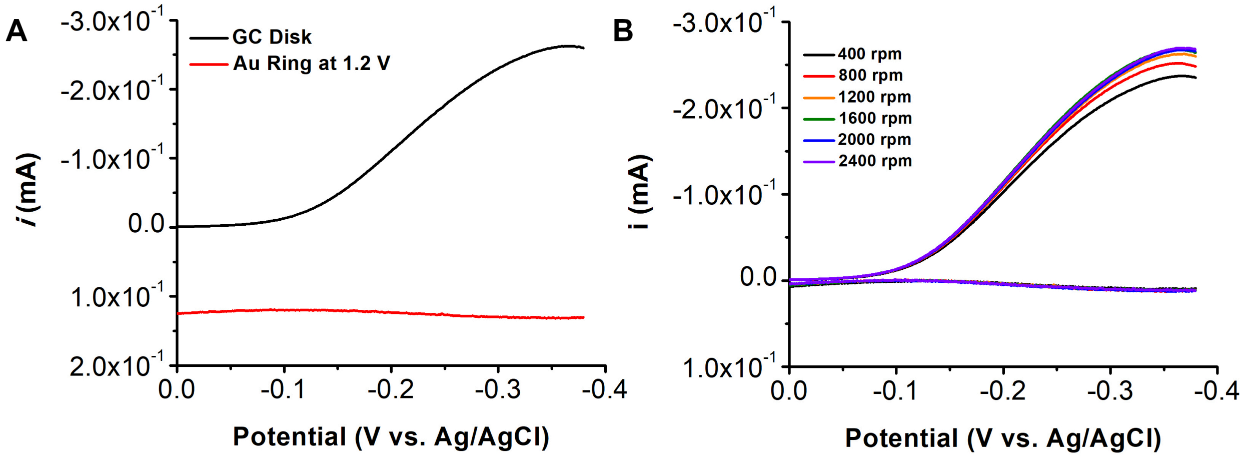

Figure S35. Linear sweep voltammograms of the RRDE experiment with 0.5 mM **1Cl** and 30 mM Cl_2AcOH/Cl_2AcO^- under air saturation. (A) Uncorrected LSVs at 1200 rpm. (B) Corrected LSVs at various rotation rates used for product quantification of % H_2O_2. Ring potential = 1.2 V vs. Ag/AgCl. Conditions: 0.5 mM **1Cl**, 0.1 M $TBAPF_6$/MeCN, glassy carbon working electrode/Au roughened working electrode, glassy carbon rod counter electrode, Ag/AgCl pseudoreference electrode, scan rate 20 mV/s.

Calculated ORR selectivity under electrochemical conditions using RRDE is 0.04 ± 1.8 % H_2O_2.

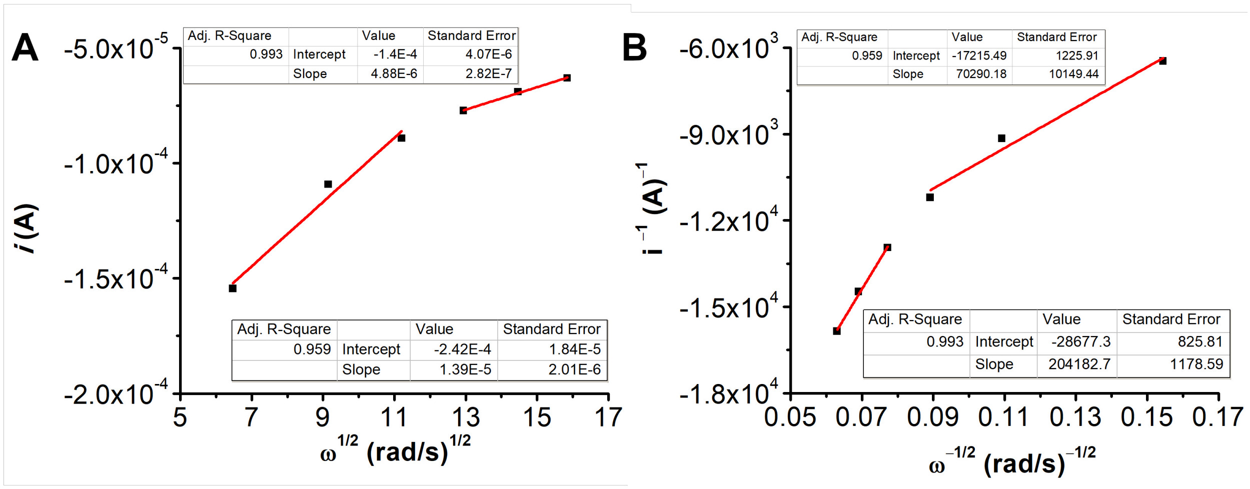

Figure S36. (A) Levich and (B) Koutecky-Levich plots from data obtained from linear sweep voltammograms of **1Cl** (0.5 mM) by RRDE with 30 mM Cl_2AcOH/Cl_2AcO^- under air saturation at various rotation rates.

Electrochemical Studies with ClAcOH

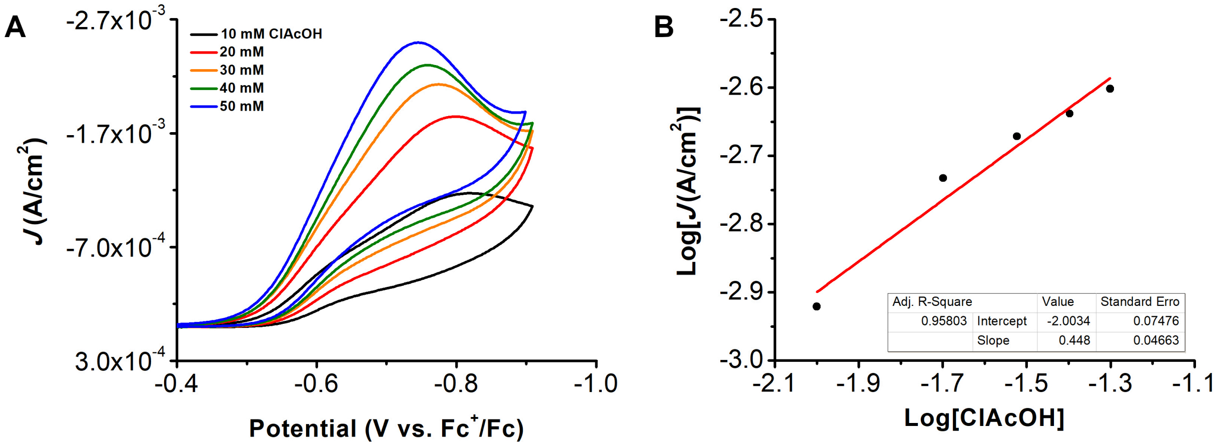

Figure S37. (A) CVs of **1Cl** under catalytic conditions with variable ClAcOH concentrations. (B) Log-Log plot of the log of the acid concentration versus the catalytic current density. Conditions: 0.5 mM **1Cl**, 0.1 M $TBAPF_6$/MeCN, glassy carbon working electrode, glassy carbon rod counter electrode, Ag/AgCl pseudoreference electrode, referenced to an internal ferrocene standard, 100 mV/s scan rate.

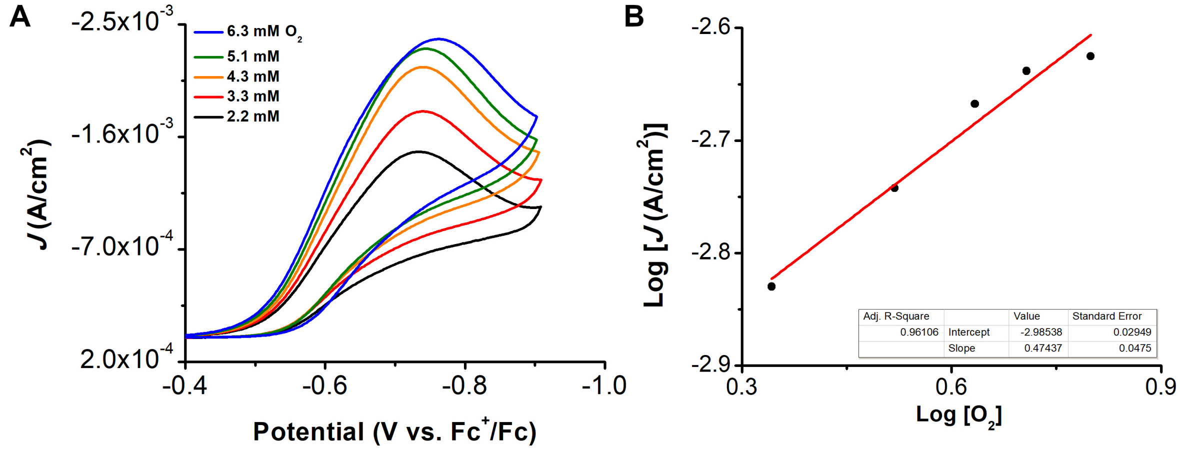

Figure S38. (A) CVs of **1Cl** under catalytic conditions with variable O_2 concentrations. (B) Log-Log plot of Oxygen concentration versus the catalytic current density. Conditions: 0.5 mM **1Cl**, 50 mM ClAcOH, 0.1 M $TBAPF_6$/MeCN, glassy carbon working electrode, glassy carbon rod counter electrode, Ag/AgCl pseudoreference electrode, referenced to an internal ferrocene standard, 100 mV/s scan rate.

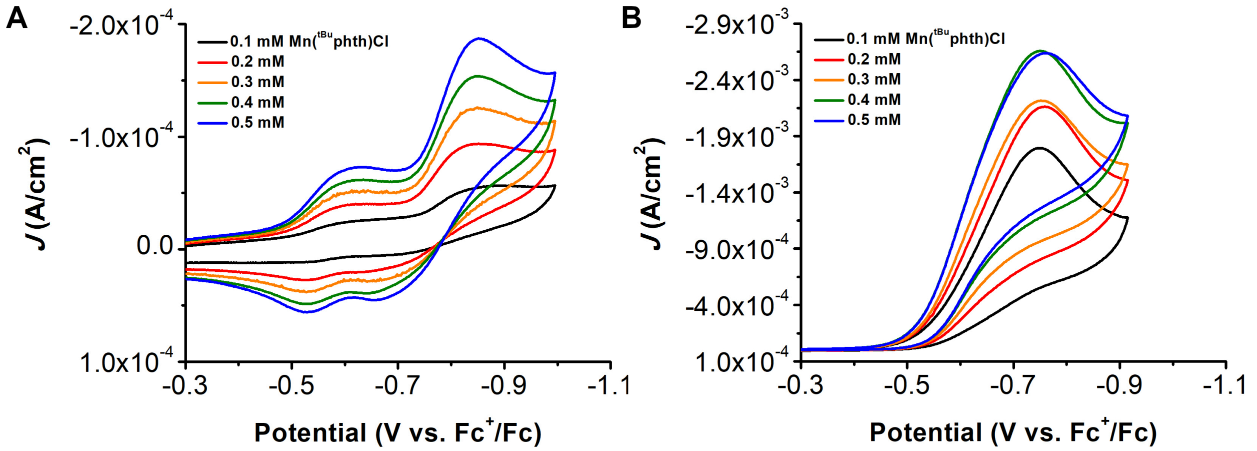

Figure S39. CVs of **1Cl** under inert (A) and (B) catalytic conditions with variable **1Cl** concentrations. Conditions: 50 mM ClAcOH, 0.1 M $TBAPF_6$/MeCN, glassy carbon working electrode, glassy carbon rod counter electrode, Ag/AgCl pseudoreference electrode, referenced to an internal ferrocene standard, 100 mV/s scan rate.

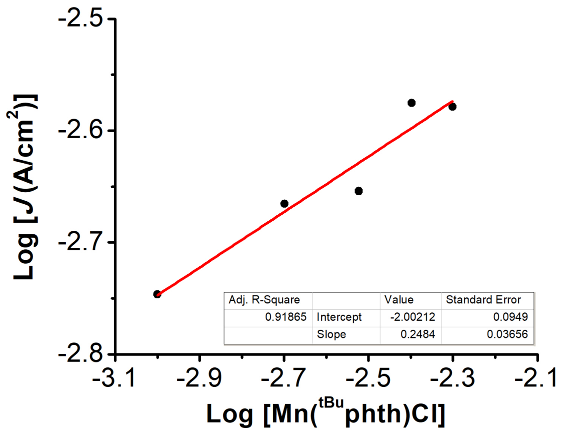

Figure S40. Log-Log plot of **1Cl** concentration versus the catalytic current density from Fi**gure S39.B.**

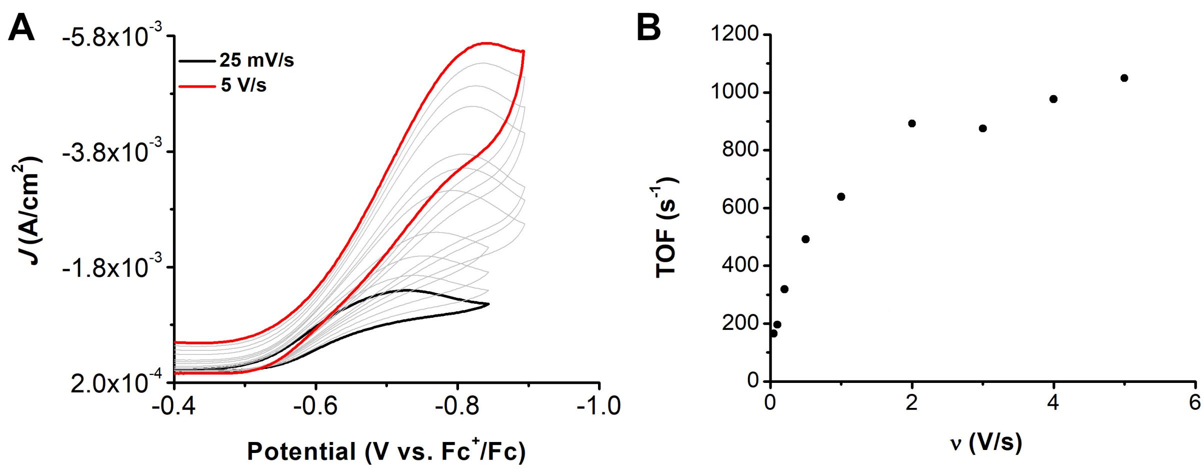

Figure S41. Determination of turnover frequency (TOF). (A) Variable scan rate CVs of **1Cl** with ClAcOH under O_2-saturation. (B) Calculated TOF from each scan rate versus the scan rate. Conditions: 0.5 mM **1Cl**, 50 mM ClAcOH in O_2-saturated 0.1 M $TBAPF_6$/MeCN; glassy carbon working electrode, glassy carbon rod counter electrode, Ag/AgCl pseudoreference electrode; referenced to and internal ferrocene standard.

Calculation of TOF. The TOF (s^{-1}) was calculated according to previously reported methods using **Eq. S11**.[8]

$$TOF = k_{obs} = k_{cat}[Q] = \frac{Fvn_p^3}{RT}\left(\frac{0.4463}{n_{cat}}\right)^2\left(\frac{i_{cat}}{i_p}\right)^2 \qquad \textbf{(Eq. S11)}$$

where n_p is the number of electrons needed to form the active catalyst from the precatalyst, ν is the scan rate (V/s) F is Faraday's constant (96,485 A s/mol) R is the gas constant (8.314 V A s/K mol), T is the temperature (K), n_{cat} is the arithmetic mean of the number of electrons received by O_2 during the ORR, i_{cat} is the catalytic peak current, and i_p is the noncatalytic peak current.

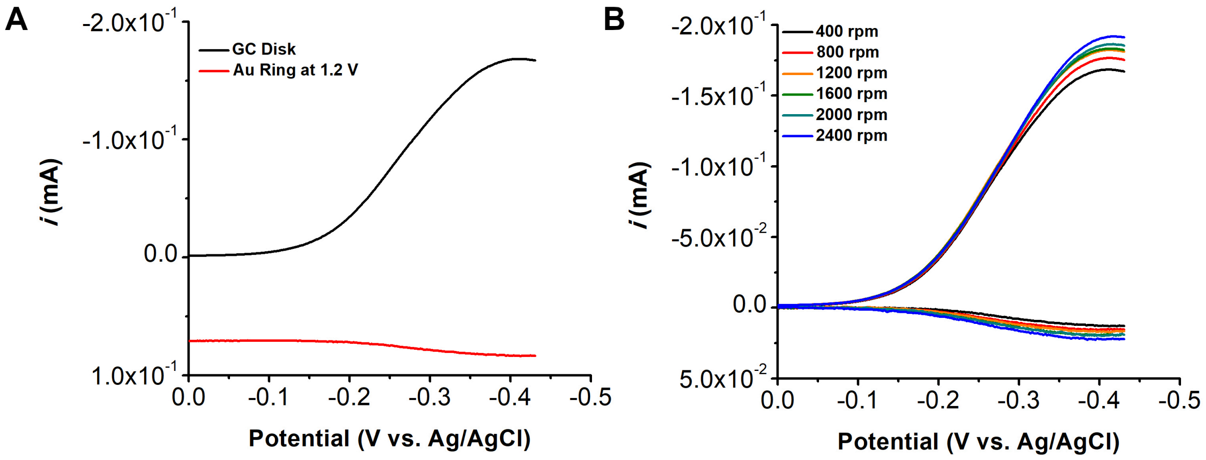

Figure S42. Linear sweep voltammograms of the RRDE experiment with 0.5 mM **1Cl** and 50 mM ClAcOH under air saturation. (A) Uncorrected LSVs at 1200 rpm. (B) Corrected LSVs at various rotation rates used for product quantification of % H_2O_2. Ring potential = 1.2 V vs. Ag/AgCl. Conditions: 0.5 mM **1Cl**, 0.1 M $TBAPF_6$/MeCN, glassy carbon working electrode/Au roughened working electrode, glassy carbon rod counter electrode, Ag/AgCl pseudoreference electrode, scan rate 100 mV/s.

Calculated ORR selectivity under electrochemical conditions using RRDE is 59.5 ± 6.4 % H_2O_2.

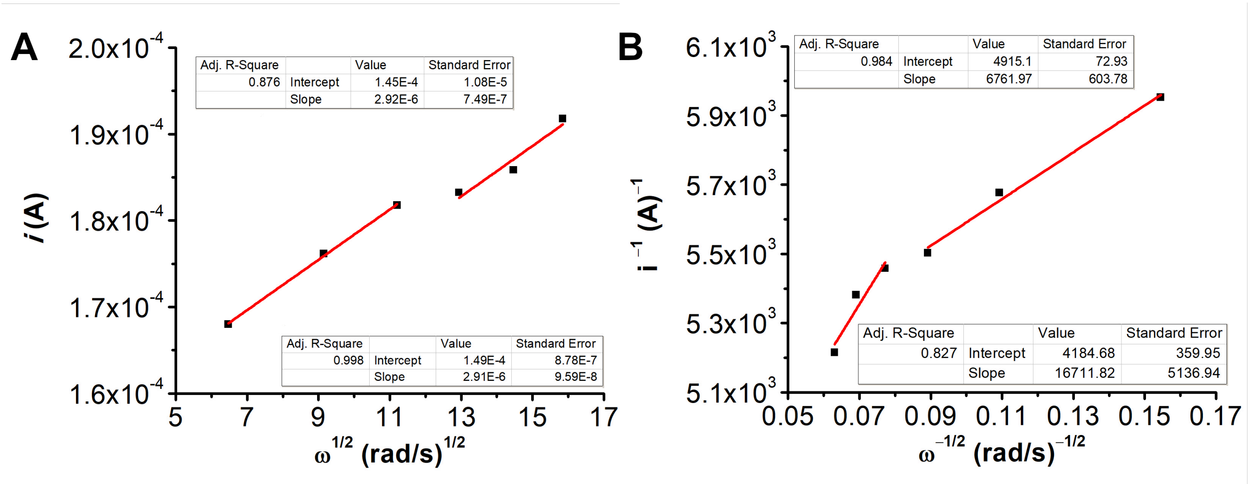

Figure S43. (A) Levich and (B) Koutecky-Levich plots from data obtained from linear sweep voltammograms of **1Cl** (0.5 mM) by RRDE with 50 mM ClAcOH under air saturation at various rotation rates.

Stopped-Flow Kinetic Analysis of 1Cl

Stopped-flow spectrochemical kinetic studies were performed with a CSF-61DX2 Stopped-Flow System from Hi-Tech Scientific. Kinetic Studio Software was used to monitor a single wavelength. All data fits were performed within the Kinetic Studio 4.0 Software Suite. Prior to experiments, dried MeCN was passed through the syringes and cell block before the reagents were loaded. In the experiment, syringes would be charged with known concentrations of reagent. All reagent solutions were prepared immediately before use. The system was flushed with dried MeCN between each trial. All studies were conducted at 25 °C unless otherwise stated.

In general, a vial containing **1Cl** catalyst and acid was sparged with O_2, drawn into a syringe, and loaded into the stopped-flow system. A second syringe containing Ar-sparged $Cp^*{}_2Fe$ was loaded into the stopped-flow system. All concentrations reported are the mixed concentrations in the spectroscopic cell.

R_{fit} values (as R_1 in fitting function) were obtained by fitting the data in the Kinetic Studio 4.0 Software to a double exponential (2Exp+Mx+C) to achieve an R^2 value of 0.999. The R_{fit} values were corrected for the number of electrons passed during catalysis (n_{cat} = 2.0 for all acids) and plotted against variable concentrations to obtain the experimental rate law (**Eq. 1–3**, main text).

Stopped-Flow with ClAcOH

$$\frac{R_{fit}}{n_{cat}} = k_{cat}[\mathbf{1Cl}]^1[O_2]^1[Cp^*{}_2Fe]^{variable}$$

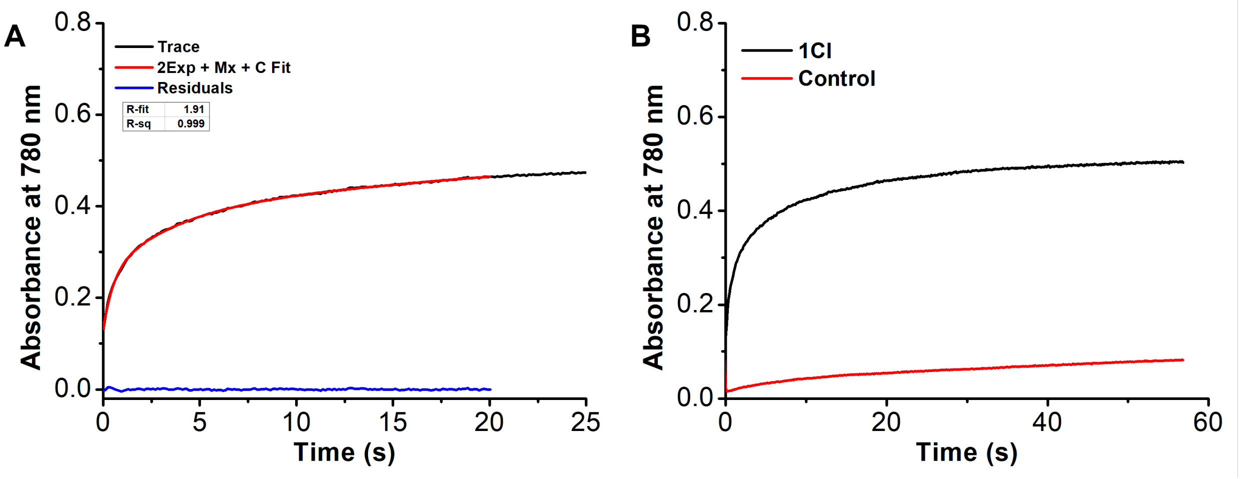

Figure S44. (A) Change in absorbance at 780 nm over time due to the formation of $[Cp^*{}_2Fe]^+$ by ORR catalyzed by **1Cl** with ClAcOH (black trace), exampled of a 2Exp + Mx + C fit in Kinetic Studio 4.0 (red trace), and residual fit (blue trace). (B) Black trace from (A) and ClAcOH only control (red trace, no **1Cl** present). Concentrations: **1Cl** (if present) = 10 µM, ClAcOH = 40 mM, O_2 = 4.05 mM, $Cp^*{}_2Fe$ = 1 mM.

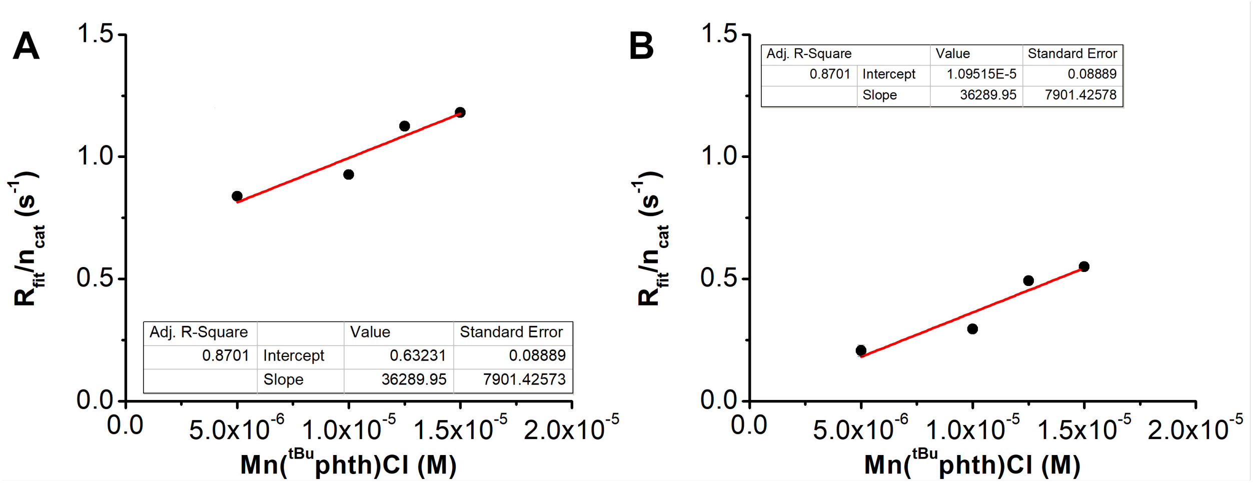

Figure S45. Calculated R_{fit}/n_{cat} values from stopped-flow spectrochemical experiments with ClAcOH, O_2, and $Cp^*{}_2Fe$ with varying **1Cl** concentrations. (B) Corrected for background by subtracting the intercept from (A). Data were fit using Kinetic Studio 4.0 (2Exp + Mx + C); n_{cat} = 2.07. Concentrations: ClAcOH = 40 mM, O_2 = 4.05 mM, $Cp^*{}_2Fe$ = 1 mM.

Calculation of Turnover Frequency. The turnover frequency per equivalent of Mn can be defined by the slope of the variable [**1Cl**] plot when there is a first-order dependence on **1Cl** (**Figure S45**).[9, 10] With ClAcOH, TOF can be calculated using **Eq. S12** and the slope from **Figure S45.B.**

$$\frac{R_{fit}}{n_{cat}} = k_{cat}[\mathbf{1Cl}]^1[O_2]^1[Cp^*{}_2Fe]^{variable} = TOF_{ORR}[1Cl]^1 \qquad \textbf{(Eq. S12)}$$

$$TOF_{ORR} = 3.63 \times 10^4\ s^{-1}$$

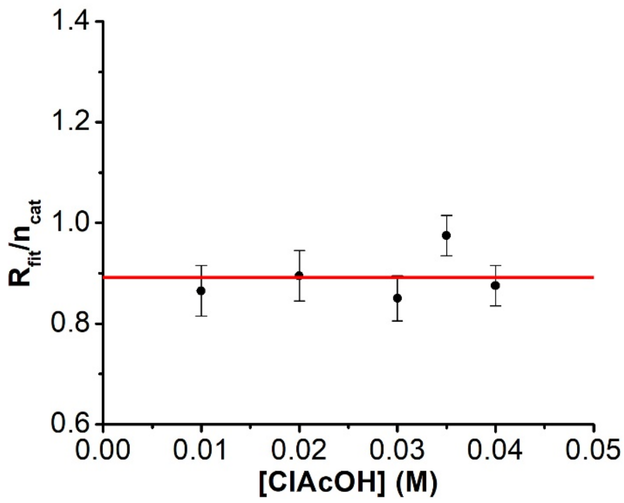

Figure S46. Calculated R_{fit}/n_{cat} values from stopped-flow spectrochemical experiments with **1Cl**, O_2, and $Cp^*{}_2Fe$ with varying ClAcOH concentrations. Data were fit using Kinetic Studio 4.0 (2Exp + Mx + C); n_{cat} = 2.07. Concentrations: **1Cl** = 10 μM, O_2 = 4.05 mM, $Cp^*{}_2Fe$ = 1 mM.

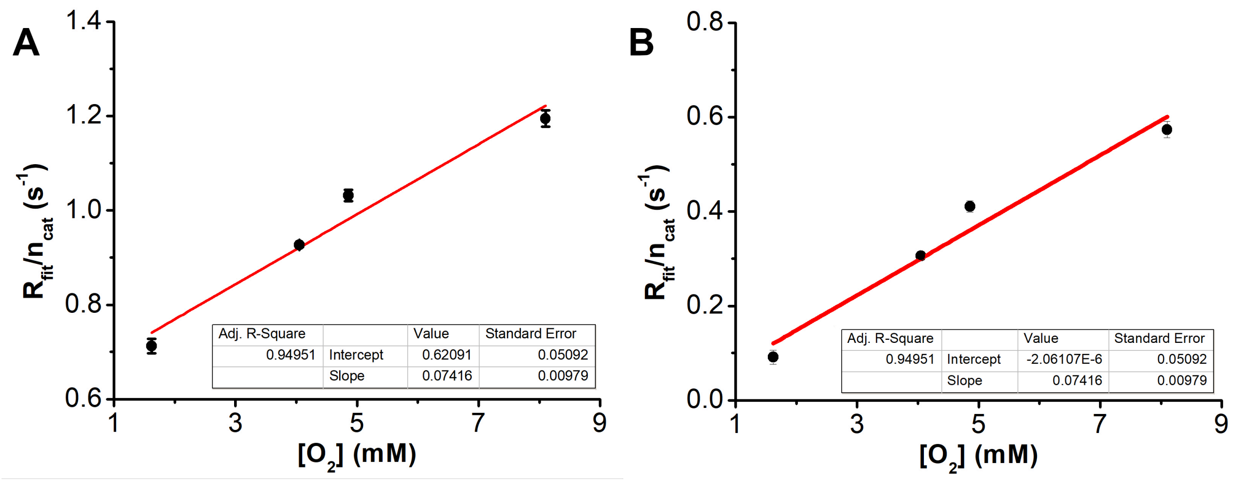

Figure S47. Calculated R_{fit}/n_{cat} values from stopped-flow spectrochemical experiments with ClAcOH, **1Cl**, and $Cp^*{}_2Fe$ with varying O_2 concentrations. (B) Corrected for background by subtracting the intercept from (A). Data were fit using Kinetic Studio 4.0 (2Exp + Mx + C); n_{cat} = 2.07. Concentrations: **1Cl** = 10 μM, ClAcOH = 40 mM, $Cp^*{}_2Fe$ = 1 mM.

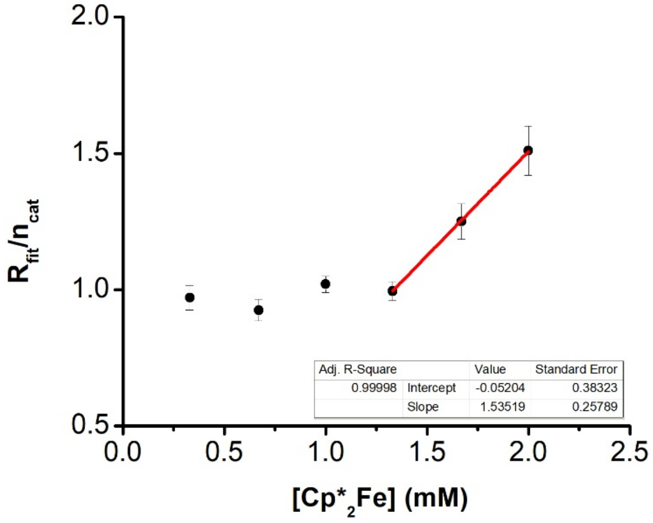

Figure S48. Calculated R_{fit}/n_{cat} values from stopped-flow spectrochemical experiments with **1Cl**, ClAcOH, and O_2 with varying $Cp^*{}_2Fe$ concentrations. Data were fit using Kinetic Studio 4.0 (2Exp + Mx + C); n_{cat} = 2.07. Concentrations: **1Cl** = 10 µM, ClAcOH = 40 mM, O_2 = 4.05 mM.

Stopped-Flow with Cl_2AcOH

$$\frac{R_{fit}}{n_{cat}} = k_{cat}[\mathbf{1Cl}]^1[Cl_2AcOH]^{inverse}$$

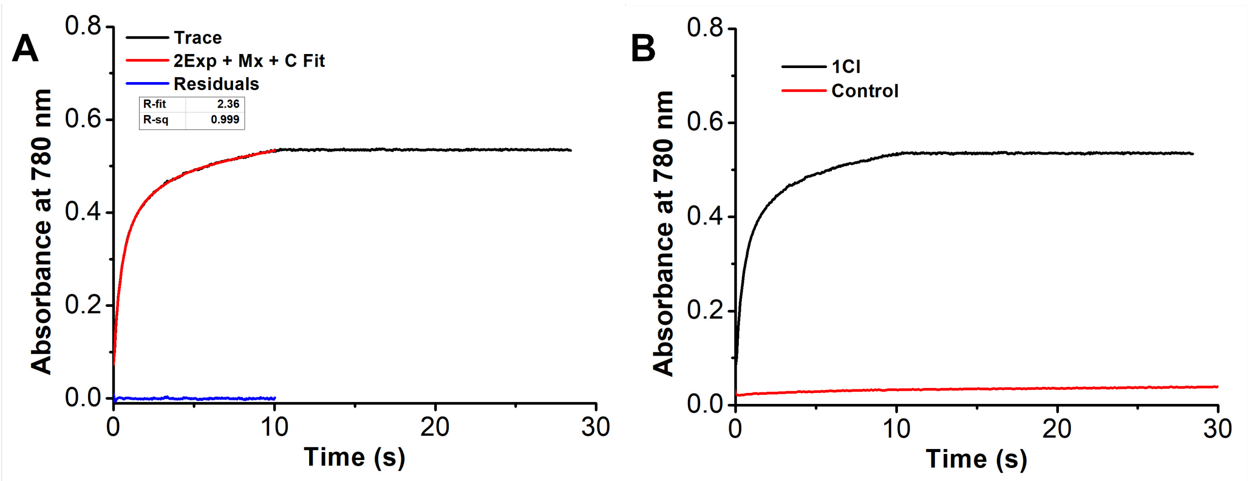

Figure S49. (A) Change in absorbance at 780 nm over time due to the formation of $[Cp^*{}_2Fe]^+$ by ORR catalyzed by **1Cl** with Cl_2AcOH (black trace), exampled of a 2Exp + Mx + C fit in Kinetic Studio 4.0 (red trace), and residual fit (blue trace). (B) Black trace from (A) and Cl_2AcOH only control (red trace, no **1Cl** present). Concentrations: **1Cl** (if present) = 10 µM, Cl_2AcOH = 10 mM, O_2 = 4.05 mM, $Cp^*{}_2Fe$ = 1 mM.

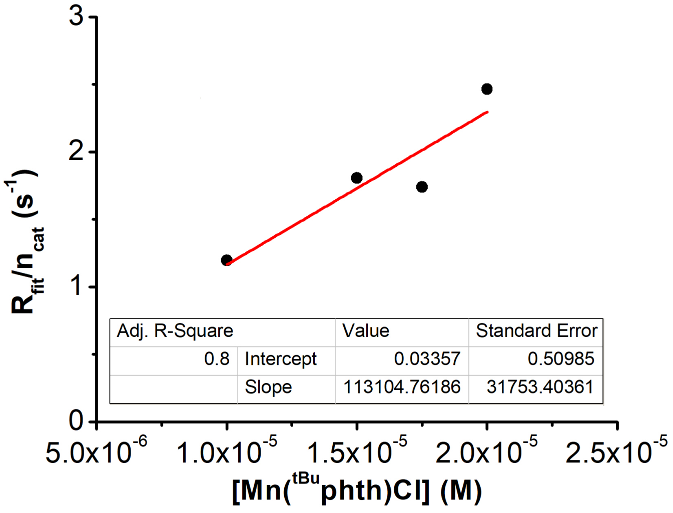

Figure S50. Calculated R_{fit}/n_{cat} values from stopped-flow spectrochemical experiments with Cl_2AcOH, O_2, and $Cp^*{}_2Fe$ with varying **1Cl** concentrations. Data were fit using Kinetic Studio 4.0 (2Exp + Mx + C); $n_{cat} = 2.0$. Concentrations: Cl_2AcOH = 10 mM, O_2 = 4.05 mM, $Cp^*{}_2Fe$ = 1 mM.

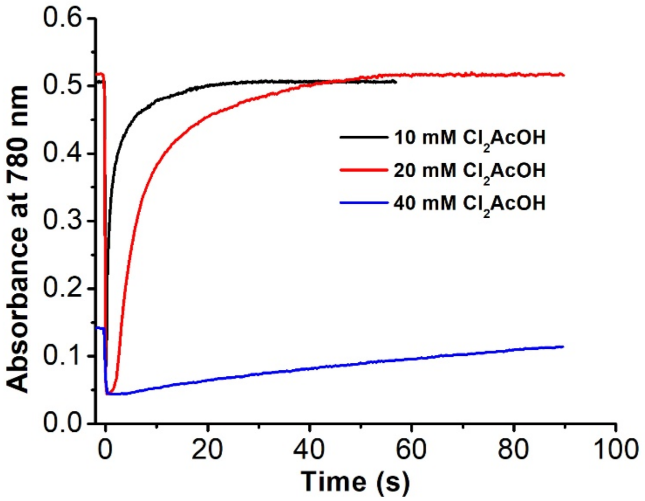

Figure S51. Change in absorbance at 780 nm over time due to production of $[Cp^*{}_2Fe]^+$ by ORR catalyzed by **1Cl** with O_2, $Cp^*{}_2Fe$, with varying Cl_2AcOH concentrations. Concentrations: **1Cl** = 10 μM, O_2 = 4.05 mM, $Cp^*{}_2Fe$ = 1 mM.

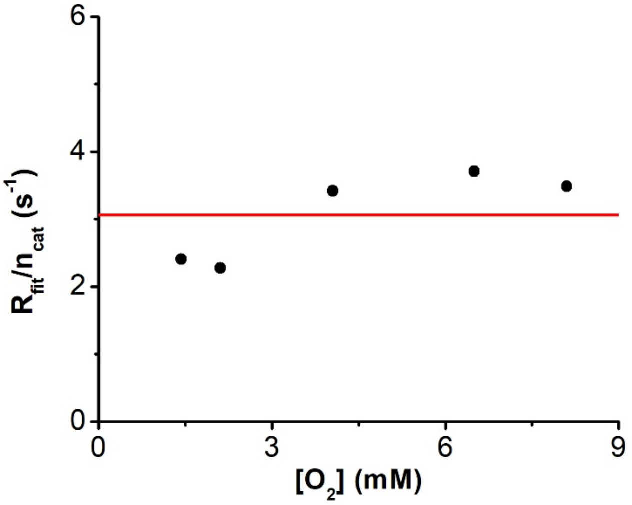

Figure S52. Calculated R_{fit}/n_{cat} values from stopped-flow spectrochemical experiments with Cl_2AcOH, **1Cl**, and $Cp^*{}_2Fe$ with varying O_2 concentrations. Data were fit using Kinetic Studio 4.0 (2Exp + Mx + C); n_{cat} = 2.0. Concentrations: **1Cl** = 10 µM, Cl_2AcOH = 10 mM, $Cp^*{}_2Fe$ = 1 mM.

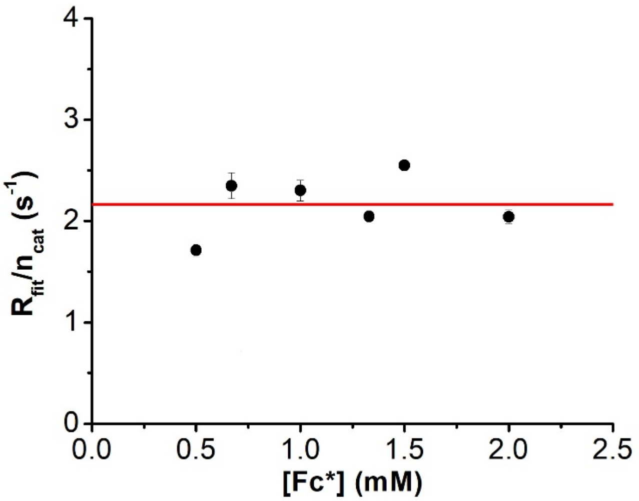

Figure S53. Calculated R_{fit}/n_{cat} values from stopped-flow spectrochemical experiments with **1Cl**, Cl_2AcOH, and O_2 with varying $Cp^*{}_2Fe$ concentrations. Data were fit using Kinetic Studio 4.0 (2Exp + Mx + C); n_{cat} = 2.0. Concentrations: **1Cl** = 10 µM, Cl_2AcOH = 10 mM, O_2 = 4.05 mM.

Calculation of Turnover Frequency. The turnover frequency per equivalent of Mn can be defined by the slope of the variable [**1Cl**] plot when there is a first-order dependence on **1Cl** (**Figure S53**).[9, 10] With Cl_2AcOH, TOF can be calculated using **Eq. S13** and the slope from **Figure S53.B.**

$$\frac{R_{fit}}{n_{cat}} = k_{cat}[\mathbf{1Cl}]^1[Cl_2AcOH]^{inverse} = TOF_{ORR}[\mathbf{1Cl}]^1 \qquad \textbf{(Eq. S13)}$$

$$TOF_{ORR} = 1.13 \times 10^6\ s^{-1}$$

Stopped-Flow with Cl_2AcOH/Cl_2AcO^- buffer

$$\frac{R_{fit}}{n_{cat}} = k_{cat}[\mathbf{1Cl}]^1[O_2]^1$$

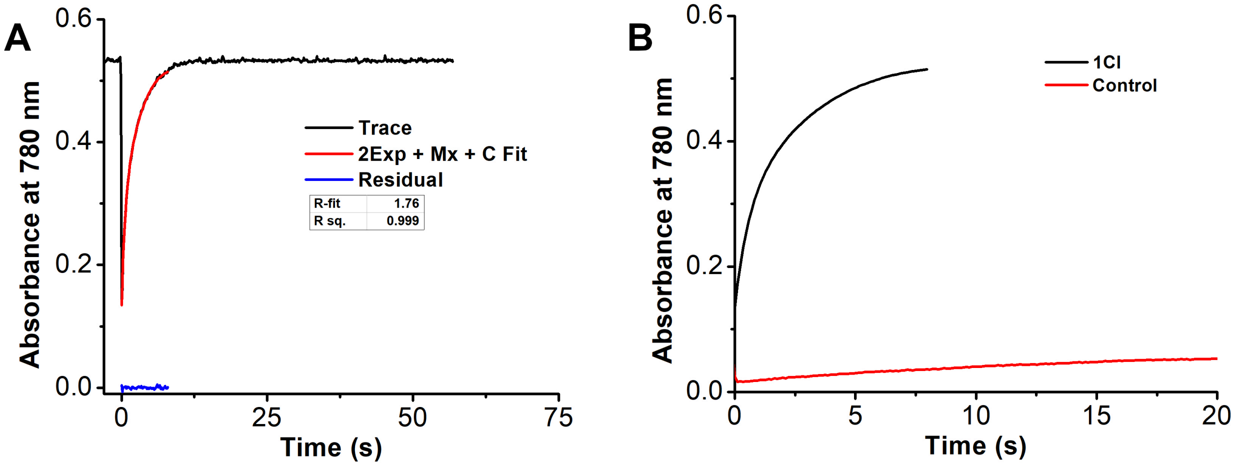

Figure S54. (A) Change in absorbance at 780 nm over time due to the formation of $[Cp^*_2Fe]^+$ by ORR catalyzed by **1Cl** with buffer (black trace), exampled of a 2Exp + Mx + C fit in Kinetic Studio 4.0 (red trace), and residual fit (blue trace). (B) Black trace from (A) and buffer only control (red trace, no **1Cl** present). Concentrations: **1Cl** (if present) = 10 µM, buffer = 40 mM, O_2 = 4.05 mM, Cp^*_2Fe = 1 mM.

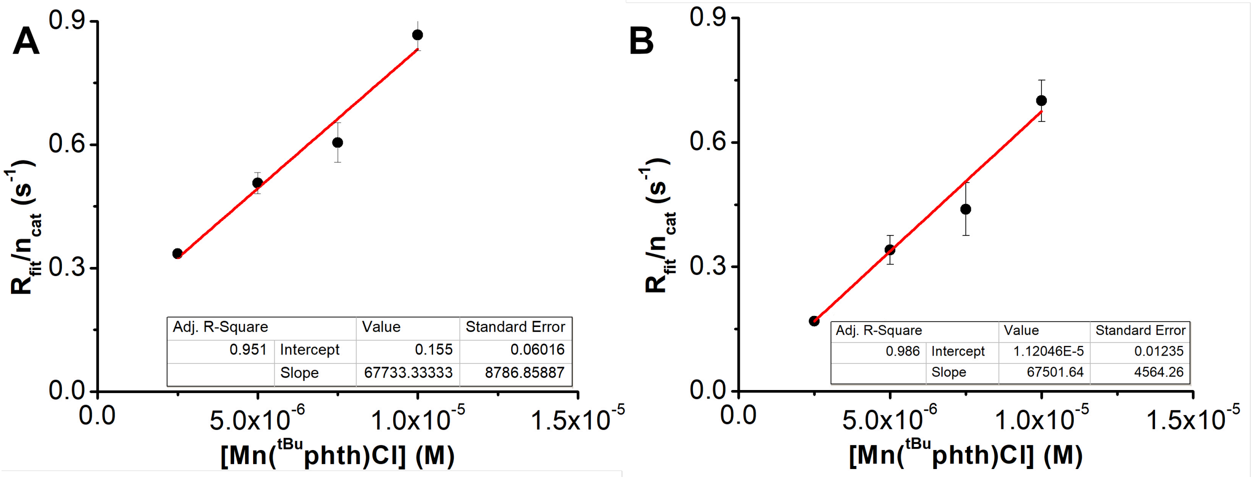

Figure S55. Calculated R_{fit}/n_{cat} values from stopped-flow spectrochemical experiments with buffer, O_2, and $Cp^*{}_2Fe$ with varying **1Cl** concentrations. (B) Corrected for background by subtracting the intercept from (A). Data were fit using Kinetic Studio 4.0 (2Exp + Mx + C); n_{cat} = 2.0. Concentrations: buffer = 40 mM, O_2 = 4.05 mM, $Cp^*{}_2Fe$ = 1 mM.

Calculation of Turnover Frequency. The turnover frequency per equivalent of Mn can be defined by the slope of the variable [**1Cl**] plot when there is a first-order dependence on [**1Cl**] (**Figure S55**).[9, 10] With Cl_2AcOH/Cl_2AcO^- buffer, TOF can be calculated using **Eq. S14** and the slope from **Figure S55.B**.

$$\frac{R_{fit}}{n_{cat}} = k_{cat}[\mathbf{1Cl}]^1[O_2]^1 = TOF_{ORR}[\mathbf{1Cl}]^1 \qquad \textbf{(Eq. S14)}$$

$$TOF_{ORR} = 6.75 \times 10^4\ s^{-1}$$

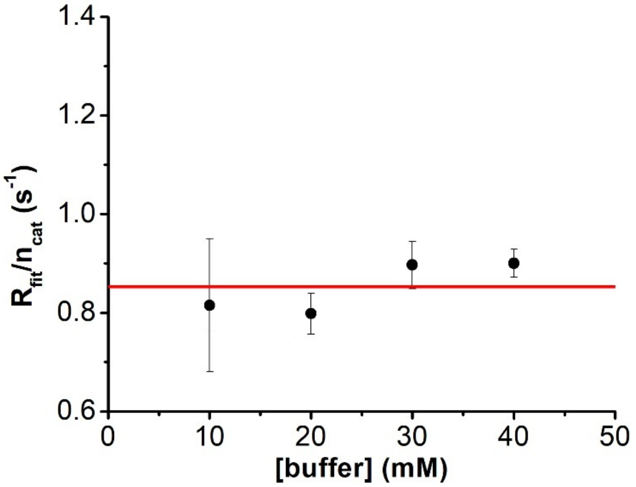

Figure S56. Calculated Rfit/ncat values from stopped-flow spectrochemical experiments with **1Cl**, O2, and Cp*2Fe with varying buffer concentrations. Data were fit using Kinetic Studio 4.0 (2Exp + Mx + C); n_{cat} = 2.0. Concentrations: **1Cl** = 10 μM, O_2 = 4.05 mM, Cp^*_2Fe = 1 mM.

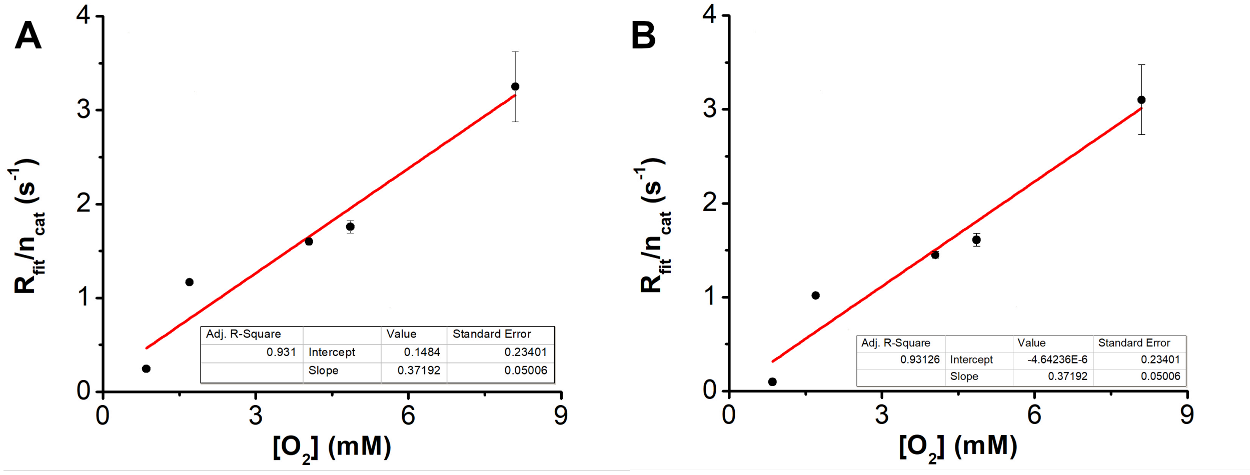

Figure S57. Calculated R_{fit}/n_{cat} values from stopped-flow spectrochemical experiments with buffer, **1Cl**, and Cp^*_2Fe with varying O_2 concentrations. (B) Corrected for background by subtracting the intercept from (A). Data were fit using Kinetic Studio 4.0 (2Exp + Mx + C); n_{cat} = 2.0. Concentrations: **1Cl** = 20 μM, buffer = 80 mM, Cp^*_2Fe = 1 mM.

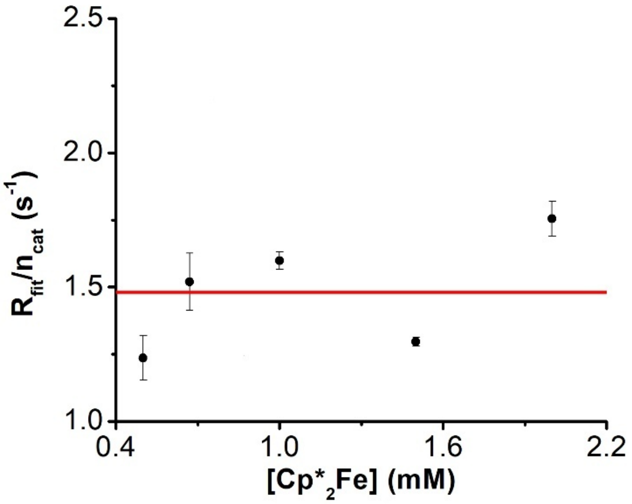

Figure S58. Calculated Rfit/ncat values from stopped-flow spectrochemical experiments with **1Cl**, buffer, and O_2 with varying $Cp^*{}_2Fe$ concentrations. Data were fit using Kinetic Studio 4.0 (2Exp + Mx + C); n_{cat} = 2.0. Concentrations: **1Cl** = 20 µM, buffer = 80 mM, O_2 = 4.05 mM.

Eyring Analysis of Buffered Conditions. Variable temperature stopped-flow kinetic analysis was performed to investigate the transition state of the mechanism of ORR catalyzed by **1Cl** with the buffered acid. The procedure for kinetic studies described above was followed, and the following temperatures were used: 15 °C, 20 °C, 25 °C, and 30 °C. The experiment was conducted 10 minutes after the temperature bath reached the desired temperature.

To generate an Eyring plot, **Eq. S15** was used to calculate k_{cat} for each trial; n_{cat} = 2.0:

$$k_{cat} = \frac{R_{fit}/n_{cat}}{[\mathbf{1Cl}][O_2]} \qquad \textbf{(Eq. S15)}$$

The k_{cat} values were averaged for each temperature before being used to create the Eyring plot (**Figure S59**).

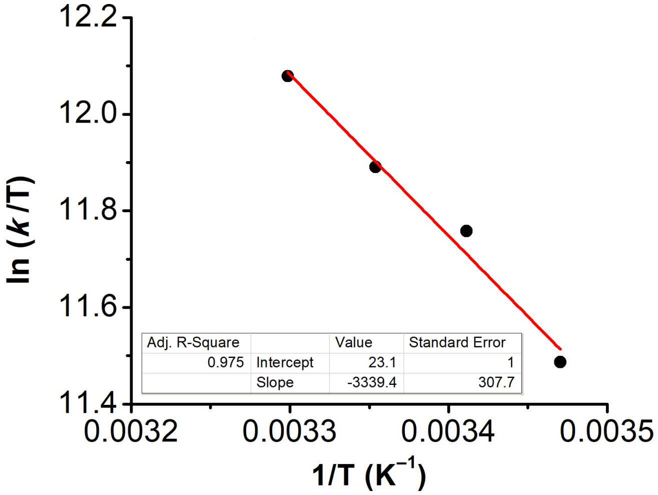

Figure S59. Eyring Plot for variable temperature stopped-flow kinetic data with **1Cl** and the buffer. Concentrations: **1Cl** = 10 μM, buffer = 40 mM, O_2 = 4.05 mM. Temperatures used = 15 °C, 20 °C, 25 °C, and 30 °C.

The Eyring Equation (**Eq. S16**); where R is the gas constant (8.314 J/mol•K), T is the temperature (K), k_B is the Boltzmann constant (1.381×10^{-23} J/K), and h is Planck's constant (6.626×10^{-34} J•s); along with the slope and y-intercept values from **Figure S59** were then used to determine the values for $\Delta H^{\ddagger}$ (**Eqs. S17-S18**) and $\Delta S^{\ddagger}$ (**Eqs. S19-S20**):

$$\ln\frac{k}{T} = \frac{-\Delta H^{\ddagger}}{R}\frac{1}{T} + \ln\frac{k_B}{h} + \frac{\Delta S^{\ddagger}}{R} \qquad \textbf{(Eq. S16)}$$

$$slope = \frac{-\Delta H^{\ddagger}}{R} \qquad \textbf{(Eq. S17)}$$

$$\Delta H^{\ddagger} = \frac{-slope}{R} \qquad \textbf{(Eq. S18)}$$

$$\Delta H^{\ddagger} = 27.8\ \frac{kJ}{mol}$$

$$y - intercept = \frac{\Delta S^{\ddagger}}{R} + ln\frac{k_B}{h} \qquad \textbf{(Eq. S19)}$$

$$\Delta S^{\ddagger} = \left[(y - int) - ln\frac{k_B}{h}\right]R \qquad \textbf{(Eq. S20)}$$

$$\Delta S^{\ddagger} = -5.49\ \frac{J}{mol \cdot K}$$

Spectrochemical Analysis

ORR Selectivity

The spectrochemical ORR selectivity of **1Cl** was determined using a $Ti(O)SO_4$ colorimetric assay.[3, 10]

Preparation of 0.1 M Ti(O)SO₄ Solution. As previously reported,[11] Ti(O)SO_4 (4.0 g, 25 mmol) was added to a 2.0 M solution of H_2SO_4 (26.65 mL H_2SO_4 in 250 mL DI H_2O) and heated until it was dissolved.

Preparation of H_2O_2 Calibration Curve. A calibration curve (**Figure S60**) was obtained through serial dilutions of a stock solution of urea•H_2O_2 (2 mM) in MeCN while monitoring the absorbance at 407 nm after the addition of Ti(O)SO_4. For each UV-vis sample, 2 mL of the urea•H_2O_2 solution was extracted with 10 mL of dichloromethane and 5 mL of DI H_2O. Then, 3 mL of the aqueous layer was transferred to a cuvette, and a UV-vis spectrum was obtained before and after the addition of 0.1 mL of the Ti(O)SO_4 solution. The spectrum before the addition of Ti(O)SO_4 was subtracted from the spectrum collected after the addition of the Ti(O)SO_4.

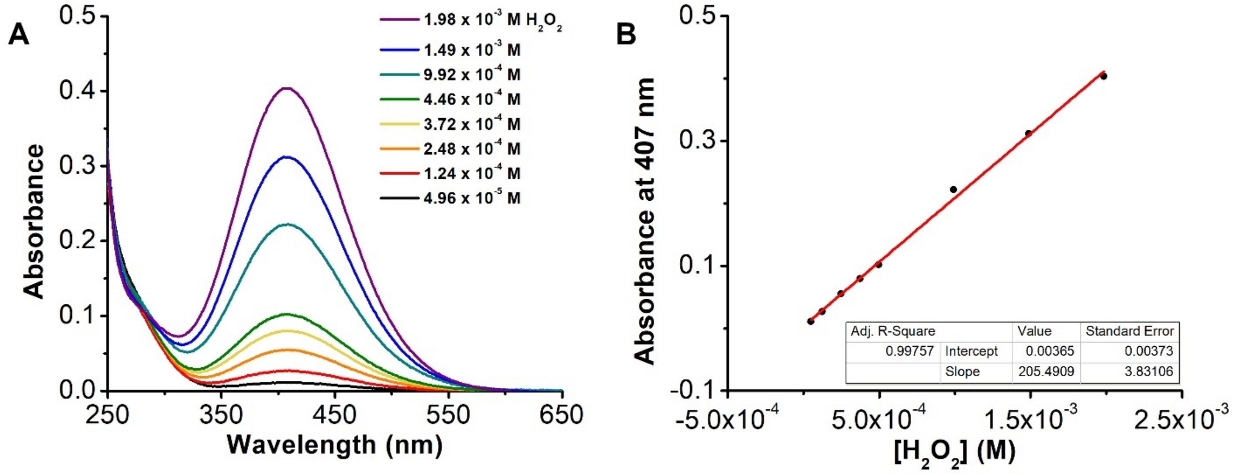

Figure S60. Calibration curve of H_2O_2 quantification for Ti(O)SO_4 colorimetric assay. (A) Serial dilution of urea•H_2O_2 in MeCN using the described extraction method. (B) Calibration curve made using the corrected absorbance at 407 nm.

ORR Product Quantification. Generally, solutions of 40 μM **1Cl** and 50 mM acid (ClAcOH, Cl_2AcOH/Cl_2AcO$^-$ buffer) or 40 mM acid (Cl_2AcOH) were sparged with O_2 and rapidly mixed in a 1:1 ratio with an Ar-saturated, 1 mM Cp^*_2Fe solution to a final volume of 8 mL (final concentrations: 20 μM **1Cl**, 25 or 20 mM acid, 0.5 mM Cp^*_2Fe solution, 4.05 mM O_2). At the indicated timepoints, 2 mL of the reaction solution were withdrawn and extracted with 10 mL of dichloromethane and 5 mL of water. Then, 3 mL of the aqueous layer was added to a cuvette, and a UV-vis spectrum of the solution was obtained before and after the addition of 0.1 mL of 0.1 M Ti(O)SO_4 solution. The calibration curve was used to establish **Eqs. S21-S23**, which were used to calculate the selectivity of H_2O_2 production.

$$O_2 + 2Cp^*{}_2Fe\ (0.5\ mM) + 2\ HA \xrightarrow{cat.[\mathbf{1Cl}]} H_2O_2\ (0.25\ mM) + 2[Cp^*{}_2Fe]^+ + 2A^- \quad \textbf{(Eq. S21)}$$

$$\text{Abs @ 407 nm (red trace)} - \text{Abs @ 407 nm (black trace)} = 205.5*[H_2O_2]_{exp} - 0.00365 \quad \textbf{(Eq. S22)}$$

$$\frac{[H_2O_2]_{exp}}{0.25\ mM\ H_2O_2} \times 100 = \%\ H_2O_2\ selectivity \quad \textbf{(Eq. S23)}$$

Table S2. Summary of spectrochemical ORR selectivity by **1Cl** with each acid.

	**% H_2O_2**	**n_{cat}**
**ClAcOH[a]**	96.5 ± 9.9	2.07
**Cl_2AcOH/Cl_2AcO^- Buffer[b]**	102.9 ± 6.7	2.0
**Cl_2AcOH[a]**	99.9 ± 6.6	2.0

[a] 20 μM **1Cl**, 25 mM acid, 4.05 mM O_2, 0.5 mM Cp^*_2Fe; [b] 20 μM **1Cl**, 20 mM acid, 4.05 mM O_2, 0.5 mM Cp^*_2Fe.

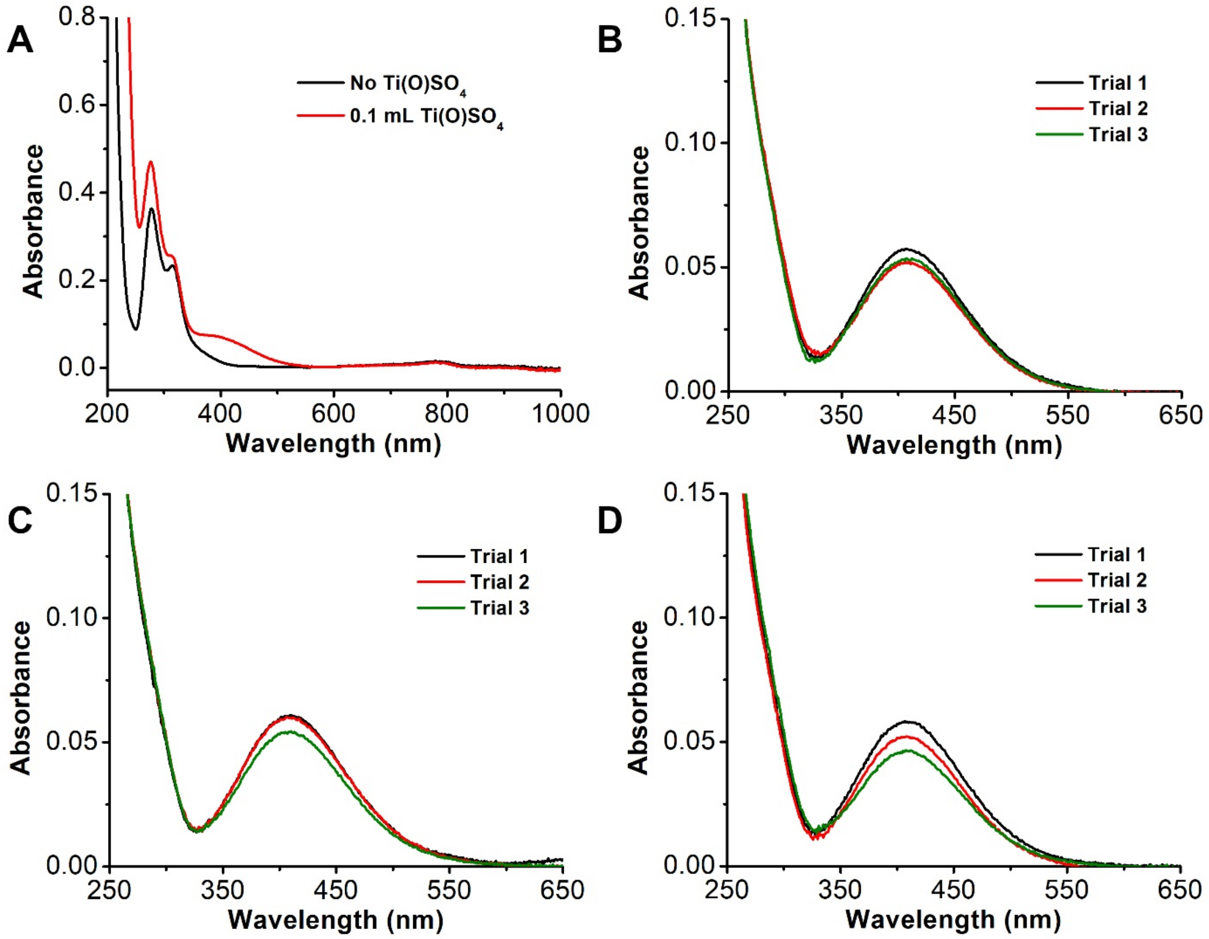

Figure S61. H_2O_2 product quantification of ORR by **1Cl** with ClAcOH after 30 s, 2 min, and 5 min. (A) UV-vis spectra of aqueous layer before (black) and after (red) the addition of 0.1 mL of 0.1 M $Ti(O)SO_4$ after 30 s. (B–D) Corrected spectra (red – black from A) for (B) 30 s, (C) 2 min, and (D) 5 min timepoints. Conditions: 20 μM **1Cl**, 25 mM ClAcOH, 4.05 mM O_2, 0.5 mM Cp^*_2Fe in MeCN.

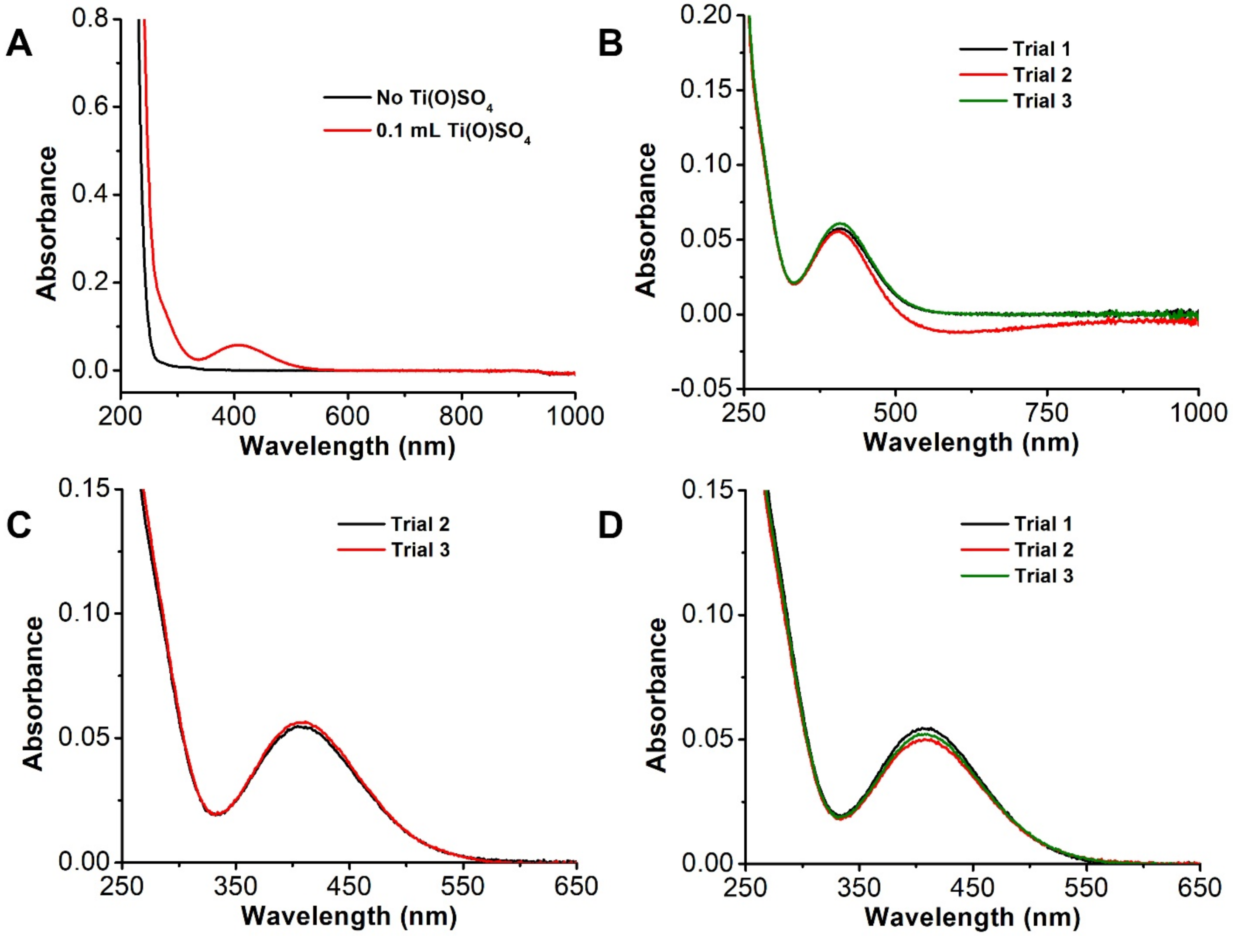

Figure S62. H_2O_2 product quantification of ORR by **1Cl** with Cl_2AcOH after 30 s, 2 min, and 5 min. (A) UV-vis spectra of aqueous layer before (black) and after (red) the addition of 0.1 mL of 0.1 M $Ti(O)SO_4$ after 30 s. (B–D) Corrected spectra (red – black from A) for (B) 30 s, (C) 2 min, and (D) 5 min timepoints. Conditions: 20 μM **1Cl**, 20 mM Cl_2AcOH, 4.05 mM O_2, 0.5 mM $Cp^*{}_2Fe$ in MeCN.

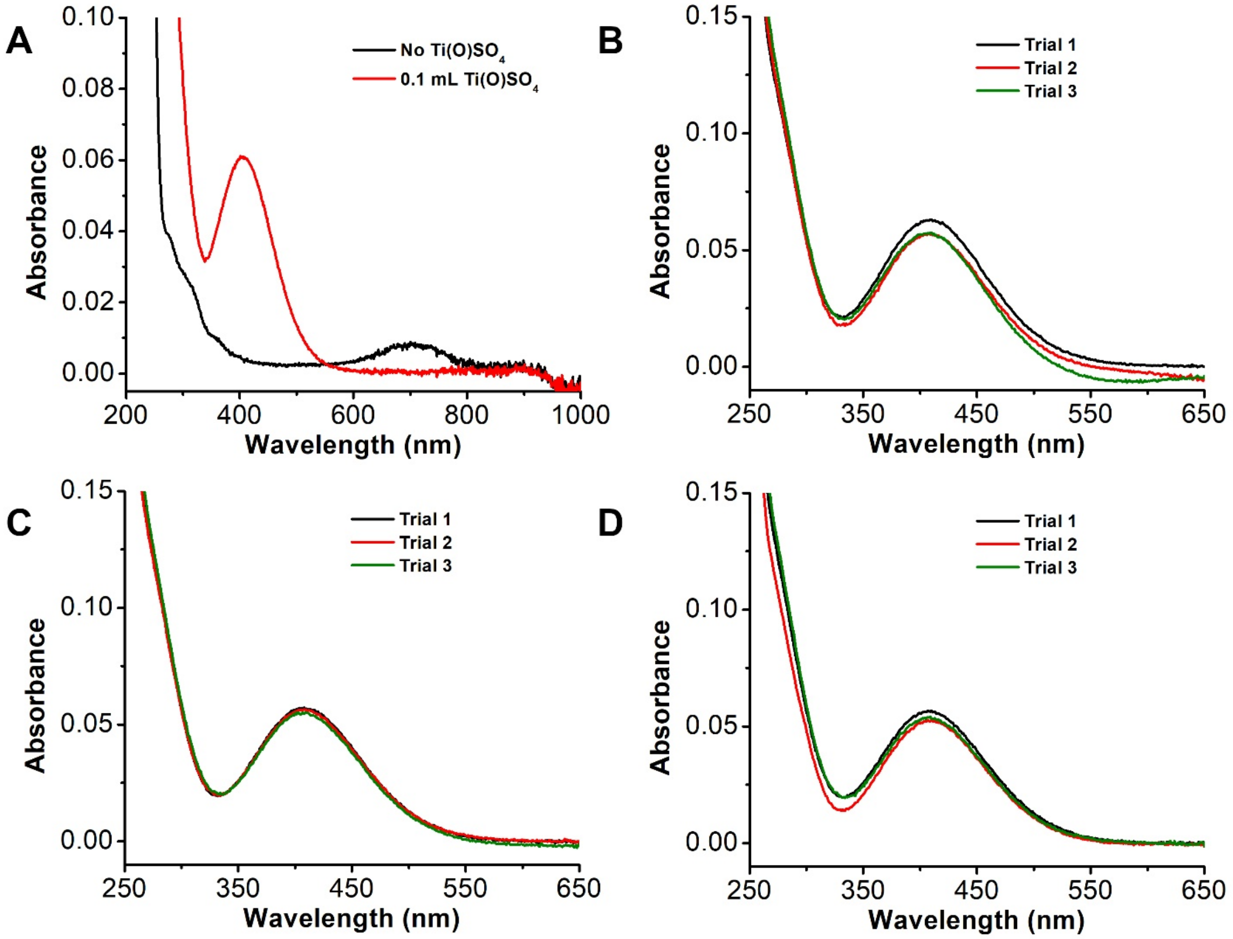

Figure S63. H_2O_2 product quantification of ORR by **1Cl** with buffer after 30 s, 1 min, and 2 min. (A) UV-vis spectra of aqueous layer before (black) and after (red) the addition of 0.1 mL of 0.1 M $Ti(O)SO_4$ after 30 s. (B–D) Corrected spectra (red – black from A) for (B) 30 s, 1 min, and 2 min timepoints. Conditions: 20 µM **1Cl**, 25 mM buffer, 4.05 mM O_2, 0.5 mM $Cp*_2Fe$ in MeCN.

H_2O_2 Stability Control Studies

Control studies were conducted to determine the stability of H_2O_2 in the presence of **1Cl**, acid, and O_2. Generally, solutions of **1Cl** (40 uM) and acid (50 mM for ClAcOH and buffer, and 40 mM for Cl_2AcOH) were sparged with O_2 for 30 s. Then, Ar-sparged urea•H_2O_2 solution (2 mM) was rapidly injected in a 1:1 ratio for final reaction concentrations of 20 uM **1Cl**, 25 or 20 mM acid (ClAcOH/buffer and Cl_2AcOH, respectively), 4.05 mM O_2, and 1 mM urea•H_2O_2. After 1 minute, 2 mL of the reaction solution was drawn into a syringe and transferred to 10 mL dichloromethane (DCM). The DCM/reaction mixture was extracted with 5 mL DI H_2O, and 3 mL of the aqueous layer was transferred to a cuvette. A UV-vis spectrum was obtained before and after 0.1 mL of the $Ti(O)SO_4$ solution was added. The absorbance value at 407 nm of the solution without $Ti(O)SO_4$ was subtracted from that with $Ti(O)SO_4$, and the amount of H_2O_2 present was calculated using **Eq. S22** (above). The % H_2O_2 recovered was calculated using **Eq. S24** (below).

$$\frac{[H_2O_2]_{exp.}}{0.001\ mM\ H_2O_2} \times 100 = \%\ H_2O_2\ recovered \quad \textbf{(Eq. S24)}$$

Table S3. Summary of H_2O_2 recovery for the stability control studies.

	**% H_2O_2 Recovered[a]**
**Cl_2AcOH[b]**	95.8 ± 2.1
**Buffer[c]**	78.3 ± 4.3
**ClAcOH[c]**	93.7 ± 4.4

[a] after 1 minute. [b] Conditions: 20 µM **1Cl**, 20 mM acid, 4.05 mM O_2, 1 mM urea•H_2O_2. [c] Conditions: 20 µM **1Cl**, 25 mM acid, 4.05 mM O_2, 1 mM urea•H_2O_2.

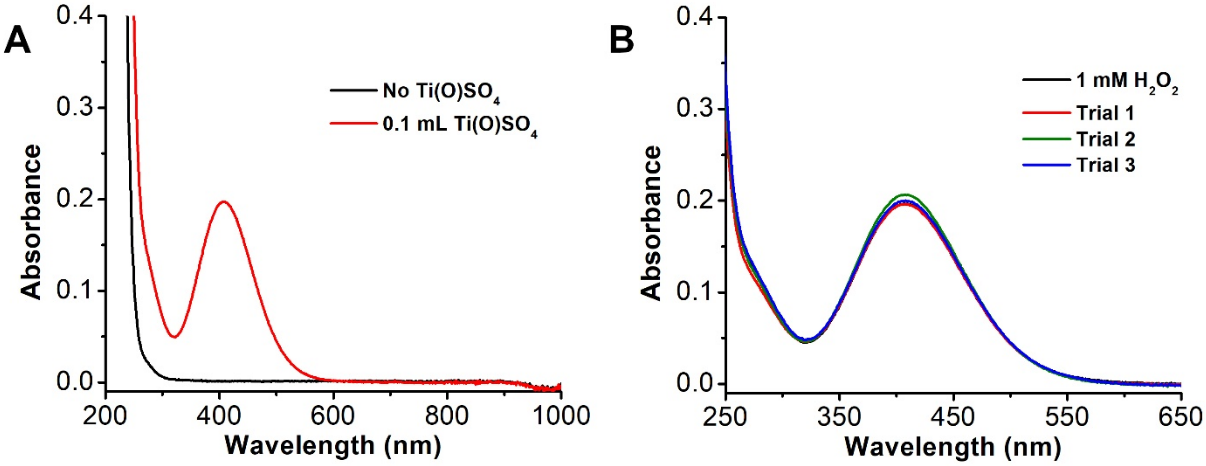

Figure S64. Stability test of urea•H_2O_2 in the presence of **1Cl**, Cl_2AcOH, and O_2. (A) UV-vis spectra of aqueous layer before (black) and after (red) the addition of 0.1 mL of 0.1 M Ti(O)SO_4 after 1 min. (B) Corrected spectra (red – black from A) for each trial. Conditions: 20 µM **1Cl**, 20 mM Cl_2AcOH, 4.05 mM O_2, 1 mM urea•H_2O_2 in MeCN.

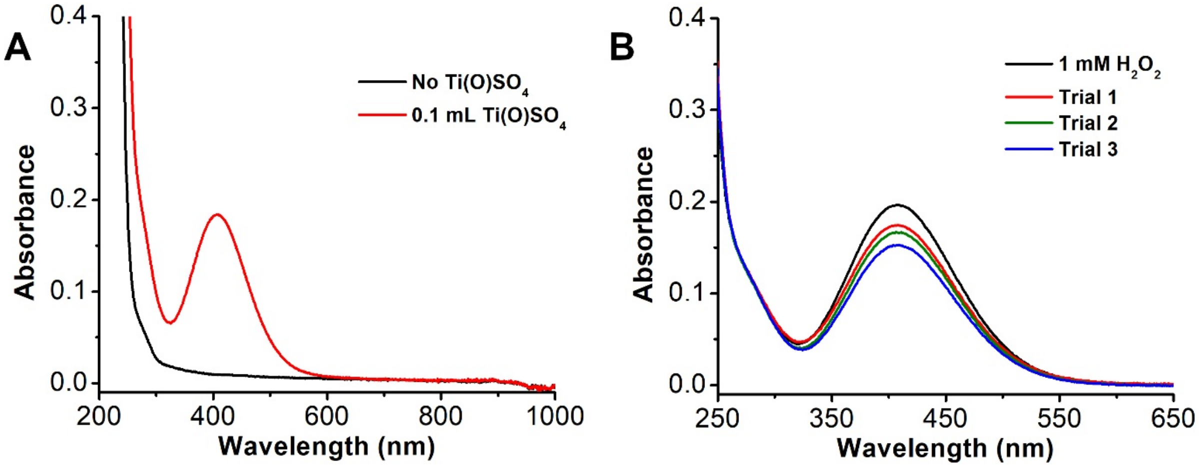

Figure S65. Stability test of urea•H_2O_2 in the presence of **1Cl**, buffer, and O_2. (A) UV-vis spectra of aqueous layer before (black) and after (red) the addition of 0.1 mL of 0.1 M Ti(O)SO_4 after 1 min. (B) Corrected spectra (red – black from A) for each trial. Conditions: 20 µM **1Cl**, 25 mM buffer, 4.05 mM O_2, 1 mM urea•H_2O_2 in MeCN.

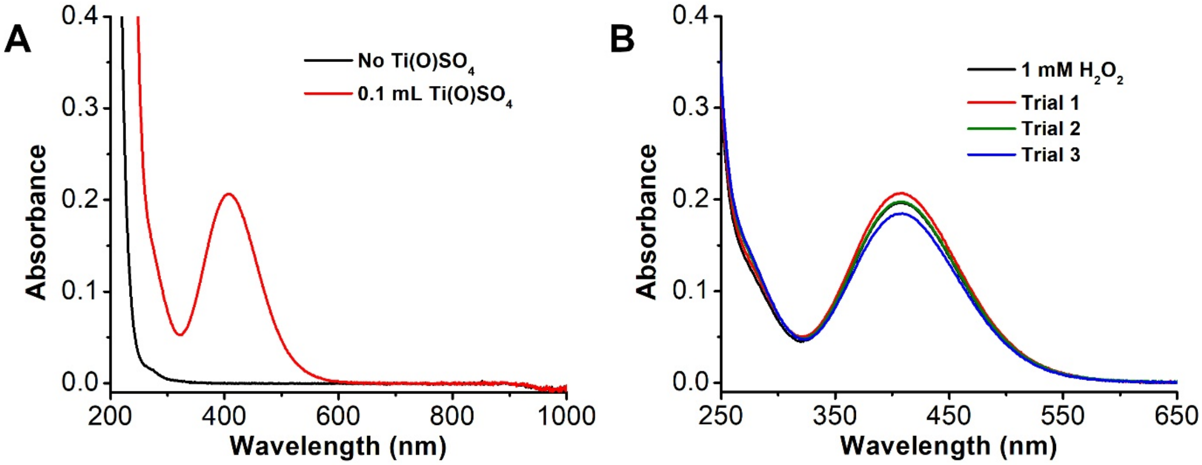

Figure S66. Stability test of urea•H_2O_2 in the presence of **1Cl**, ClAcOH, and O_2. (A) UV-vis spectra of aqueous layer before (black) and after (red) the addition of 0.1 mL of 0.1 M Ti(O)SO_4 after 1 min. (B) Corrected spectra (red – black from A) for each trial. Conditions: 20 µM **1Cl**, 25 mM ClAcOH, 4.05 mM O_2, 1 mM urea•H_2O_2 in MeCN.

Computational Methods

Geometry optimizations were performed without geometry constraints at the DFT level with the Gaussian 16 program, Rev B.01,[12] employing the hybrid functional B3LYP[13-16] and the def2-SVP basis set was used for all atoms.[17, 18] Dispersion and bulk solvent effects (acetonitrile = MeCN; ε = 35.688) were accounted for at the optimization stage, by using Grimme's D3 parameter set with Becke-Johnson (BJ) damping[19, 20] and the SMD continuum model,[21] respectively. The stationary points and their nature as minima (no imaginary frequencies) were characterized by vibrational analysis using the IGRRHO approach as implemented by default in the software package, which also produced enthalpy (H), entropy (S) and Gibbs energy (G) data at 298.15 K. The minima connected by a given transition state were determined by perturbing the transition states along the TS coordinate and optimizing to the nearest minimum. Free energies were corrected (ΔG_{qh}) to account for concentration effects and for errors associated with the harmonic oscillator approximation. Thus, according to Truhlars's quasi-harmonic approximation for vibrational entropy and enthalpy, all vibrational frequencies below 100 cm^{-1} were set to this value.[22] These anharmonic and concentration corrections were calculated with the Goodvibes code.[23] Concentrations were set at 0.001 M for all metal complexes, 0.004 M for O_2, 0.050 for acids, and 0.001 M for their homoconjugate pairs. Energies were refined with Orca 6.0[24-30] at the ωB97M-D4/def2-TZVPPD level.[17, 18, 31-44] The stability of the wavefunction and spin contamination were studied at the double-zeta level of theory. Reduction potentials from computational data were obtained according to our previous methodology by using the calculated free energy of reduction of the species of interest by [phenazine]$^-$, corrected to the experimental potential of phenazine reduction vs Fc^+/Fc.[45]

References

(1) Li, Q.; Batchelor-McAuley, C.; Lawrence, N. S.; Hartshorne, R. S.; Compton, R. G. Anomalous solubility of oxygen in acetonitrile/water mixture containing tetra-n-butylammonium perchlorate supporting electrolyte; the solubility and diffusion coefficient of oxygen in anhydrous acetonitrile and aqueous mixtures. *J. Electroanal. Chem.* **2013**, *688*, 328-335. DOI: 10.1016/j.jelechem.2012.07.039.

(2) Metz, J.; Schneider, O.; Hanack, M. Synthesis and Properties of Substituted (Phthalocyaninato)iron and -cobalt Compounds and Their Pyridine Adducts. *Inorg. Chem.* **1984**, *23*, 1065-1071. DOI: 10.1021/ic00176a014.

(3) Cook, E. N.; Flaxman, L. A.; Reid, A. G.; Dickie, D. A.; Machan, C. W. Acid Strength Effects on Dimerization during Metal-Free Catalytic Dioxygen Reduction. *J. Am. Chem. Soc.* **2024**, *146* (36), 24892-24900. DOI: 10.1021/jacs.4c05708.

(4) McCarthy, B. D.; Martin, D. J.; Rountree, E. S.; Ullman, A. C.; Dempsey, J. L. Electrochemical Reduction of Brønsted Acids by Glassy Carbon in Acetonitrile-Implications for Electrocatalytic Hydrogen Evolution. *Inorg. Chem.* **2014**, *53*, 8250-8261. DOI: dx.doi.org/10.1021/ic500770k

(5) Harraz, D. M.; Weng, S.; Surendranath, Y. Electrochemically Quantifying Oxygen Reduction Selectivity in Nonaqueous Electrolytes. *ACS Catalysis* **2023**, *13* (2), 1462-1469. DOI: 10.1021/acscatal.2c04564.

(6) Hooe, S. L.; Rheingold, A. L.; Machan, C. W. Electrocatalytic Reduction of Dioxygen to Hydrogen Peroxide by a Molecular Manganese Complex with a Bipyridine-Containing Schiff Base Ligand. *J. Am. Chem. Soc.* **2018**, *140*, 3232-3241. DOI: doi.org/10.1021/jacs.7b09027.

(7) Zhou, R.; Zheng, Y.; Jaroniec, M.; Qiao, S.-Z. Determination of the Electron Transfer Number for the Oxygen Reduction Reaction: From Theory to Experiment. *ACS Catal.* **2016**, *6*, 4720-4728. DOI: 10.1021/acscatal.6b01581.

(8) McKinnon, M.; Rochford, J. Principles of Electrocatalysis. In *Green Chemistry*, Török, B., Dransfield, T. Eds.; Elsevier, 2018; pp 695-727.

(9) Wasylenko, D. J.; Rodríguez, C.; Pegis, M. L.; Mayer, J. M. Direct Comparison of Electrochemical and Spectrochemical Kinetics for Catalytic Oxygen Reduction. *J. Am. Chem. Soc.* **2014**, *136*, 12544-12547.

(10) Cook, E. N.; Dickie, D. A.; Machan, C. W. Catalytic Reduction of Dioxygen to Water by a Bioinspired NonHeme Iron Complex via a 2+2 Mechanism. *J. Am. Chem. Soc.* **2021**, *143*, 16411-16418. DOI: 10.1021/jacs.1c04572.

(11) Anson, C. W.; Stahl, S. S. Cooperative Electrocatalytic O2 Reduction Involving Co(salophen) with p-Hydroquinone as an Electron−Proton Transfer Mediator. *J. Am. Chem. Soc.* **2017**, *139*, 18472-18475. DOI: doi.org/10.1021/jacs.7b11362.

(12) *Gaussian 16 Rev. B.01*; Frisch, M.J., Trucks, G.W., Schlegel, H.B., Scuseria, G.E., Robb, M.A., Cheeseman, J.R.; Scalmani, G.; Barone, V.; Petersson, G.A.; Nakatsuji, H.; Li, X.; Caricato, M.; Marenich, A.V.; Bloino, J., Janesko, B.G., Gomperts, R., Mennucci, B., Hratchian, H.P., Ortiz, J.V., Izmaylov, A.F., Sonnenberg, J.L., Williams-Young, D., Ding, F., Lipparini, F., Egidi, F., Goings, J., Peng, B., Petrone, A., Henderson, T., Ranasinghe, D., Zakrzewski, V.G., Gao, J., Rega, N., Zheng, G., Liang, W., Hada, M., Ehara, M., Toyota, K., Fukuda, R., Hasegawa, J., Ishida, M., Nakajima, T., Honda, Y., Kitao, O., Nakai, H., Vreven, T., Throssell, K., Montgomery Jr., J.A., Peralta, J.E., Ogliaro, F., Bearpark, M.J., Heyd, J.J., Brothers, E.N., Kudin, K.N., Staroverov, V.N., Keith, T.A., Kobayashi, R., Normand, J., Raghavachari, K., Rendell, A.P., Burant, J.C., Iyengar, S.S., Tomasi, J., Cossi, M., Millam, J.M., Klene, M., Adamo, C., Cammi, R., Ochterski, J.W., Martin, R.L., Morokuma, K., Farkas, O., Foresman, J.B., Fox, D.J. Gaussian, Inc., Wallingford CT (2016) GaussView 5.0. Wallingford, E.U.A.

(13) Becke, A. D. Density-functional thermochemistry. III. The role of exact exchange. *J. Chem. Phys.* **1993**, *98* (7), 5648-5652. DOI: doi:http://dx.doi.org/10.1063/1.464913.

(14) Lee, C.; Yang, W.; Parr, R. G. Development of the Colle-Salvetti correlation-energy formula into a functional of the electron density. *Phys. Rev. B* **1988**, *37* (2), 785-789.

(15) Vosko, S. H.; Wilk, L.; Nusair, M. Accurate spin-dependent electron liquid correlation energies for local spin density calculations: a critical analysis. *Can. J. Phys.* **1980**, *58* (8), 1200-1211. DOI: 10.1139/p80-159 (acccessed 2016/05/20).

(16) Stephens, P. J.; Devlin, F. J.; Chabalowski, C. F.; Frisch, M. J. Ab Initio Calculation of Vibrational Absorption and Circular Dichroism Spectra Using Density Functional Force Fields. *J. Phys. Chem.* **1994**, *98* (45), 11623-11627. DOI: 10.1021/j100096a001.

(17) Weigend, F.; Ahlrichs, R. Balanced basis sets of split valence, triple zeta valence and quadruple zeta valence quality for H to Rn: Design and assessment of accuracy. *Phys. Chem. Chem. Phys.* **2005**, *7* (18), 3297-3305, 10.1039/B508541A. DOI: 10.1039/B508541A.

(18) Weigend, F. Accurate Coulomb-fitting basis sets for H to Rn. *Phys. Chem. Chem. Phys.* **2006**, *8* (9), 1057-1065, 10.1039/B515623H. DOI: 10.1039/B515623H.

(19) Grimme, S.; Antony, J.; Ehrlich, S.; Krieg, H. A consistent and accurate ab initio parametrization of density functional dispersion correction (DFT-D) for the 94 elements H-Pu. *J. Chem. Phys.* **2010**, *132* (15), 154104. DOI: doi:http://dx.doi.org/10.1063/1.3382344.

(20) Grimme, S.; Ehrlich, S.; Goerigk, L. Effect of the damping function in dispersion corrected density functional theory. *J. Comput. Chem.* **2011**, *32* (7), 1456-1465. DOI: 10.1002/jcc.21759.

(21) Marenich, A. V.; Cramer, C. J.; Truhlar, D. G. Universal Solvation Model Based on Solute Electron Density and on a Continuum Model of the Solvent Defined by the Bulk Dielectric Constant and Atomic Surface Tensions. *J. Phys. Chem. B* **2009**, *113* (18), 6378-6396. DOI: 10.1021/jp810292n.

(22) Ribeiro, R. F.; Marenich, A. V.; Cramer, C. J.; Truhlar, D. G. Use of Solution-Phase Vibrational Frequencies in Continuum Models for the Free Energy of Solvation. *J. Phys. Chem. B* **2011**, *115* (49), 14556-14562. DOI: 10.1021/jp205508z.

(23) *Goodvibes v3.0.1*; 2019. https://doi.org/10.5281/zenodo.3346166.

(24) Neese, F. Software update: The ORCA program system—Version 5.0. *WIREs Computational Molecular Science* **2022**, *12* (5), e1606, https://doi.org/10.1002/wcms.1606. DOI: https://doi.org/10.1002/wcms.1606 (acccessed 2023/05/15).

(25) Izsák, R.; Hansen, A.; Neese, F. The resolution of identity and chain of spheres approximations for the LPNO-CCSD singles Fock term. *Molecul. Phys.* **2012**, *110*, 2413-2417. DOI: 10.1080/00268976.2012.687466.

(26) Izsák, R.; Neese, F. An overlap fitted chain of spheres exchange method. *J. Chem. Phys.* **2011**, *135*. DOI: 10.1063/1.3646921.

(27) Izsák, R.; Neese, F.; Klopper, W. Robust fitting techniques in the chain of spheres approximation to the Fock exchange: The role of the complementary space. *J. Chem. Phys.* **2013**, *139*. DOI: 10.1063/1.4819264.

(28) Neese, F. The ORCA program system. *WIREs Comput. Mol. Sci.* **2012**, *2*, 73-78. DOI: 10.1002/wcms.81.

(29) Neese, F. Software update: the ORCA program system, version 4.0. *WIREs Comput. Mol. Sci.* **2018**, *8:e1327*. DOI: 10.1002/wcms.1327.

(30) Neese, F.; Wennmohs, F.; Becker, U.; Riplinger, C. The ORCA quantum chemistry program package. *J. Chem. Phys.* **2020**, *152*. DOI: 10.1063/5.0004608.

(31) Garcia-Ratés, M.; Neese, F. Effect of the Solute Cavity on the Solvation Energy and its Derivatives within the Framework of the Gaussian Charge Scheme. *J. Comput. Chem.* **2020**, *41*, 922-939. DOI: 10.1002/jcc.26139.

(32) Helmich-Paris, B.; Souza, B. d.; Neese, F.; Izsák, R. An improved chain of spheres for exchange algorithm. *J. Chem. Phys.* **2021**, *155*. DOI: 10.1063/5.0058766.

(33) Neese, F. An improvement of the resolution of the identity approximation for the formation of the Coulomb matrix. *J. Comput. Chem.* **2003**, *24* (14), 1740-1747. DOI: 10.1002/jcc.10318.

(34) Neese, F. Definition of corresponding orbitals and the diradical character in broken symmetry DFT calculations on spin coupled systems. *J. Phys. Chem. Solids* **2004**, *65* (4), 781-785. DOI: 10.1016/j.jpcs.2003.11.015.

(35) Neese, F. The SHARK Integral Generation and Digestion System. *J. Comput. Chem.* **2022**, *44*, 381-396. DOI: 10.1002/jcc.26942.

(36) Neese, F.; Wennmohs, F.; Hansen, A.; Becker, U. Efficient, approximate and parallel Hartree-Fock and hybrid DFT calculations. A 'chain-of-spheres' algorithm for the Hartree-Fock exchange. *Chem. Phys.* **2009**, *356* (1-3), 98-109. DOI: 10.1016/j.chemphys.2008.10.036.

(37) Lehtola, S.; Steigemann, C.; Oliveira, M. J. T.; Marques, M. A. L. Recent developments in Libxc - A comprehensive library of functionals for density functional theory. *SoftwareX* **2018**, *7*. DOI: 10.1016/j.softx.2017.11.002.

(38) Caldeweyher, E.; Bannwarth, C.; Grimme, S. Extension of the D3 dispersion coefficient model. *J. Chem. Phys.* **2017**, *147*. DOI: 10.1063/1.4993215.

(39) Caldeweyher, E.; Ehlert, S.; Hansen, A.; Neugebauer, H.; Spicher, S.; Bannwarth, C.; Grimme, S. A generally applicable atomic-charge dependent London dispersion correction. *J. Chem. Phys.* **2019**, *150*. DOI: 10.1063/1.5090222.

(40) Caldeweyher, E.; Mewes, J.-M.; Ehlerta, S.; Grimme, S. Extension and evaluation of the D4 London-dispersion model for periodic systems. *Phys. Chem. Chem. Phys.* **2020**, *22* (16), 8499-8512. DOI: 10.1039/D0CP00502A.

(41) Rappoport, D. Property-optimized Gaussian basis sets for lanthanides. *J. Chem. Phys.* **2021**, *155* (12). DOI: 10.1063/5.0065611.

(42) Rappoport, D.; Furche, F. Property-optimized Gaussian basis sets for molecular response calculations. *J. Chem. Phys.* **2010**, *133* (13). DOI: 10.1063/1.3484283.

(43) Hellweg, A.; Hättig, C.; Höfener, S.; Klopper, W. Optimized accurate auxiliary basis sets for RI-MP2and RI-CC2 calculations for the atoms Rb to Rn. *Theor. Chem. Acc.* **2007**, *117*, 587-597. DOI: 10.1007/s00214-007-0250-5.

(44) Hellweg, A.; Rappoport, D. Development of new auxiliary basis functions of the Karlsruhe segmented contracted basis sets including diffuse basis functions (def2-SVPD, def2-TZVPPD, and def2-QVPPD) for RI-MP2 and RI-CC calculations. *Phys. Chem. Chem. Phys.* **2015**, *17*. DOI: 10.1039/C4CP04286G.

(45) Moreno, J. J.; Hooe, S. L.; Machan, C. W. DFT Study on the Electrocatalytic Reduction of CO_2 to CO by a Molecular Chromium Complex. *Inorg. Chem.* **2021**, *60* (6), 3635–3650. DOI: 10.1021/acs.inorgchem.0c03136.
